# Transcriptional programming using engineered systems of transcription factors and genetic architectures

**DOI:** 10.1038/s41467-019-12706-4

**Published:** 2019-10-21

**Authors:** Ronald E. Rondon, Thomas M. Groseclose, Andrew E. Short, Corey J. Wilson

**Affiliations:** Georgia Institute of Technology, School of Chemical & Biomolecular Engineering, Atlanta, GA USA

**Keywords:** Biochemistry, Biological techniques, Genetic engineering, Molecular engineering, Systems biology

## Abstract

The control of gene expression is an important tool for metabolic engineering, the design of synthetic gene networks, and protein manufacturing. The most successful approaches to date are based on modulating mRNA synthesis via an inducible coupling to transcriptional effectors. Here we present a biological programming structure that leverages a system of engineered transcription factors and complementary genetic architectures. We use a modular design strategy to create 27 non-natural and non-synonymous transcription factors using the lactose repressor topology as a guide. To direct systems of engineered transcription factors we employ parallel and series genetic (DNA) architectures and confer fundamental and combinatorial logical control over gene expression. Here we achieve AND, OR, NOT, and NOR logical controls in addition to two non-canonical half-AND operations. The basic logical operations and corresponding parallel and series genetic architectures represent the building blocks for subsequent combinatorial programs, which display both digital and analog performance.

## Abstract (web summary)

Successful approaches for controlling gene expression modulate mRNA synthesis by coupling it to inducible transcription effectors. Here the authors design 27 non-natural and non-synonymous transcription factors.

## Introduction

Biological computation, at its core, is the ability to engineer and develop systems capable of converting information (inputs) into a programmable gene expression (output(s))^1^. Gene regulation in biological systems can be viewed as a molecular computer. Namely, gene expression can be modeled as on–off states of Boolean (digital) logic, which can integrate multiple digital inputs into a desired output^2–4^. Currently, living cells can be programmed with genetic parts such as promoters, transcription factors, and metabolic genes^5–9^ to encode logical operations that integrate environmental and cellular signals^10–12^. Synthetic genetic logic gates have been engineered, including those capable of accomplishing AND, OR, and NOT functions^3,13–15^, which have been employed for pharmaceutical and biotechnological applications^16,17^. Moreover, combinations of such gates can be used to construct biological analogs of more advanced electronic circuits including switches^18–21^, logic^15,22,23^, memory^24,25^, pulse generators^26^, and oscillators^20,27–30^. Although logic in synthetic gene networks can be accomplished either at the transcriptional or translational levels, the former is more commonly employed in the development of synthetic gene networks via the use of transcription factors (TFs) to activate or repress genes of interest. Broadly speaking, TFs are DNA-binding proteins capable of blocking (or recruiting) RNA polymerase activity at the site of genetic promoters, and these functions can be combined in modular ways to engineer synthetic gene networks^31^. For the most part, early bacterial gene circuits were based on a core set of repressors, namely, TetR, LacI, and bacteriophage λ cI^15,30,32,33^, which have been extensively studied.

**Fig. 1:**
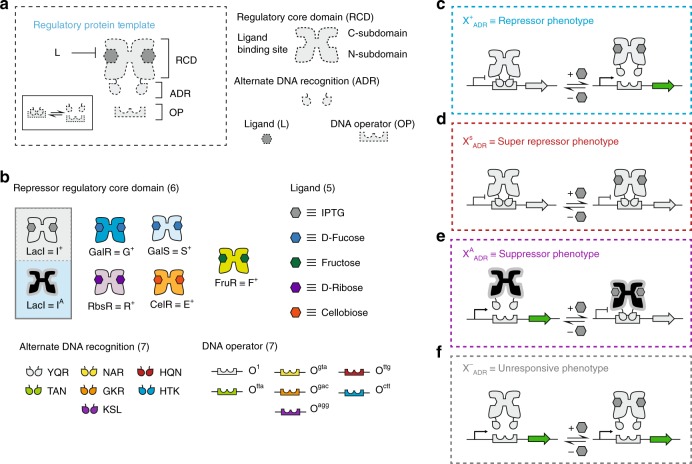
Workflow for generating X_ADR_ transcription factors. **a** Top panel illustrates the regulatory protein template. This system consists of three parts: a dimeric transcription factor (TF) along with its corresponding ligand (L), and its cognate DNA operator (OP). The interaction between the protein and its operator is modulated by binding of a complementary ligand and the system exchanges between the operator bound and unbound states (inset). The TF can be divided into two components, the regulatory core domain, or RCD and the (alternate) DNA recognition unit (ADR). The regulatory core domain can be further divided into the N- and C-subdomains, with the ligand binding domain at the cleft formed between the two. **b** Middle panel shows the discrete modules utilized in this work encompassing six regulatory core domains corresponding to five distinct ligands and seven Alternate DNA Recognition (ADR) units along with the corresponding DNA operator elements. The modules can be exchanged to yield a total of 42 combinations including 7 I^A^_ADR_ that have been previously reported. TF function can be classified into four distinct phenotypes. **c** The X^+^ or repressor phenotype inhibits gene expression in the absence of inducer by binding to the operator. Gene expression is induced when the repressor undergoes a conformational shift upon binding ligand. **d** The X^S^ or super-repressor phenotype cannot be induced either due to an inability to bind ligand, shift conformation, or dissociate from the operator sequence. **e** The X^A^ or suppressor phenotype allows gene expression in the absence of ligand. Whereas, binding of the ligand results in inhibition of gene expression. **f** The X^−^ or nonfunctional phenotype is incapable of inhibiting gene expression either from an inability to properly assemble or associate with its cognate operator

The lactose repressor (LacI) is a workhorse in the field of synthetic biology. LacI or I^+^_YQR_ (Fig. 1) is a canonical molecular switch, serving as the central regulatory protein in the lac operon of *Escherichia coli (E. coli)*. Under normal cellular conditions, LacI will bind to the O^1^ operator site and prevent transcription of downstream genes by physically blocking and compromising the activity of RNA polymerase^34^. In the presence of the chemical signal isopropyl-β-d-thiogalactoside or IPTG (a nonhydrolyzable analog of the natural inducer 1,6-allolactose) LacI undergoes a conformational shift that results in decreased affinity for its cognate DNA operator. This event allows transcription of the downstream gene to proceed^35^. Although the structure of the 360 amino acid LacI protein can be divided into several (sub)domains, for simplicity we will group them into the DNA-binding domain (DBD) and the regulatory core domain (RCD).The first 60 residues constitute the N-terminal DBD containing the helix-turn-helix motif, which aids in the recognition of the O^1^ operator region. Residues 61–330 constitute the RCD, which encompasses the regions responsible for dimer assembly (C-subdomain), ligand binding, and for mediating and propagating the allosteric signal across the protein (N-subdomain). The functional unit of LacI is a dimer, however residues 331–360 make up the C-terminal tetramerization domain which facilitates the dimerization of two functional units^36–39^.

LacI is part of the larger LacI/GalR family of transcriptional repressors that regulate sugar metabolism in *E. coli* and other organisms^40^. Most members of this family are structurally similar and several respond to their respective inducers in much the same way LacI responds to IPTG. In recent years, a library of chimeric repressors has been engineered by replacing the RCD of LacI with regulatory core domains from homologous LacI/GalR family members. This has generated LacI-like transcription factors capable of regulating the *lac* promoter via the O^1^ operator, while still responding to the homolog’s corresponding natural inducer^41–43^. Moreover, the DBD of LacI has previously been modified to achieve alternate DNA recognition (ADR) via the introduction of the point mutations Y17T, Q18A, R22N (i.e., the TAN mutation^44^) to create functionally orthogonal repressors which can be used to generate a variety of biological AND gates. For example, Shis et al. used several LacI/GalR chimeras to construct parallel AND gates, which used multiple TFs simultaneously with minimal cross-talk^45^.

Efforts in recent years have been made to expand the set of transcription factors that can be used toward the design of genetic circuits^46–49^. These efforts are critically important because the development and discovery of non-natural transcription factors will facilitate an increase in the size and sophistication of synthetic gene networks, and expand the capabilities of achieving biological analogs of basic Boolean logical operations^1,50^. In this work we demonstrate a modular design approach capable of engineering non-natural transcription factors with orthogonal ligand response and non-natural alternate DNA recognition or X^+^_ADR_ (Fig. 1). These transcriptional regulators are then used to develop the basic logical operations AND, OR, NOT and NOR in a manner that complements existing technologies with similar functionality^1,45,47,50,51^. The ability to exert ligand (signal) control directly at the transcription factor level eliminates the need for ligand inducible promoters or the need to use sensors independent of the logical circuitry^52^. Finally, pairing these X^+^_ADR_ repressors with our previously engineered collection of LacI suppressors (I^A(X)^_YQR_ and I^A(X)^_ADR_^53,54^) allows for the development of combinatorial and non-canonical logical operations. Collectively, the engineered TFs and corresponding genetic architectures lay the foundation for the development of a nascent biological (non-natural) programming language.

## Results

### Engineering workflow for non-natural transcription factors

In a previous study, we established a protein engineering strategy to confer alternate DNA-binding functions in the LacI scaffold^53^. Here we extended this workflow to simultaneously vary the RCD and the ADR (Fig. 1a, b). In brief, we adapted 5 disparate LacI/GalR regulatory core domains (CelR ≡ E, FruR ≡ F, GalR ≡ G, GalS ≡ S, and RbsR ≡ R) with six non-natural DNA-binding domains (NAR, HQN, TAN, GKR, HTK, and KSL), plus one naturally occurring domain (YQR) from LacI (Supplementary Fig. 1). Collectively, this resulted in a RCD-ADR combinatorial design space of 35 putative transcription factors (Supplementary Fig. 2). In principle, each engineered transcription factor binds to a single non-natural DNA operator (O^gta^, O^tta^, O^gac^, O^ctt^, and O^agg^ - respectively), while the YQR DNA-binding domain is complemented by the native O^1^ (or symmetric O^sym^) DNA operator. Mechanistically, when a given DNA operator is in proximity to a promoter element, transcription factor binding (to operator DNA) interferes with RNA polymerase function, inhibiting transcription (Fig. 1c, e). We postulated that each putative transcription factor adapted with a given ADR unit would retain the parental wild-type repressor phenotype (i.e., X^+^_ADR_, where X = G, E, R, S, and F) when paired with a cognate DNA operator element (Fig. 1c). Alternate DNA-binding domains and cognate operators were selected from a pool of engineered (non-natural) systems previously reported^55^, in which the DNA-binding motif and the cognate operator were simultaneously evolved and adapted via the wild-type LacI (I^+^) regulatory core domain. Out of the ~200 functional alternate DNA-binding domains and cognate non-natural operators, we selected the 6 ADR-operator sets for this study based on the performance metrics observed in our previous study, Rondon and Wilson^53^. In the aforementioned study, we adapted a collection of engineered LacI suppressors (Fig. 1e) with 6 non-natural ADR. The 6 ADR (plus YQR) were functional in both repressor (I^+^) and suppressor (I^A^) phenotypes. The wild-type YQR/O^1^ set was selected for this study to enable the transcription factors created in this work to function in cooperation with the foundational antilacs (suppressors) engineered in Richards et al.^54^. Likewise, the 35 putative X^+^_ADR_ variants tested in this study were selected to function as complementary systems to the suppressors adapted with ADR from our previous study^53^.

### Performance of engineered transcription factors

**Fig. 2:**
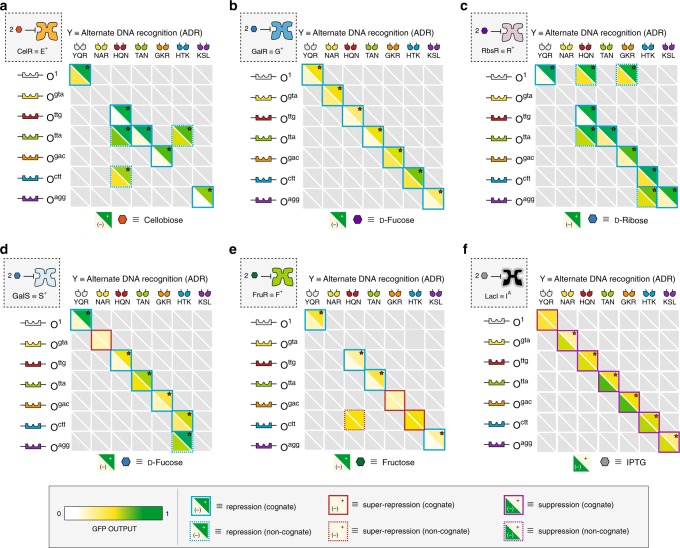
Repression matrices for the regulatory core domains equipped with ADR units. **a** Cellobiose Repressor, CelR ≡ E, **b** Galactose Repressor, GalR ≡ G, **c** Ribose Repressor, RbsR ≡ R, **d** Galactose Isorepressor, GalS ≡ S, **e** Fructose Repressor, FruR ≡ F and **f** a representative antilac I^A(1)^. The ADR DNA-binding domains are displayed along the top of the table and their corresponding operators are shown along the left. The bottom left triangle shows (OD_600_ normalized) GFP output in the absence of inducer, while the top right shows GFP output in the presence of 10 mM of the inducer corresponding to a given RCD. Red stars denote a statistically significant difference between the two states at *α* = 0.001 level using a one-tailed Student’s *t*-test. Values correspond to the mean of *n* = 12 biological replicates. Scale bar reference for GFP output are all scaled to the same absolute values (inset, bottom). Phenotype is denoted by the color of bounding box in accordance with Fig. 1. TF-operator pairs along the diagonal are classified as interactions between the ADR and a cognate operator (solid boxes). Dashed boxes indicates promiscuity in DNA recognition. Any repressor-operator combination that was incapable of repression (X^-^ phenotype) is denoted in gray

At the outset, we evaluated each of the 35 putative transcription factors paired with a single cognate DNA operator (i.e., data along the diagonals in Fig. 2a–e, summarized in Supplementary Fig. 3). In this system, a given DNA operator was located downstream (proximal) to a promoter element, and upstream to a green fluorescent protein (GFP) reporter (Supplementary Fig. 4). Provided that the related engineered transcription factor can functionally pair with the DNA operator, GFP can be regulated. In this experiment, fluorescence was measured in the presence and absence of a given effector ligand via a micro-well plate assay to determine phenotype and performance characteristics. Out of the 35 chimera, 27 functioned as cognate repressors X^+^ (Figs. 1c and 2a–e). Three putative transcription factors bound to cognate operator DNA, but were unresponsive to inducer ligands, and were designated as super repressors X^s^ (Figs. 1d and 2d, e). Five transcription factors remained unbound to related operator DNA, plus or minus effector ligand, and were classified as unresponsive X^−^ (Figs. 1f and 2a, c–e). Out of the 210 non-cognate TF-operator pairs (i.e., off-diagonal combinations in Fig. 2a–e) 201 were unresponsive (X^−^) in phenotype (Supplementary Fig. 5, and Supplementary Note. 1). Out of the nine non-cognate systems that interacted with operator DNA, eight were designated as X^+^, and one system produced the X^s^ phenotype.

**Fig. 3:**
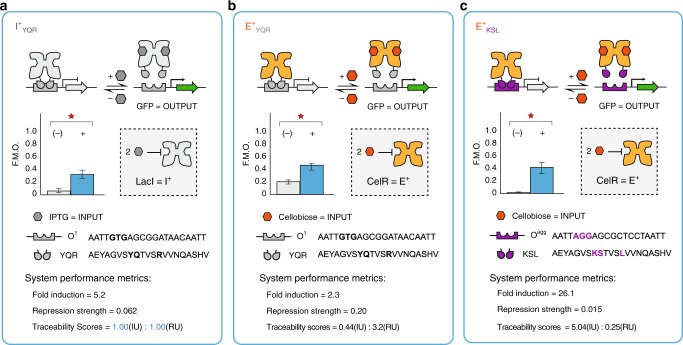
Performance cards for select repressor-operator combinations. **a** The I^+^_YQR_|O^1^ set was designated as the reference system with traceability scores set to 1. **b** The E^+^_YQR_|O^1^ and **c** E^+^_KSL_|O^agg^ were selected as relative analog and digital systems, respectively. The context for each performance card displaying the genetic architecture for performance reporting, the properties of the reporter plasmid, the cellular chassis chosen, as well as the details for F.M.O. (Fraction of Maximum Output), IU (Induction Units), RU (Repression Units), and Traceability Scores are given in the supplementary information (Supplementary Fig. 6 and Supplementary Note 2)

The gene regulator LacI (I^+^_YQR_) has successfully been used throughout synthetic biology. Accordingly, we used I^+^_YQR_ paired with O^1^ operator DNA (I^+^_YQR_|O^1^) as a reference system to score the relative performance of the engineered transcription factors observed in this study (Fig. 3). Thus, the performance of a functional repressor-operator set can be classified as an analog-like or digital-like process, relative to the I^+^_YQR_|O^1^ reference system (Fig. 3a, b, Supplementary Fig. 6 and Supplementary Note. 2). For example, the E^+^_YQR_|O^1^ process had a smaller fold induction and weaker repression strength (i.e., DNA interaction minus the ligand), relative to the reference system. Accordingly, the E^+^_YQR_|O^1^ system was designated as a more analog process. In contrast, the E^+^_KSL_|O^agg^ system can be classified as a more digital process, given the higher relative fold induction and stronger repression strength (Fig. 3c). A marked difference in the maximum expression for each promoter-operator combination was observed (Supplementary Fig. 3). To address this variation, we normalized and evaluated each repressor-operator pair relative to LacStop controls (Supplementary Fig. 3) under the same conditions. The normalized performance was reported as a Fraction of Maximum Output (F.M.O.), and allowed for a direct comparison of performance characteristics (i.e., repression strength and fold induction) across the design space (Supplementary Fig. 6 and Supplementary Note. 2). In summary, a total of 35 repressor systems (i.e., 27 cognate and 8 non-cognate) were identified in this assessment. Select repressor-operator systems from this collection were used to construct logical genetic programs in the subsequent sections.

### Genetic architectures used to direct transcription factors

**Fig. 4:**
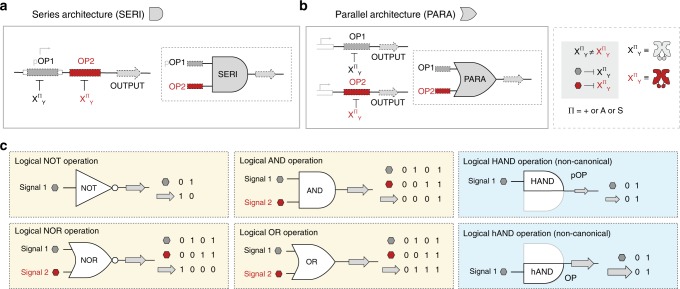
Genetic architectures and biological truth tables for logical operations. **a** In the series architecture (SERI), two operators are employed, each acted on by a transcription factor with a different ADR unit. The first operator (pOP1) lies within the promoter element, flanked by the −35 and −10 hexamers in the core position, while the second operator (OP2), lies downstream in the proximal position. **b** In the parallel architecture (PARA), a single operator controls a single output, but multiple outputs exist on a given plasmid. **c** Canonical logical operations (yellow boxes) along with corresponding truth tables. Gray and red hexagons represent distinct inputs selected from the five ligands in this work. Non-canonical logical operations (blue boxes) stemming from the series architecture. When the core operator is controlled, the resulting operation is denoted a HAND, while controlling the proximal operator leads to a hAND

To facilitate the development of logical gene control, we employed two fundamental genetic structures to support any pair of DNA operators. At the outset we composed genetic architectures that configured any two DNA operators in parallel or in series (Fig. 4a, b). The parallel (PARA) configuration consisted of two channels, each channel containing one DNA operator located downstream of the promoter element, but upstream of the GFP reporter gene or other designated output (Fig. 4b and Supplementary Fig. 4). The series (SERI) configuration was inspired by the architectures developed by Elowitz et al.^56^ in which two DNA operators were placed in tandem upstream of a given reporter gene. In our SERI architecture, the first DNA operator was intercalated within the promoter element and was designated as the core position, whereas the second DNA operator was located downstream of the promoter in the proximal position (Fig. 4a and Supplementary Fig. 4). The SERI and PARA genetic architectures can support any combination of two DNA operators (i.e., non-natural or natural), and when paired with engineered transcription factors can result in fundamental and non-canonical logical operations to regulate gene expression (Fig. 4c).

### Boolean logical operations NOT, AND, and OR

**Fig. 5:**
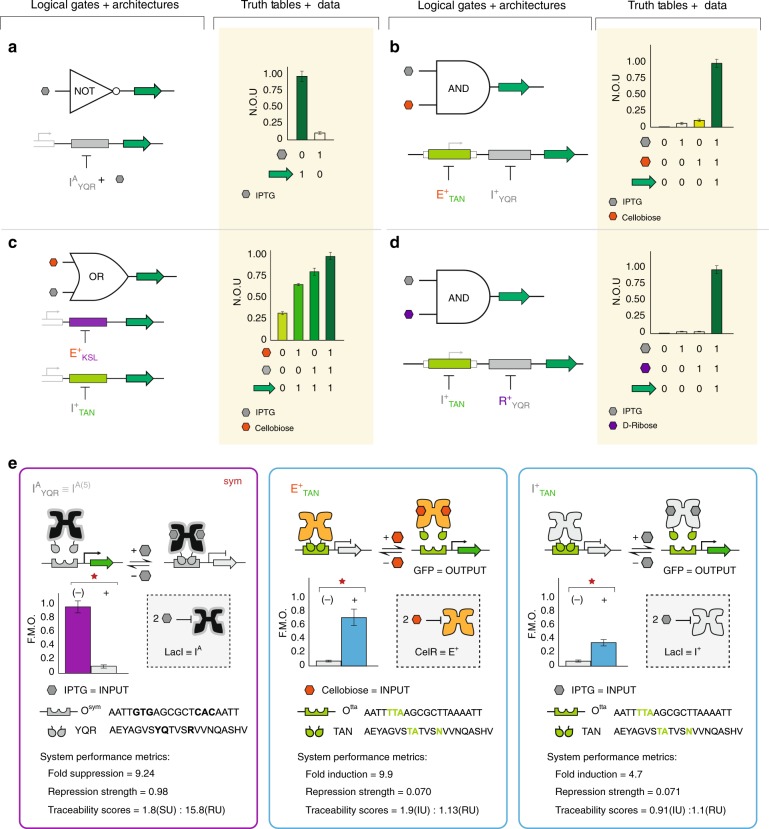
Biological logical gates NOT, AND, and OR. **a** An engineered LacI suppressor (antilac) represents a unary NOT gate, the output is OFF when IPTG is present. *Y*-axis is given as normalized output units or N.O.U. This is calculated by taking GFP fluorescence (485ex., 510em.) normalized to OD_600_, which is further normalized to the maximum output for each experiment. Each value corresponds to the mean of *n* = 6 biological replicates and error bars indicate the 95% confidence interval (95% CI), or approximately two times the standard error of the mean (s.e.m.). **b** A biological AND gate achieved via the SERI architecture utilizing two X^+^_ADR_ with orthogonal DNA recognition and ligand response. Values correspond to *n* = 18 biological replicates. **c** Biological OR function achieved through PARA architecture where each copy of GFP is controlled by a distinct X^+^_ADR_. Values correspond to *n* = 12 biological replicates. **d** Another iteration of a biological AND gate utilizing distinct input signals, via two non-natural transcription factors. Values correspond to *n* = 18 biological replicates. **e** Transcription factor operator pairs utilized in logic gates (**a**–**d**), along with their performance characteristics. For performance cards, values represent the mean of *n* = 12 biological replicates. Data in the cards have been normalized relative to the maximum value of 20,000 GFP/OD_600_ a.u. Note: the I^A^ suppressor used in the representative NOT gate is I^A(5)^_YQR_, created in our previous report (Rondon and Wilson)^53^. Source data are provided as a Source Data file, and individual data points overlaid as dot plots can be found in Supplementary Fig. 7

Using the aforementioned genetic architectures, we constructed three basic logical operations that leveraged the engineered repressors (X^+^_ADR_) developed in this study, and complementary antilac suppressors (I^A^_ADR_) from our previously reported systems^53^ (Fig. 5a–c). Namely, we constructed NOT, AND, and OR logical operations composed of engineered (non-natural) transcription factors and cognate operators arranged in PARA or SERI configurations. The representative NOT gate was composed of a single PARA channel configured with an O^sym^ DNA operator and cognate I^A^_YQR_ suppressor (Fig. 5a, e and Supplementary Fig. 7a). O^sym^ is an O^1^ variant in which the right-half of the operator has been symmetrized with the left to form a proper palindrome (Fig. 5e and Supplementary Fig. 6). In the absence of the effector ligand IPTG, I^A^_YQR_ cannot form a complex with O^sym^ DNA, thus GFP was produced. In contrast, upon the addition of 10 mM IPTG, the engineered antilac suppressor bound to operator DNA and suppressed GFP production. Next, we constructed an AND logic gate that leveraged the series (SERI) genetic architecture and two decoupled repressors (Fig. 5b). The AND gate was composed of the wild-type LacI repressor (I^+^_YQR_) and the non-natural transcription factor CelR adapted with the alternate DNA-binding domain TAN (E^+^_TAN_). In the absence of both effector ligands (IPTG and cellobiose) GFP was not produced, as both I^+^_YQR_ and E^+^_TAN_ repressed gene expression (i.e., blocked RNA polymerase function). The addition of IPTG or cellobiose alone cannot induce the full production of GFP, as at least one transcription factor (I^+^_YQR_ or E^+^_TAN_) remained in the repressed state. Only the addition of both effector ligands resulted in the full production of the reporter GFP, and led to the near digital performance of the engineered AND gate. In turn, we constructed a fully synthetic AND gate using two non-natural repressors I^+^_TAN_ and E^+^_YQR_ (Fig. 5d). Next, we constructed an OR gate via the parallel (PARA) genetic structure paired with two non-natural repressors E^+^_KSL_ and I^+^_TAN_ (Fig. 5c). Given the two channel architecture of this genetic circuit, there was an additive production of GFP (Supplementary Fig. 7c). This resulted in an inherently analog (opposed to digital) output performance^57^.

In digital devices that employ logic gates, there can only be two logic states, 1 and 0. However, digital devices are often driven by analog devices with an infinite range between a voltage high and a voltage low (ground). In order to convert an infinite number of states into only two outcomes, voltage logic levels are created by defining which voltage bands or ranges represent a logic high (1) or logic low (0)^58^. Likewise, we applied a similar set of threshold constraints to the OR logic gate (Supplementary Fig. 7c). We opted to set output values below 0.5 normalized output units (N.O.U.) to 0, and values above 0.5 N.O.U. to 1. Moreover, we applied the same logic state thresholds for 0 and 1 to all circuits described onward in this work. Analog performance is not unusual in biological circuits^57^, as illustrated in the OR gate we constructed in this study (Fig. 5c). To a lesser extent, analog performance was also observed in the relative outputs of the core and proximal DNA operator positions in the SERI architecture. For example, in our engineered AND gate (Fig. 5b) asymmetry was observed in the performance of each of the individually induced operators. Specifically, the relative performance of the E^+^_TAN_|O^tta^ (core) vs. I^+^_YQR_|O^sym^ (proximal) operations upon single ligand induction was not equivalent. The observed uneven off-states can be explained in part by the differences in performance of the engineered transcription factors (Supplementary Fig. 7b). In addition to transcription factor performance, Elowitz et al.^56^ illustrated performance asymmetry as a result of DNA operator position. Namely, the core position had stronger RNA polymerase interference (i.e., stronger repression) relative to the proximal position. Therefore, given equivalent transcription factor performance, the induction of a core genetic configuration resulted in lower gene production relative to the proximal configuration. Accordingly, a more digital AND gate was achieved via the incorporation of a more digital repressor-operator pair (R^+^_YQR_|O^sym^) at the proximal position (Fig. 5d and Supplementary Fig. 7d). Inspired by the asymmetry in performance of core and proximal operator configurations, we introduced two non-canonical logical half-AND operations HAND_(core)_ and hAND_(proximal)_ (Fig. 4c).

### Combinatorial logic gating via the series architecture

**Fig. 6:**
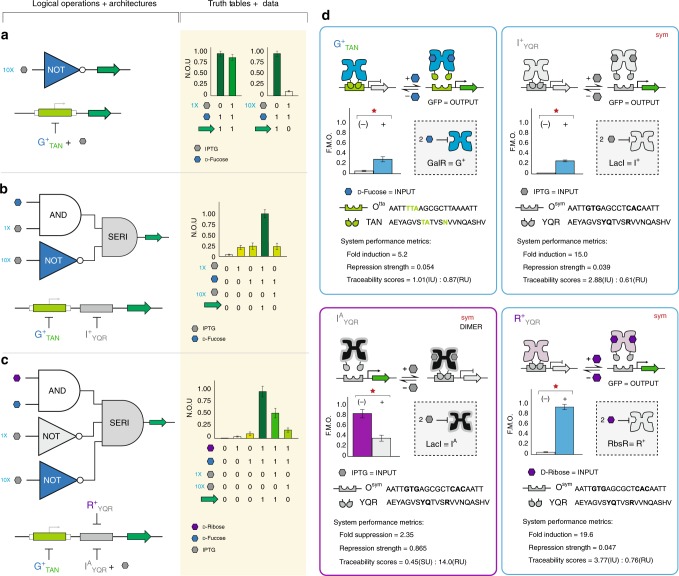
Combinatorial logic via the SERI architectures and bandpass operations. **a** IPTG acts as a competitive inhibitor to GalR, preventing GFP expression upon addition of fucose in the presence of 10 mM IPTG. When 1 mM IPTG is present, GalR is not inhibited. Values represent the mean of *n* = 6 biological replicates, and the error bars represent the 95% CI or ~2x s.e.m. **b** NOT logic function can be incorporated into a SERI circuit by utilizing IPTG as a competitive inhibitor to GalR, resulting in an apparent bandpass filter, where GFP output is achieved only in the presence of certain IPTG concentrations. Values correspond to *n* = 18 biological replicates. **c** A second NOT gate can be added by introducing an I^A^ transcription factor; this represents a more granular bandpass filter where the output can be gradually decreased upon addition of different concentrations of IPTG. Values correspond to *n* = 12 biological replicates except for the fucose, ribose, 1 mM IPTG state where *n* = 6 biological replicates. **d** Performance cards for each of the transcription factors involved. All data have been normalized to the maximum value of 20,000 GFP/OD_600_ a.u. Note: the I^A^_YQR_ suppressor used in **c** was reduced to a dimer (from a tetramer) to achieve repressor function. Source data provided as a Source Data file and individual data points overlaid as dot plots can be found in Supplementary Fig. 8

Combinatorial logic is a concept in which two or more fundamental operations (e.g., AND, OR, and NOT) can be systematically combined to produce more sophisticated logical gates. In principle, the combinational logic strategies that are employed toward the development of advanced electronic circuits can be applied to the fundamental biological edifice we developed in this study. Bearing in mind that electronic digital circuits are physically isolated, and gene circuits do not share the same degree of physical sequestration (unless they are cellularly compartmentalized^59^). Rather than viewing the lack of isolated operations within our systems as an impediment, we can leverage this inherent connectivity to generate unique combinations of logical operations (canonical or non-canonical) with programming rules that are distinctive to biological logic. Moreover, these deviations in biological circuit functions (from traditional digital circuit behavior) allow for the development of a vastly more dynamic programming language (i.e., circuits that can be simultaneously programmed horizontally and vertically). To demonstrate the potential of our emerging non-natural transcriptional programming structure, we constructed two combinatorial logic gates via the SERI genetic architecture. The first combinational program used two-signal coupled repressors, G^+^_TAN_ and I^+^_YQR_, and two cognate DNA operators O^tta^ (core) and O^sym^ (proximal) in series (Fig. 6a, b). The wild-type repressors LacI(I^+^) and GalR(G^+^) share a conditional signal overlap^41,45^. Studies have shown that 10 mM IPTG competitively inhibited the GalR repressor, and resulted an insensitivity to the cognate effector ligand d-fucose. Accordingly, we hypothesized that the engineered G^+^_TAN_ repressor would exhibit similar IPTG interference, and that this behavior could be used as a conditional repressor when coupled with I^+^_YQR_ in series. To test this hypothesis, we first demonstrated that 10 mM IPTG competitively inhibited the G^+^_TAN_ repressor, and resulted in insensitivity to the cognate effector d-fucose in isolation (Fig. 6a and Supplementary Fig. 8a). Objectively, this resulted in a conditional NOT gate, such that an apparent G^S^_TAN_ phenotype was observed in the presence of 10 mM IPTG (Fig. 6a). Next, we constructed a SERI genetic circuit that assumed an AND + NOT logical combination in which the AND gate is controlled by the addition of 1 mM IPTG and d-fucose to achieve a relative on-state (i.e., maximum GFP output). However, upon the addition of 10 mM IPTG and 10 mM d-fucose, the circuit reverts to the off-state (i.e., no GFP output). Practically, this combinatorial logic gate functions as an apparent bandpass filter that only allowed the production of the output interface (GFP) within a certain effector ligand concentration, and attenuated (rejected) GFP production at or above 10 mM IPTG (Fig. 6b and Supplementary Fig. 8b).

In our second iteration of a combinatorial logical gate via the SERI genetic architecture, we repurposed the O^tta^ (core) and O^sym^ (proximal) configuration, but deployed two decoupled non-natural repressors (R^+^_YQR_ and G^+^_TAN_) and one engineered antilac (I^A^_YQR_). In principle, the I^A^_YQR_ suppressor is coupled to the R^+^_YQR_ repressor via the DNA-binding function and is simultaneously coupled to the G^+^_TAN_ repressor via the conditional ligand interaction (as described above). When assembled, the resulting combinational logical program consists of a two-signal AND operation controlled via d-ribose and d-fucose, in series with two NOT gates controlled via IPTG (Fig. 6c). This three-signal logic gate functioned as a bandpass filter, similar to the previous example (Fig. 6b). However, the I^A^_YQR_ suppressor introduced a second but complementary NOT operation that was responsive to 1 mM IPTG. When the suppressor was activated, it reduced the maximum GFP output by approximately half (Supplementary Fig. 8c). The GFP output only achieved a full off-state when the I^A^_YQR_ suppressor was activated and the G^+^_TAN_ repressor was deactivated at 10 mM IPTG (i.e., the system rejected the d-ribose and d-fucose signal inputs). Thus, this three-signal program represented a more granular bandpass filter.

### Combinatorial Logic via parallel and series architectures

**Fig. 7:**
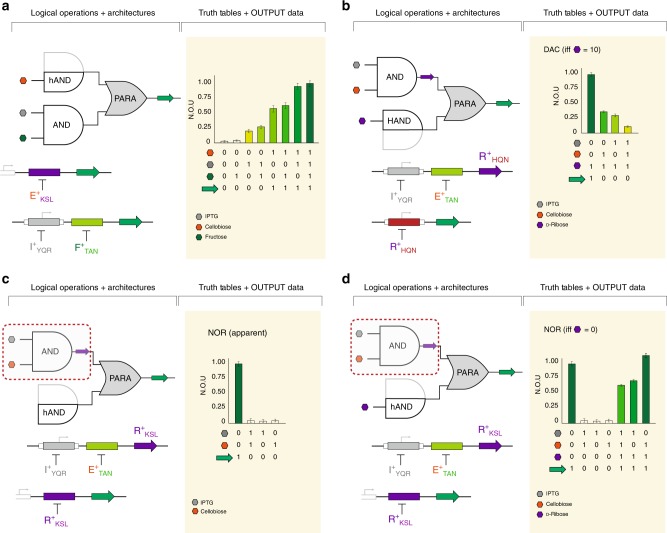
Digital-to-analog converters (DAC) and NOR gates. Analog behavior is inherent in biological systems and is exploited in the following circuits. **a** Asymmetric parallel configuration composed of three non-synonymous DNA operators. The progressive addition of ligands (which represent digital inputs 0 or 1) leads to a gradual increase in GFP output. **b** A second asymmetric parallel system (denoted AND [PARA] HAND) where the two channels are coupled via an engineered TF-operator pair. The production of R^+^_HQN_ is dependent on the addition of IPTG and cellobiose such that progressive addition of inputs leads to a decrease in GFP output. 10 mM ribose is required to alleviate repression. **c** Slight modifications to the previous circuit generate vastly different performance. In this iteration, R^+^_KSL_ (interacting with a proximal O^acc^) replaces R^+^_HQN_ (interacting with a core O^tgg^) and the change in performance characteristics leads to a more digital output profile, giving the appearance of a NOR gate. Red dashed box indicates a portion of the circuit that is influenced by the performance of the engineered transcription factor (i.e. the SERI operation behaves more like an OR rather than AND). **d** Upon addition of d-ribose, the NOR gate is deactivated such that the program becomes NOR if and only if (iff) there is no d-ribose present. Source data provided as a Source Data File and individual data points overlaid as dot plots can be found in Supplementary Fig. 9

In addition to our ability to leverage the series operator configuration to achieve combinatorial logic gating, we expanded our genetic programming capability via the incorporation of parallel operator architectures. For example, we constructed an asymmetric parallel (PARA) configuration composed of three non-synonymous DNA operators (Fig. 7a). The top channel consisted of a single non-natural DNA operator (O^agg^) located in the proximal position. Whereas, the bottom channel had a SERI architecture and included O^sym^ (core) and one non-natural operator O^tta^ (proximal). The genetic structure was complemented by three decoupled transcription factors I^+^_YQR_, E^+^_KSL_, and F^+^_TAN_, that were responsive to three disparate effector ligands (IPTG, cellobiose, and fructose – respectively). Both channels returned the same GFP output interface. Objectively, the system resulted in a non-canonical logical program (hAND [PARA] AND) controlled via three-signal inputs. This genetic program had a performance profile comparable to a digital-to-analog converter (DAC), where the system generated various output levels that corresponded to different digital input combinations (Supplementary Fig. 9a).

Our next iteration of a non-canonical combinatorial logic gate that functioned as a digital-to-analog converter was objectively represented as AND [PARA] HAND (Fig. 7b and Supplementary Fig. 9b). However, instead of two synonymous outputs, we coupled the top channel to the bottom channel via a non-natural transcription factor paired to a DNA operator located on the second channel that controlled the GFP output. The production of the R^+^_HQN_ repressor was controlled via a two-signal AND gate. When the signal IPTG or cellobiose was added the top channel produced intermediate amounts of the engineered repressor. Whereas, the maximum R^+^_HQN_ repressor output was only produced upon the addition of both signal inputs at 10 mM. The production of the R^+^_HQN_ transcription factor was coupled to a GFP output interface, located on the bottom channel. The R^+^_HQN_ repressor was paired with the cognate operator O^ttg^, with a background of 10 mM d-ribose under every condition to alleviate repression. This DAC program presented an inverted series of outputs, relative to the previous DAC program (Fig. 7a, b). Thus, the absence of IPTG and cellobiose resulted in maximum GFP output. Output attenuation was only achieved once different combinations of IPTG or cellobiose are introduced, with GFP maximally repressed when both IPTG and cellobiose were present (Fig. 7b). Notably, this digital-to-analog converter generated the presented output if and only if (iff) d-ribose was present at 10 mM (Supplementary Fig. 10). Moreover, the DAC output profile was de-amplified or muted via the addition of 5 mM or 0 mM d-ribose, respectively.

As mentioned in previous sections, the performance of a given transcriptional program is dictated by the relative performance metrics of the system of transcription factors and DNA operator architectures selected to construct the final structure. To demonstrate how the performance of an engineered repressor influenced the apparent outcome of genetic programs (with similar structure), we modified the single GFP output digital-to-analog converter developed in the last iteration (Fig. 7b). The objective representation of the modified system was AND [PARA] hAND (Fig. 7c). The key differences between the two programs being: (i) the exchange of R^+^_HQN_ for R^+^_KSL_, (ii) the relative position of the operator being acted on was moved from the core to the proximal position, (iii) the R^+^_KSL_ repressor had a fold induction that was less than one third of that observed for R^+^_HQN_. Given the significant change in performance of R^+^_KSL_, relative to R^+^_HQN_ (Supplementary Fig. 9c), the observed performance switches from analog outputs (that vary with different combinations of input signals) to a more digital output profile. Moreover, reassessment of the truth table and corresponding output data revealed the development of an apparent NOR gate (Fig. 7c). However, if we introduced the cognate effector ligand to R^+^_KSL_ (d-ribose) the NOR program was deactivated (Fig. 7d and Supplementary Fig. 9d). Thus, this program functioned as a NOR gate, if and only if (iff) d-ribose = 0.

### Transcriptional programming via the master architecture

**Fig. 8:**
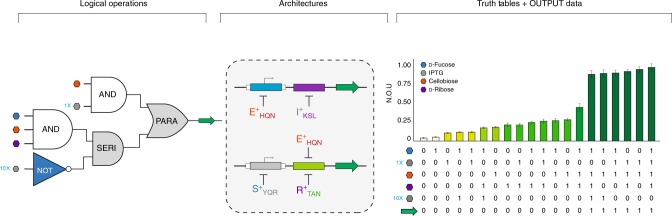
A complex program via the master architecture. Arranging two SERI structures in a PARA configuration leads to the development of the genetic master circuit. This configuration employs four non-synonymous DNA operators and allows for the generation of any of the logical programs presented in this study. When both outputs are the same (GFP), this gives rise to the most granular digital-to-analog converter in this study. The top channel contains two operators arranged in a SERI architecture and therefore functions as a digital-like AND gate with respect to cellobiose and IPTG. By capitalizing on off-diagonal interactions, the bottom channel becomes a three-signal AND gate with respect to the ligands d-fucose, d-ribose, and cellobiose. Finally, we take advantage of the ligand overlap between GalS and LacI to generate additional output states with varying GFP outputs. Values represent the mean of *n* = 12 biological replicates and error bars indicate the 95% CI or ~2x s.e.m. *Y*-axis is given as normalized output units or N.O.U. This is calculated by taking GFP fluorescence (485ex., 510em.) normalized to OD_600_, which is further normalized to the maximum output for each experiment. Source data provided as a Source Data File and individual data points overlaid as dot plots can be found in Supplementary Fig. 11

Finally, we constructed a master architecture in which two series genetic structures are positioned in parallel (SERI [PARA] SERI). In principle, the genetic master circuit can generate any of the logical programs presented in this study, provided the appropriate systems of engineered transcription factors and cognate operators are selected. Using the master architecture we constructed a full four-signal combinatorial program (Fig. 8 and Supplementary Fig. 11a). The performance of this system (with two synonymous output reporters) gave rise to our most granular digital-to-analog converter. In addition to the DAC performance, the program had a time-independent step response (output) profile. The observed input dependent step response was the result of an imposed asymmetric output maxima of the top and bottom channels (Supplementary Fig. 11a). Namely, the bottom channel has a maximum output (inputs = d-fucose + cellobiose + d-ribose) that was less than half that of the top channel (inputs = IPTG + cellobiose). Resulting output signals are non-discrete, and convoluted by operator coupling between O^ctt^ (top core) and O^tta^ (bottom proximal) facilitated *via* non-natural repressor E^+^_HQN_. In addition to operator coupling, this program was IPTG signal coupled *via* S^+^_YQR_ and I^+^_KSL_, where 10 mM IPTG inhibits the GalS transcription factor (S^+^_YQR_). Program complexity notwithstanding, the fidelity of this system was remarkably high, implying that a priori (in silico) forecasting may be possible. Additional iterations of this master architecture paired with different non-natural transcription factors resulted in vastly different outcomes (Supplementary Fig. 11b, c). Moreover, the impact of multiple transcription factors had an impact on the microbe chassis growth rate (Supplementary Fig. 12 and Supplementary Note 3). In most cases, there was no appreciable difference between the growth curves of cells coding for 0–2 transcription factors. However, once the cells expressed either 3 or 4 transcription factors, a marked decrease in the growth rate was observed as evidenced by the doubling time, or the slope of the exponential region of the growth curve. In some cases, specific combinations of transcription factors impacted the growth rate. Interestingly, all cells reached the exponential phase at approximately the same time and quickly diverged in their growth profiles. While, multiple transcription factors expressed in the chassis exerted a sizable burden on the cells; the cells were still capable of robust, predictable, and reproducible circuit performance.

## Discussion

In this study we introduce a biological programming edifice based on an engineered system of non-natural transcription factors and complementary genetic architectures. The SERI and PARA structures were used to systematically arrange cognate DNA operators and facilitate the development of combinatorial logic gating. Given that the engineered transcription factors are adapted with alternate DNA recognition and operator DNA elements that are not represented in nature, the resulting logical operations can be constructed and operated alongside existing natural genetic programs. Moreover, we have adapted each representative regulatory core domain with the native LacI DNA-binding domain. This will facilitate program coupling with the wild-type LacI repressor (I^+^_YQR_) or engineered antilac suppressor (I^A^_YQR_), via a shared DNA operator O^1^ (or O^sym^). The six alternate DNA recognition (ADR) domains used in this study to confer repressive function in E^+^, G^+^, S^+^, F^+^ and R^+^ RCDs, are also shared among 46 engineered antilacs (I^A^_ADR_) and 6 related I^+^_ADR_ repressors. This will enable a broad range of functional coupling by way of shared DNA-binding functions. The coupling of engineered transcription factors can also be extended via shared ligand binding functions. Namely, seven I^+^_ADR_ repressors, seven I^A^_ADR_ suppressors, seven G^+^_ADR_ and six S^+^_ADR_ repressors share a common binding function to IPTG – though disparate phenotypes upon effector ligand binding. While this system of engineered transcription factors is large (i.e., 27 × + _ADR_ – where X = E, G, S, F and R; 46 I^A^_ADR_; and 6 I^+^_ADR_), the putative combinatorial design space that can be used toward the development of additional non-natural transcription factors is even more astonishing. Swint-Kruse et al.^43^ estimates that there are more than 1000 regulatory core domains that share the same topology to those used in this study. In addition, Lewis et al.^55^ has identified ~200 non-natural (alternate) DNA-binding domains and cognate operator combinations that can potentially be adapted to any given RCD. Consequently, these parts represent a combinatorial design space of ~10^5^ putative transcription factors. Even if only 1% of these engineered transcription factors are functional, this could lead to the generation of hundreds of non-natural regulatory proteins with a variety of performance characteristics.

Ultimately, we are interested in codifying our programming structure and the first step toward this goal is to establish a metrology for our system of engineered transcription factors. This metrology will ensure that we can confidently compare the performance metrics of a given transcription factor between laboratories, which sets the stage for predicting performance of a bespoke transcriptional program prior to construction. In brief, our metrology consists of three parts: (i) defining the conditional units of measurement for a given transcription factor, (ii) reproducible realization of these units at steady-state, (iii) performance traceability via the comparison of the performance metrics of a given transcription factor to a reference system. We begin this metrology via the development of performance cards for each of the transcription factors developed or used in this study (Supplementary Fig. 6 and Supplementary Note. 2). On the front of the card we display the complete engineered system and the putative phenotypic mechanism. In addition, the front of the card summarizes the contextual performance metrics for each system (i.e., normalized repression strength and fold induction), plus the relative performance to a reference standard (i.e., I^+^_YQR_ paired with the DNA operator O^1^; ±10 mM IPTG) to establish traceability. The back of a given card displays the operator position, microbe chassis, relevant genetic elements and related information. Collectively, this represents a standardized set of 55 unit operations. Recent studies^60,61^ have demonstrated the impact that genetic architecture can have on the regulatory properties of a given transcription factor, justifying the need for our standardization approach (which will aid in both benchmarking and reproducibility). While we found the dynamic range of the unit operations useful in this study, the proposed metrology will enable other end-users to systematically tune the performance of a given transcription factor system to meet individual needs. The estimated scale-out of our current programming edifice is represented by approximately 86,184 putative biomolecular systems for the master architecture, when complemented by our current set of engineered transcription factors (Supplementary Note. 4, Supplementary Software 1, Supplementary Table 1, and Supplementary Fig. 13). This estimate can be expanded further if we consider the practical possibility that multiple transcription factors can engage a single DNA operator (Figs. 6c and 8). This study represents an important advance in synthetic biology via expanding biological computing capacity, and lays the foundation for the development of a nascent biological (non-natural) programming language.

## Methods

### Vector construction and reporter systems

All chimeras were inserted into the pLacI plasmid (Novagen), which features a low copy number p15A origin, a chloramphenicol resistance marker, and the gene for the repressor regulated with a constitutive LacI promoter. The different RCDs were obtained as follows: GalS (Addgene #60773), FruR (Addgene #60768), RbsR (Addgene #60773) from the Swint-Kruse and Bennett Labs^45^, while GalR and CelR were gifts from the Collins lab^41^. The open reading frame for each respective gene was amplified via PCR and inserted into the pLacI vector using circular polymerase extension cloning (CPEC). Mutations to the DNA-binding domain were then introduced via routine site-directed mutagenesis using Phusion DNA polymerase with GC buffer (New England Biolabs). For the FruR variants, the traditional lacI promoter was replaced by the stronger lacIq promoter^62^, which leads to a tenfold increase in protein production. This single nucleotide change was introduced via NEB Q5 Site Directed Mutagenesis (New England Biolabs) using the NEBaseChanger tool to design primers. For each repressor variant, the entire gene reading frame along with the promoter driving expression was sequenced in the forward and reverse direction (Eurofins Genomics) to verify all mutations and correct assembly. A reporter plasmid system was constructed starting with the pZS*22-sfGFP reported in Richards et al.^54^. This plasmid features a low copy number pSC101* origin of replication, and a kanamycin resistance marker. The region of the plasmid excluding the promoter and operator was PCR amplified, visualized on an Agarose Gel, and Gel Extracted (Qiagen). A small fragment containing the constitutive *trc* promoter (hybrid of trp and lacUV5 promoters), a 5-bp spacer segment, and the operator sequence was constructed via oligos (Eurofins Genomics) and placed into the pZS*22-sfGFP vector through CPEC. This is the same reporter system utilized by Rondon and Wilson^53^. For each operator variant, the region upstream of the reporter gene, along with the GFP reading frame was sequenced in the forward and reverse direction (Eurofins Genomics) to verify correct assembly. Detailed sequence data (i.e., promoter sequences, transcription factors, RCD, ADR and operator pair information and vector maps are given in Supplementary Figs. 1, 2, and 4. Relevant primers are given in Supplementary Data 1.

### Construction of operators in series and parallel

The trc promoter was used as a scaffold, but the 18 bp region between the −35 (TTGACA) and −10 (TATAAT) hexamers was replaced with 18 bp of an operator sequence (akin to the original pLacO1 architecture^12^) — in the core^56^ position. The second operator was then introduced in the proximal^13^ position, 15 bp downstream of the end of the −10 hexamer (Supplementary Fig. 4). In order to prevent variations in gene output stemming from operator sequence identity, we employed and insulator part, specifically the self-cleaving ribozyme RiboJ, which has been shown to be an effective buffer against transfer-function variability^63^. RiboJ was inserted between the proximal promoter and the ribosome binding site to eliminate transfer-function variability due to the 5′ untranslated region (UTR). To build the parallel circuits, the GFP gene along with the rrnB T1 terminator was PCR amplified from the pZS*22-sfGFP plasmid, visualized on an agarose gel, and gel extracted (Qiagen). On this second copy of GFP, we used the strong pL promoter^64^ as the scaffold and replaced the 18 bp region between the −35 (TTGACA) and −10 (GATACT) hexamers with 18 bp of an operator sequence. The second operator was then introduced in the proximal position, 15 bp downstream of the end of the −10 hexamer. Like the original reporter, we employed and insulator part, specifically the self-cleaving ribozyme RiboJ to prevent transfer-function variability stemming from differences in operator sequence. In order to avoid evolutionary instability due to homologous recombination, an engineered variant, RiboJ10^50^ was used upstream of GFP to introduce sequence diversity, while maintaining function. This region (the promoter, operator, RiboJ and the ribosome binding site) was constructed via oligos and the three fragments were then combined via splicing by overlap extension (SOE)^65^ and introduced into the linearized form of the reporter plasmid described previously via CPEC. For each construct, the region upstream of the reporter gene (containing the promoter, operator, and insulator), along with the GFP reading frame was sequenced in the forward and reverse direction (Eurofins Genomics) to verify correct assembly.

### Transcription factor vehicles

To introduce additional transcription factors (TF), a third plasmid bearing another LacI/GalR family chimera was created. A plasmid with an alternate selection marker and compatible origin of replication was needed and to this end, the AmpR coding region of pLS1 was PCR amplified, visualized on an agarose gel, and gel extracted (Omega). This was then combined with the X^+^ coding region from pLacI via Splicing by Overlap Extension (SOE). Finally, the PBR322 origin of replication was PCR amplified from the pet28b vector (a gift from the Kane lab), visualized on an agarose gel, and gel extracted (Qiagen) and combined with the X^+^ and AmpR coding regions via CPEC. This plasmid also uses the strong lacIq promoter to drive expression of the transcription factor and was then co-transformed along with the pLacI plasmid and the reporter plasmid for assaying. In the case that more than two TFs were needed for a Transcription Factor system, plasmids containing more than one open reading frame (ORF) needed to be constructed. First, a linearized form of the pLacI plasmid was created by amplifying a 3764-bp region of the plasmid, visualized on an agarose gel, and gel extracted (Qiagen) the fragment. Next, the ORF for another X^+^ variant was PCR amplified from pSO and dpnI digested in order to remove any background template. The two were then combined using the NEBuilder HiFi kit (New England Biolabs). Similarly, a 3648-bp region of pSO was amplified, visualized on an agarose gel, and gel extracted (Qiagen) and combined with the ORF of a given X + from pLacI. The latter was dpnI digested and the two fragments were combined once again using the NEBuilder HiFi kit. This allowed for the introduction of up to four TFs to be simultaneously transformed along with the reporter plasmid into *E. coli*. For these plasmids, each Transcription Factor was sequenced independently along with their respective promoters to ensure proper assembly. Vector maps are given in Supplementary Fig. 4b. Relevant primers are given in Supplementary Data 1.

### Microplate assay for transcription phenotyping

All experiments were performed in the cell strain 3.32^66^ (Genotype *lacZ13(Oc), lacI22, LAM–, el4–, relA1, spoT1, and thiE1*, Yale CGSC #5237), which is an *E. coli* K12 strain that has LacI and the lac operon deleted. The plasmids were co-transformed and plated on LB agar with the appropriate antibiotics (chloramphenicol for pLacI, ampicillin for pSO, and kanamycin for the reporter plasmid). Microplate assays were performed as outlined by Richards et al.^54^. Briefly, colonies were inoculated in 1 mL of LB and grown overnight at 37 °C shaking at 300 rpm. After this initial growth period, cultures were diluted into 200uL wells in M9 minimal media supplemented with 0.2% casamino acids, 1 mM Thiamine HCl and the appropriate antibiotics containing the appropriate effector ligand (all ligands in this work were used at a final concentration of 10 mM, unless otherwise stated). Ligands used are as follows: IPTG (CAS 367–93–1, Thermo Fischer), d-Fucose (CAS 3615–37–0, Carbosynth), Cellobiose (CAS 528–50–7, Arcos Organics), d-Ribose (CAS 50–69–1 Arcos Organics), d-Fructose (CAS 57–48–7 Alfa Aesar). Each sample was aliquoted in six samples in a clear, sterile, conical-bottom 96-well plate (Fischer Scientific) and grown in a 37 °C shaker at 300 rpm covered with a Breathe-Easier sealing membrane (Midwest Scientific) to prevent evaporation. After 20 h, all wells were transferred to a black 96-well plate (COSTAR) for assaying and GFP fluorescence (ex. 485 nm, em. 510 nm, gain 50) and optical density (OD600) were measured using a Synergy HT plate reader (BioTek). Corrections for pathlength were made using OD900 and OD975 and the fluorescence values were normalized to the optical density and averaged across all biological replicates. For each operator variant, the maximum GFP expression was determined using the LacStop control plasmid. LacStop is a plasmid that contains the LacI coding sequence on the pLacI plasmid but includes a stop codon at positions 2 and 3 (Supplementary Fig. 2). Therefore, this plasmid produces no repressor while still exerting the metabolic load of a second plasmid; this LacStop control was used to determine the maximum expression that can be achieved by a given architecture and can therefore be used for normalization, see Supplementary Fig. 4.

### Statistical analysis for phenotype classification

Phenotypes were determined by first comparing the mean GFP (fluorescent) output for *n* = 12 biological replicates six trials each on two different days) in the presence and absence of inducer utilizing a student’s t-test with unequal variances and allowing for unequal sample sizes. The significance level was set to 0.001 and a one-tailed test was used. The rationale for a one-tailed rather than two tailed test is that the ligands utilized in this study have been previously shown to be inducers of their respective RCD and therefore, we were strictly concerned with cases in which the mean fluorescence was higher in the presence of inducer than in the absence. For a full table of descriptive statistics, including effect size (Cohen’s d values) see Supplementary Data 2. Variants for which there was no statistical difference upon addition of induction were classified as either X^−^ or X^s^ depending on the magnitude of the fluorescence output. Variants with a GFP expression of less than 50% of LacStop under the same conditions (same operator and same ligand) were classified as X^s^ or super-repressor phenotypes while a variant with GFP expression greater than 50% of LacStop was classified as an X^−^ phenotype.

### Reporting summary

Further information on research design is available in the Nature Research Reporting Summary linked to this article.

## Supplementary information

Supplementary Information

Description of Additional Supplementary Files

Supplementary Software 1

Supplementary Data 1

Supplementary Data 2

Reporting Summary

Source Data

## Data Availability

The authors declare that all analyzed data supporting the findings of this study are available within the paper [and its supplementary information files]. The source data underlying Figs. 2, 3, 5, 6, 7, and 8 are provided as a Source Data file. The data supporting the findings of this study are available from the corresponding author upon reasonable request. The sequences of the following plasmids are provided in GenBank: CelR Variants (Accession #s MN207910 - MN207915), FruR Variants (MN207916 - MN207921), GalR Variants (MN207922 - MN207928), GalS Variants (MN207929 - MN207935), RbsR Variants (MN207958 - MN207963) Antilac Variants (MN207936 - MN207944), Master Architecture (MN207945), RbsR Reporters (MN207946, MN207947), pSO plasmid variants (MN207948 - MN207951), pSOx2 plasmids (MN207952 - MN207954), pLac-Lac plasmids (MN207955 - MN207957), Single Reporter Variants (MN207964 - MN207971), and Series Reporter (MN207972).

## Code availability

All custom code (Supplementary Software 1) is available at: https://github.com/AndrewEShort/Wilson-Lab.
