# Supplementary material for: Transcriptional programming using engineered systems of transcription factors and genetic architectures
Source: Nat Commun. 2019 Oct 21;10:4784. doi: 10.1038/s41467-019-12706-4 (PMC6803630; doi:10.1038/s41467-019-12706-4)
Supplement: Supplementary file 1 — Supplementary Information [file 41467_2019_12706_MOESM1_ESM.pdf]

SUPPLEMENTARY INFORMATION:

Transcriptional programming using engineered systems of transcription factors and genetic architectures

Ronald et al.

Supplementary Note 1: All five regulatory core domains functioned as repressors (X^+) when adapted with YQR, HQN, or TAN ADR-domains and paired to cognate operator DNA elements O^1, O^{ttg}, and O^{tta} (respectively), see main text **Fig. 2a-e**. ADR-domains GKR and KSL conferred the second most successful set of engineered systems, such that 4 out of 5 functioned as repressors (X^+). ADR-domain GKR was functional when paired with the regulatory cores of CelR, GalR, RbsR, and GalS, and resulted in non-natural repressors E^+_{GKR}, G^+_{GKR}, R^+_{GKR}, and S^+_{GKR}. The KSL DNA binding domain was functional only when paired with CelR, GalR, FruR, and RbsR, which resulted in repressors E^+_{KSL}, G^+_{KSL}, F^+_{KSL}, and R^+_{KSL}. Only three regulatory core domains paired with HTK and resulted in three chimeras that functioned as repressors (*i.e.*, G^+_{HTK}, R^+_{HTK}, and S^+_{HTK}). NAR was the least successful alternate DNA recognition domain, and resulted in one functional system (repressor G^+_{NAR}). Interestingly, operator element O^{gta} (*i.e.*, the cognate operator to NAR) has been previously referred to as the "gal-like operator" by Daber and Lewis[1]. Likewise, only GalS (S^S_{NAR}) and GalR (G^+_{NAR}) NAR-chimera bound to O^{gta}, see main text **Fig. 2**. The GalR (G^+_{ADR}) regulatory core domain was the most amenable to modular design (*i.e.*, all 7 ADR-domains conferred repressive function), see main text **Fig. 2b**. The ribose repressor (R^+_{ADR}) was the second most successful modular design, and resulted in repressor functions when paired with 6 out of 7 ADR, main text **Fig. 2c**. CelR and GalS paired with 5 out of 7 ADR and resulted in the repressor phenotype in each case, main text **Fig. 2a,d**. Finally, FruR was the least amenable to ADR adaptation, with only 4 out of 7 repressive chimeras reported, **Fig. 2e**. A detailed description of individual engineered transcription factor performance is given in **Supplementary Figs. 3 and 6**.

In the X^+_{YQR} repressor suit, $S^+_{YQR}|O^1$ performed nearly *on par* to the reference system. Whereas, $R^+_{YQR}|O^1$ had a fold-induction more than 3-times that of the I^+_{YQR} $|O^1$ reference system. Engineered transcription factors I^+_{HQN}, E^+_{HQN}, and R^+_{HQN} had induction values greater than the reference state (*i.e.*, 1.3x, 4.5x and 2.9x, respectively) when paired with the cognate non-natural operator O^{ttg}. $G^+_{TAN}|O^{tta}$ was *on par* with $I^+_{YQR}|O^1$ while E^+_{TAN} had a fold induction 1.7 times that of the LacI reference system. I^+_{GKR} and E^+_{GKR} had relative fold induction values of 1.3x and 2.0x, respectively, when paired with the O^{gac} operator. I^+_{KSL}, E^+_{KSL}, and F^+_{KSL} (paired with O^{agg}) were 1.9x, 5.2x, and ~1x, relative to $I^+_{YQR}|O^1$ performance metrics. Finally, all X^+_{HTK} and X^+_{NAR} variants had induction properties that are less than the reference system. The E^+ regulatory core domain with alternate DNA binding resulted in the greatest number of transcription factors with induction values that exceeded the $I^+_{YQR}|O^1$ reference system at a total of four engineered systems (*i.e.*, E^+_{HQN}, E^+_{TAN}, E^+_{GKR}, and E^+_{KSL} – paired with cognate operators O^{ttg},O^{tta},O^{gac}, and O^{agg}, respectively). Three super-repressors were observed that interacted with cognate operator DNA but were unresponsive to effector ligands. 22 out of the 27 engineered transcription factors interacted and functioned orthogonally with their cognate operator DNA — *i.e.*, repression and induction only with the intended operator DNA element. We observed 7 functional cross-interactions in which a given engineered transcription factor interacted with a non-cognate DNA operator and was inducible by the appropriate ligand, see **Fig. 2**. The X^+_{HQN} variants had the greatest number of cross-interactions (*i.e.*, $E^+_{HQN}|O^{tta}$, $E^+_{HQN}|O^{ctt}$, $E^+_{HQN}|O^1$, and $R^+_{HQN}|O^{tta}$), see main text **Fig. 2** and **Supplementary Fig. 6**. The HTK adapted variants resulted in two transcription factors S^+_{HTK} and E^+_{HTK} that interacted (and were functional) with non-cognate operators O^{agg} and O^{tta}, respectively. Finally, R^+_{GKR} is cross-functional with O^1. In addition to cross-interactions that resulted in repressive phenotypes, one super-repressor was observed F^S_{HQN} that binds to non-cognate operator O^{ctt}.

Supplementary Figure 1

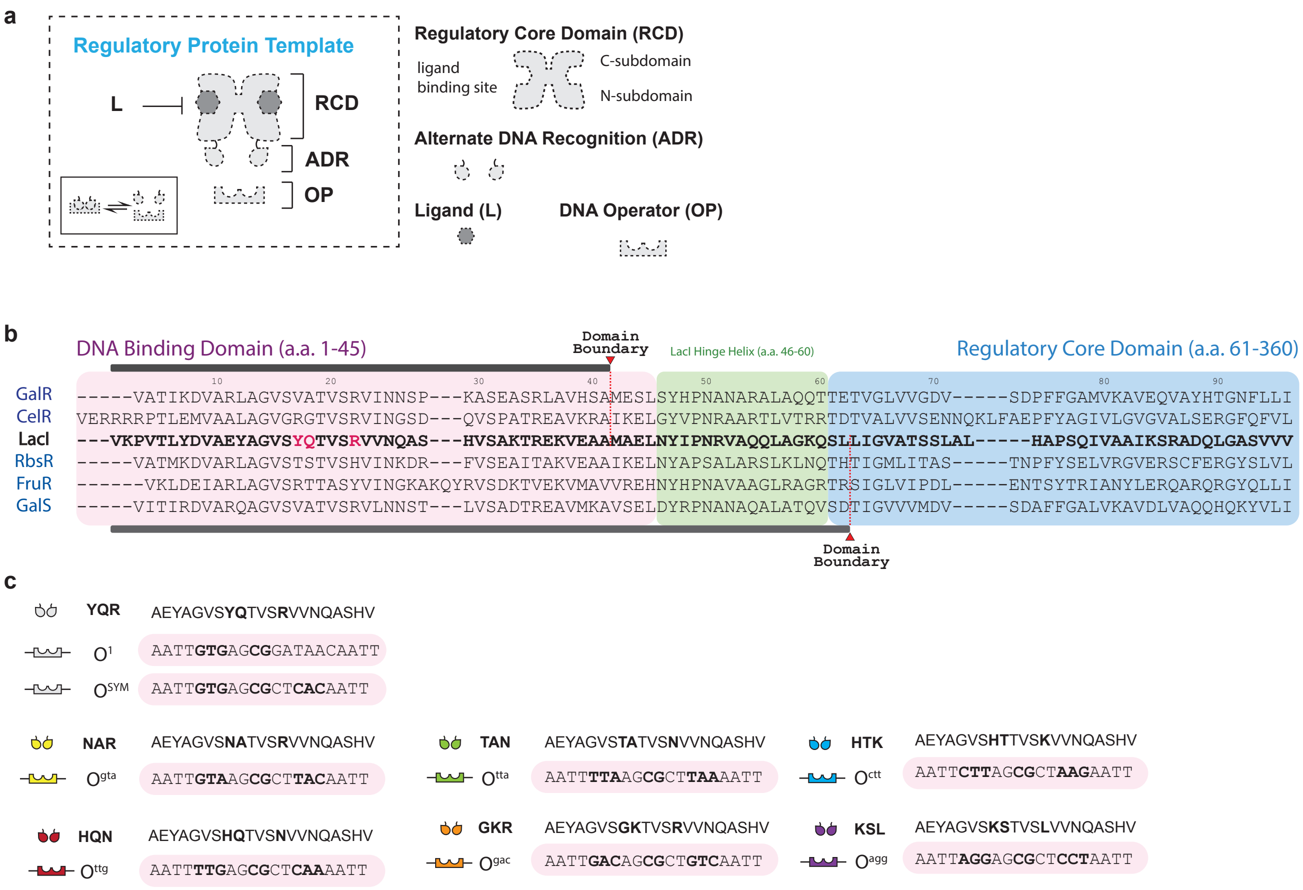

Supplementary Figure 1: LacI/GalR RCD sequence alignment and ADR/OP pairs. **(a)** Regulatory Protein Template as seen and described in **Fig. 1.** **(b)** The LacI/GalR homologs used in this study were subjected to a Multiple Sequence Alignment (MSA) utilizing the Clustal Omega program from EMBL-EBI. The sequence for LacI is shown in bold and the residues are numbered along the top of the alignment. GalR and CelR were obtained as a gift from the Collins lab and the module boundary between the ADR unit and the RCD occurs at amino acid 42, while RbsR, FruR and GalS were obtained via Addgene and the module boundary occurs at amino acid 62. Residues 46-60 encompass the LacI Hinge Helix and are shown with a green background. LacI Residues 17, 18 and 22 purportedly are responsible for DNA recognition and as such, are shown in red. Sequence identity of each homolog relative to LacI was calculated and the results are as follows: GalR & LacI – 26% identical, CelR & LacI – 29% identical, RbsR & LacI – 31% identical, FruR & LacI – 22% identical, GalS & LacI – 28% identical. **(c)** Full sequences for each of the DNA operators (inside pink ovals) and the amino acid sequence for the corresponding ADR (amino acids 10-30 shown with positions 17,18, and 22 in bold). Each ADR is grouped along with its cognate DNA Operator (OP).

Supplementary Figure 2

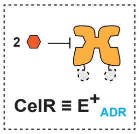

VKPVTLYDVAEYAGVSXXTVSXVVNQASHVSAKT
REKVEAAIKELGYVPNRAARTLVTRRTDTVALVVS
ENNQKLFAEPFYAGIVLGVGVALSERGFQFVLAT
GRSGIEHERLGGYLAGQHVDGVLLLSLHRDDPLP
QMLDEAGVPYVYGGRPLGVPEEQVSYVDIDNIG
GGRQATQRLIETGHRRIATIAGPQDMVAGVERLQ
GYREALLAAGMEYDETLVSYGDFTYDSGVAAMR
ELLDRAPDVDAVFAASDLMGLAALRVLRASGRRV
PEDVAVVGYDDSTVAEHAEPPMTSVNQPTELMG
REMARLLVDRITGETTEPVRLVLETHLMVRESG

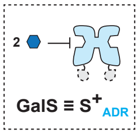

VKPVTLYDVAEYAGVSXXTVSXVVNQASHVSAKT
REKVEAAMAELNYIPNRVAAQLAGKQSDTIGVVV
MDVSDAFFGALVKAVDLVAQQHQKYVLIGNSYHE
AEKERHAIEVLIRQRCNALIVHSKALSDDELAQFM
DNIPGMVLINRVVPGYAHRCVCLDNLSGARMATR
MLLNNGHQRIGYLSSSHGIEDDAMRKAGWMSAL
KEQDIIPPESWIGAGTPDMPGGEAAMVELLGRNL
QLTAVFAYNDNMAAGALTALKDNGIAIPLHLSIIGF
DDIPIARYTDPQLTTVRYPIASMAKLATELALQGAA
GNIDPRASHCFMPTLVRRHSVATRQNAAAITNST
NQAM

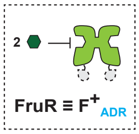

VKPVTLYDVAEYAGVSXXTVSXVVNQASHVSAKT
REKVEAAMAELNYIPNRVAQQLAGKSSRSIGLVIP
DLENTSYTRIANYLERQARQRGYQLLIACSEDQP
DNEMRCIEHLLQRQVDAIIVSTSLPPEHPFYQRW
ANDPFPIVALDRALDREHFTSVVGADQDDAEML
AEELRKFPAETVLYLGALPELSVSFLREQGFRTA
WKDDPREVHFLYANSYEREAAAQLFEKWLETHP
MPQALFTTSFALLQGVMDVTLRRDGKLPSDLAIA
TFGDNELLDFLQCPVLAVAQRHRDVAERVLEIVL
ASLDEPRKPKPGLTRIKRNLYRRGVLSRS-

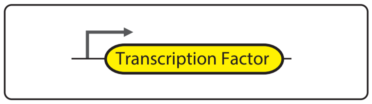

5′-GACACCATCGAATGGCGCAAAACCTTTCGCGGTATGGCATGATA
GCGCCCGGAAGAGAGTCAATTCAGGGTGGTGAATGTG- 3′
(start codon)

lacI Promoter

GACACCATCGAATGG**C**GCAAAACCTTTCGCGGTATGGCATGATAGCGCCCGGAAGAGAGTCAATTCAGGGTGGTGAAT
-35 -10

lacIq Promoter

GACACCATCGAATGG**T**GCAAAACCTTTCGCGGTATGGCATGATAGCGCCCGGAAGAGAGTCAATTCAGGGTGGTGAAT
-35 -10

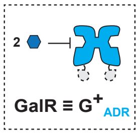

VKPVTLYDVAEYAGVSXXTVSXVVNQASHVSAKTR
EKVEAAMESLSYHPNANARALAQQTTETVGLVVG
DVSDPFFGAMVKAVEQVAYHTGNFLLIGNGYHNE
QKERQAIEQLIRHRCAALVVHAKMIPDADLASLMK
QMPGMVLINRILPGFENRCIALDDRYGAWLATRHLI
QQGHTRIGYLCSNHSISDAEDRLQGYYDALAESGI
AANDRLVTFGEPDESGGEQAMTELLGRGRNFTAV
ACYNDSMAAGAMGVLNDNGIDVPGEISLIGFDDVL
VSRYVRPRLTTVRYPIVTMATQAAELALALADNRP
LPEITNVFSPTLVRRHSVSTPSLEASHHATSD

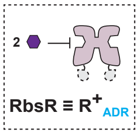

VKPVTLYDVAEYAGVSXXTVSXVVNQASHVSAKT
REKVEAAMAELNYIPNRVAQQLAGKASHTIGMLIT
ASTNPFYSELVRGVERSCFERGYSLVLCNTEGDE
QRMNRNLETLMQKRVDGLLLLCTETHQPSREIMQ
RYPTVPTVMMDWAPFDGDSDLIQDNSLLGGDLAT
QYLIDKGHTRIACITGPLDKTPARLRLEGYRAAMK
RAGLNIPDGYEVTGDFEFNGGFDAMRQLLSHPLR
PQAVFTGNDAMAVGVYQALYQAELQVPQDIAVIG
YDDIELASFMTPPLTTIHQPKDELGELAIDVLIHRIT
QPTLQQQRLQLTPILMERGSA

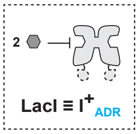

VKPVTLYDVAEYAGVSXXTVSXVVNQASHVSAKT
REKVEAAMAELNYIPNRVAQQLAGKQSLLIGVATS
SLALHAPSQIVAAIKSRADQLGASVVVSMVERSG
VEACKTAVHNLLAQRVSGLIINYPLDDQDAIAVEAA
CTNVPALFLDVSDQTPINSIIFSHEDGTRLGVEHLV
ALGHQQIALLAGPLSSVSARLRLAGWHKYLTRNQ
IQPIAEREGDWSAMSGFQQTMQMLNEGIVPTAML
VANDQMALGAMRAITESGLRVGADISVVGYDDTE
DSSCYIPPLTTIKQDFRLLGQTSVDRLLQLSQGQA
VKGNQLLPVSLVKRKTTLAPNTQTASPRALADSL

LacStop

VN** TLYDV AEYAGVSYQTVSRVVNQASHVSAKT
REKVEAAMAELNYIPNRVAQQLAGKQSLLIGVATS
SLALHAPSQIVAAIKSRADQLGASVVVSMVERSG
VEACKTAVHNLLAQRVSGLIINYPLDDQDAIAVEAA
CTNVPALFLDVSDQTPINSIIFSHEDGTRLGVEHLV
ALGHQQIALLAGPLSSVSARLRLAGWHKYLTRNQ
IQPIAEREGDWSAMSGFQQTMQMLNEGIVPTAML
VANDQMALGAMRAITESGLRVGADISVVGYDDTE
DSSCYIPPLTTIKQDFRLLGQTSVDRLLQLSQGQA
VKGNQLLPVSLVKRKTTLAPNTQTASPRALADSL

Supplementary Figure 2: Primary structures of 35 putative transcription factors and LacStop. Amino acid sequences for each of the X^{+}_{ADR} used in this work. The region between amino acids 10-30 is circled and residues 17,18, and 22 are depicted as red Xs to denote that they were changed throughout this work to yield the various ADR units, details shown in **Supplementary Fig. 1c**. The coding sequence for LacStop is also shown, with the STOP codons depicted as asterisks (* = TAA). The genetic architecture of the TF plasmids is presented along with a detailed sequence view showing the region from the promoter to the start codon (green text) of the coding sequence. Each TF was produced using either the lacI promoter or in the case of FruR, the lacIq promoter (blue inset) with no Ribosome Binding Site before the start codon (GTG). The single base pair difference between both promoters is shown in bold. The -35 and -10 hexamers are underlined. Details regarding the workflow for engineering non-natural transcription factors is given in **Fig. 1**. LacStop were used to determine maximum GFP output with a given operator element *in situ*, data given in **Supplementary Fig. 3**

Supplementary Figure 3 (part 1)

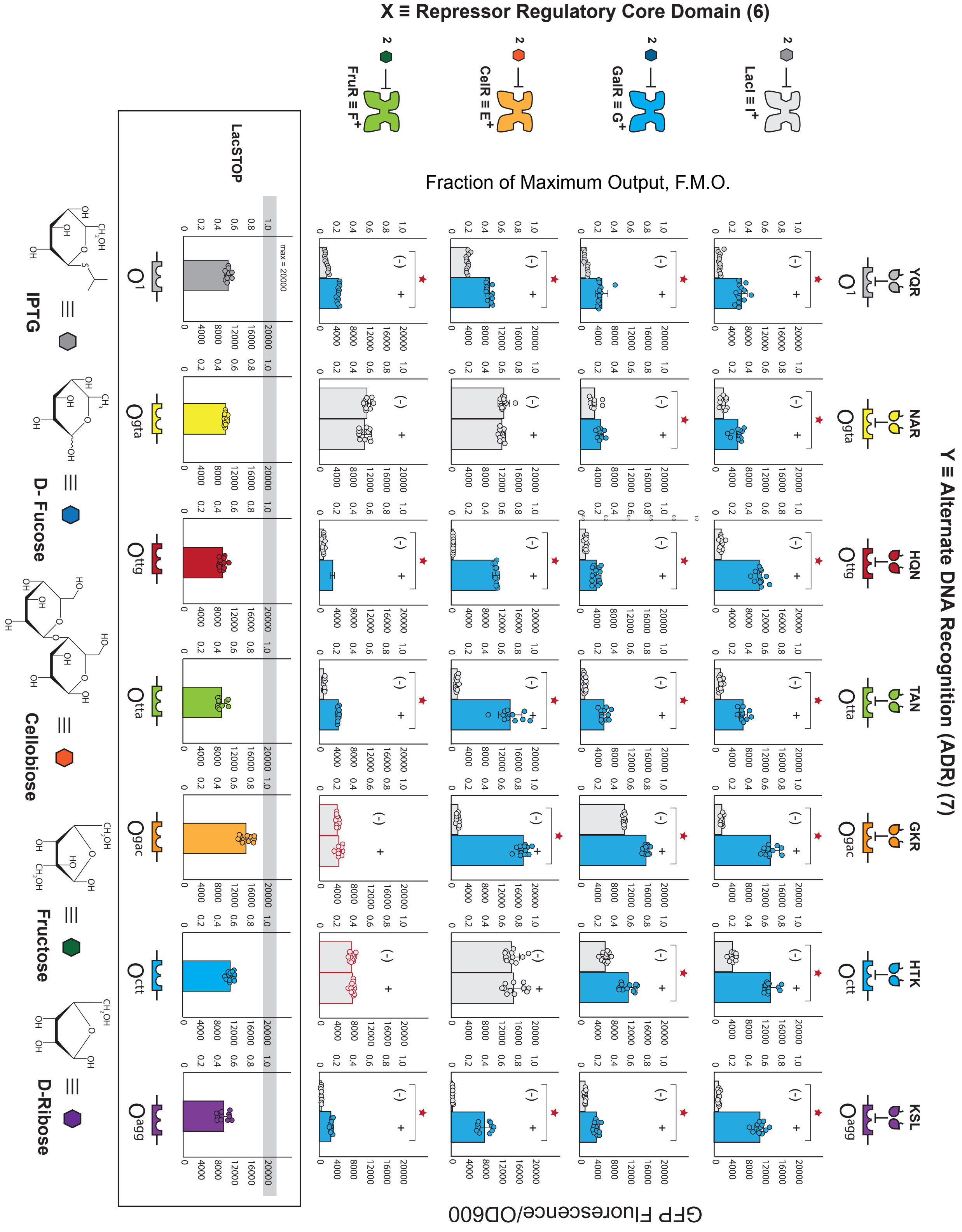

Supplementary Figure 3 (part 2)

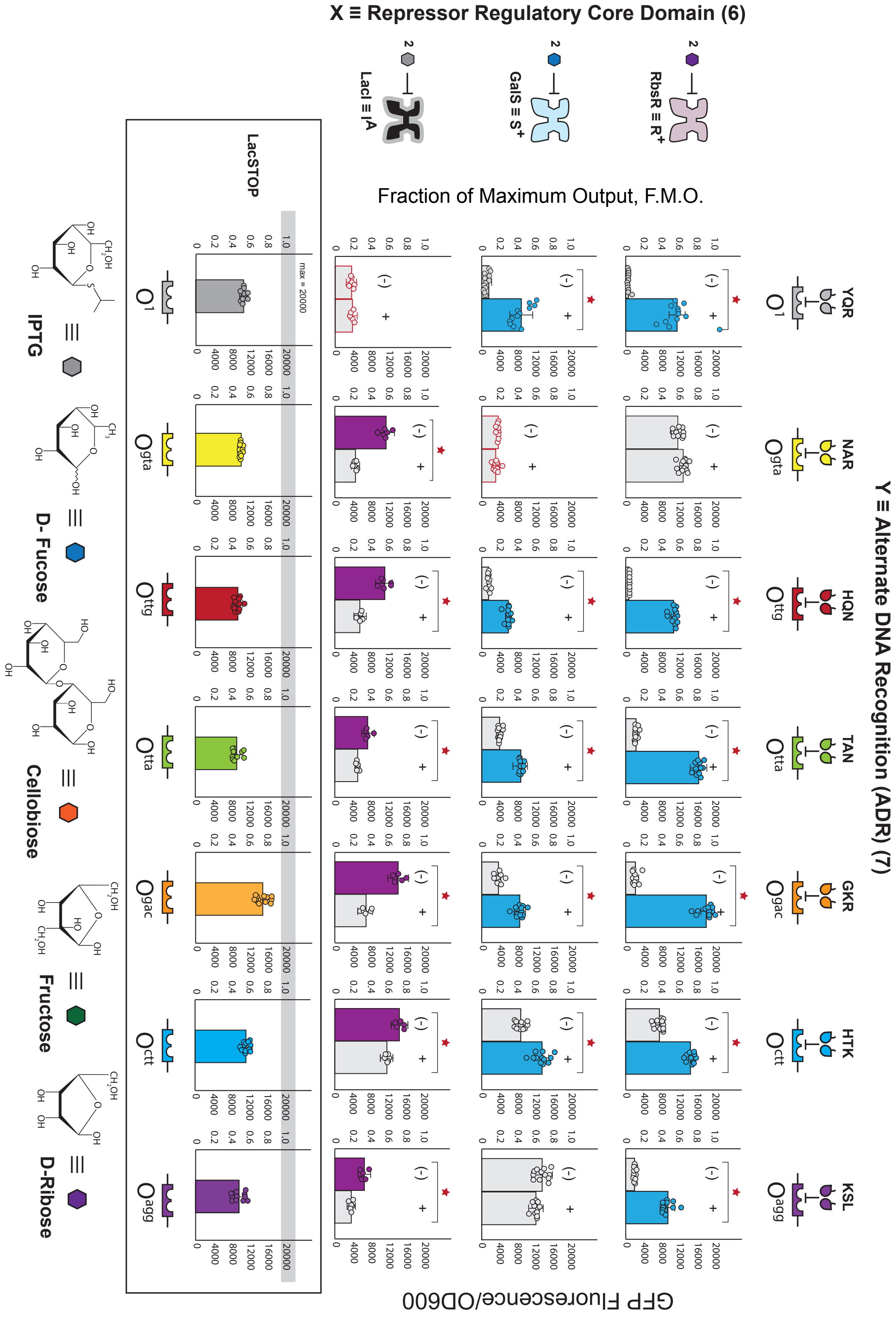

Supplementary Figure 3 (part 3)

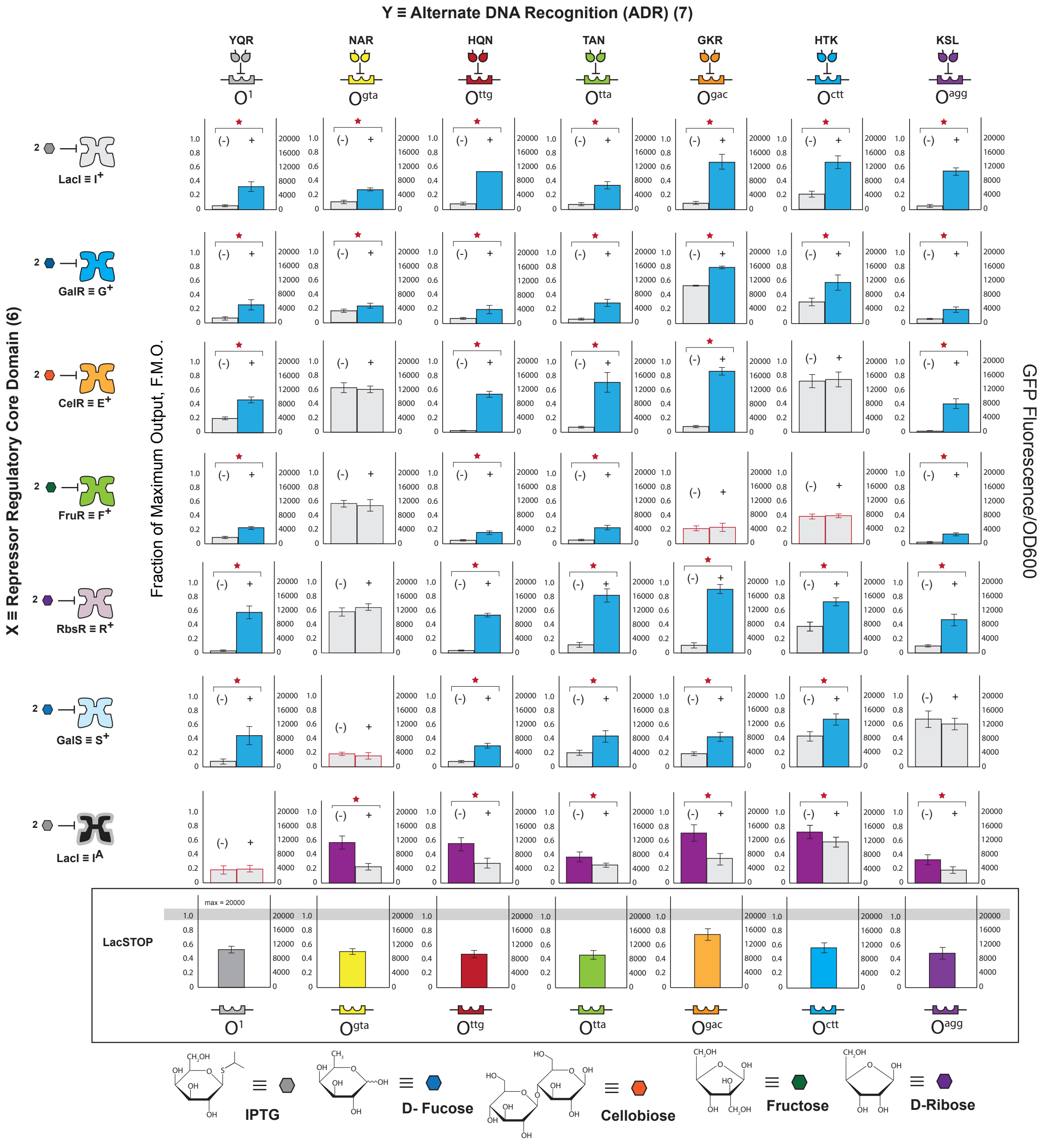

Supplementary Figure 3: Function of X_{ADR} Transcription Factors. Each set of histograms shows the level of GFP expression (-/+ inducer) for each of the 42 X_{ADR} variants along with their cognate operator. The Alternate DNA Recognition (ADR) units are shown along the top of the table, while the Regulatory Core Domains (RCD) and accompanying ligand are shown along the left. The red asterisks indicate statistical significance at the α<0.001 level. Each bar represents the average of n=12 biological replicates (except for the previously reported suppressors, where n = 6) and the error bars represent the 95% Confidence Interval (95% CI), which is approximately 2 times the standard error of the mean (s.e.m). The color of the bar in the presence of ligand is used to denote the phenotype observed in accordance with **Fig. 1**: blue bar indicates the X^+ phenotype, red bar indicates the X^s phenotype, purple bar indicates the X^A phenotype and gray bar indicates and X^- phenotype. Along the bottom row, the expression levels with LacStop are shown; these indicate the maximum observed GFP expression for each promoter-operator pair. Gray bar along the top is set at 20,000 arbitrary units (a.u.), which is the value used to normalize the data. Structure of each of the ligands along with their cartoon representation are shown along the bottom. The Fraction of Maximum Output (F.M.O.) is the normalized output value relative to the maximum LacSTOP values. These data are summarized in **Fig. 2** along the diagonal of each matrix.

Supplementary Figure 4

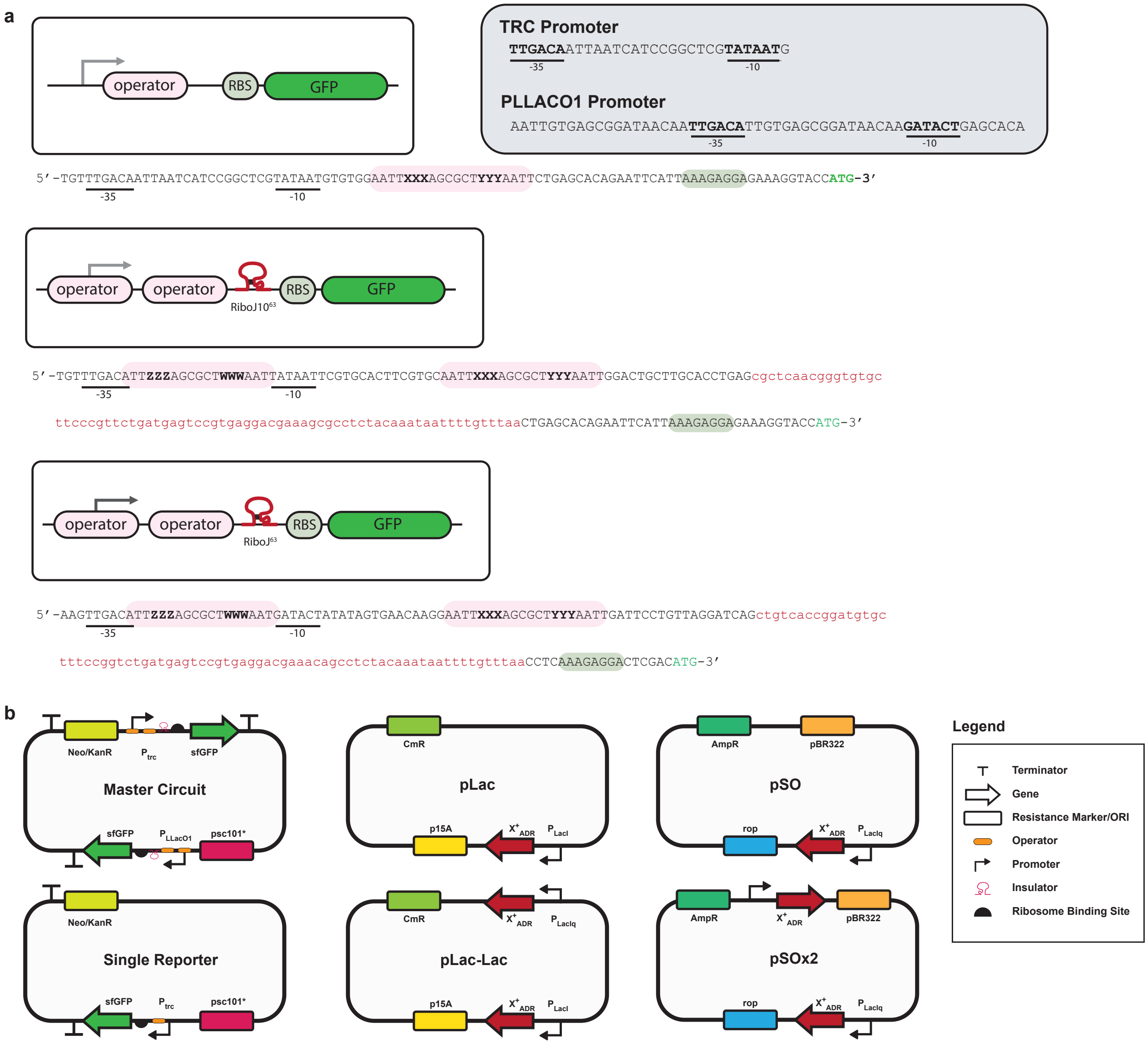

Supplementary Figure 4: Reporter and TF system layout and sequence information. (a) Schematic for the different reporter systems used in this work. The first is the single reporter used to collect data for **Fig. 2** along with a detailed sequence view. The -35 and -10 hexamers of the trc promoter are underlined and the operator position is marked with a pink oval. Ribosome Binding Site is circled in light green and the start codon (ATG) is in green. The next two illustrations show the genetic architecture of both channels of the Master Circuit, and represent a granular description of the SERI architecture outlined in **Fig. 4a**. Sequence of the insulator is shown in red. Finally, we show the two promoters used in this work, the TRC promoter and the P_{LLacO1} with their -35 and -10 hexamers underlined. **(b)** Plasmid maps for all plasmids used in this study detailing important structural features. Promoters are indicated with a bent arrow, operators with orange ovals, genes with a thick arrow, RBSs with a solid half-circle, terminators with a T and insulators with a small pink loop. Plasmid "backbone" features such as ORI/Resistance markers are shown as rounded rectangles. Details regarding the workflow for engineering non-natural transcription factors is given in **Fig. 1**. The sequences of all relevant plasmids are provided in GenBank – see **Data Availability** Section for relevant Accession Numbers.

Supplementary Figure 5

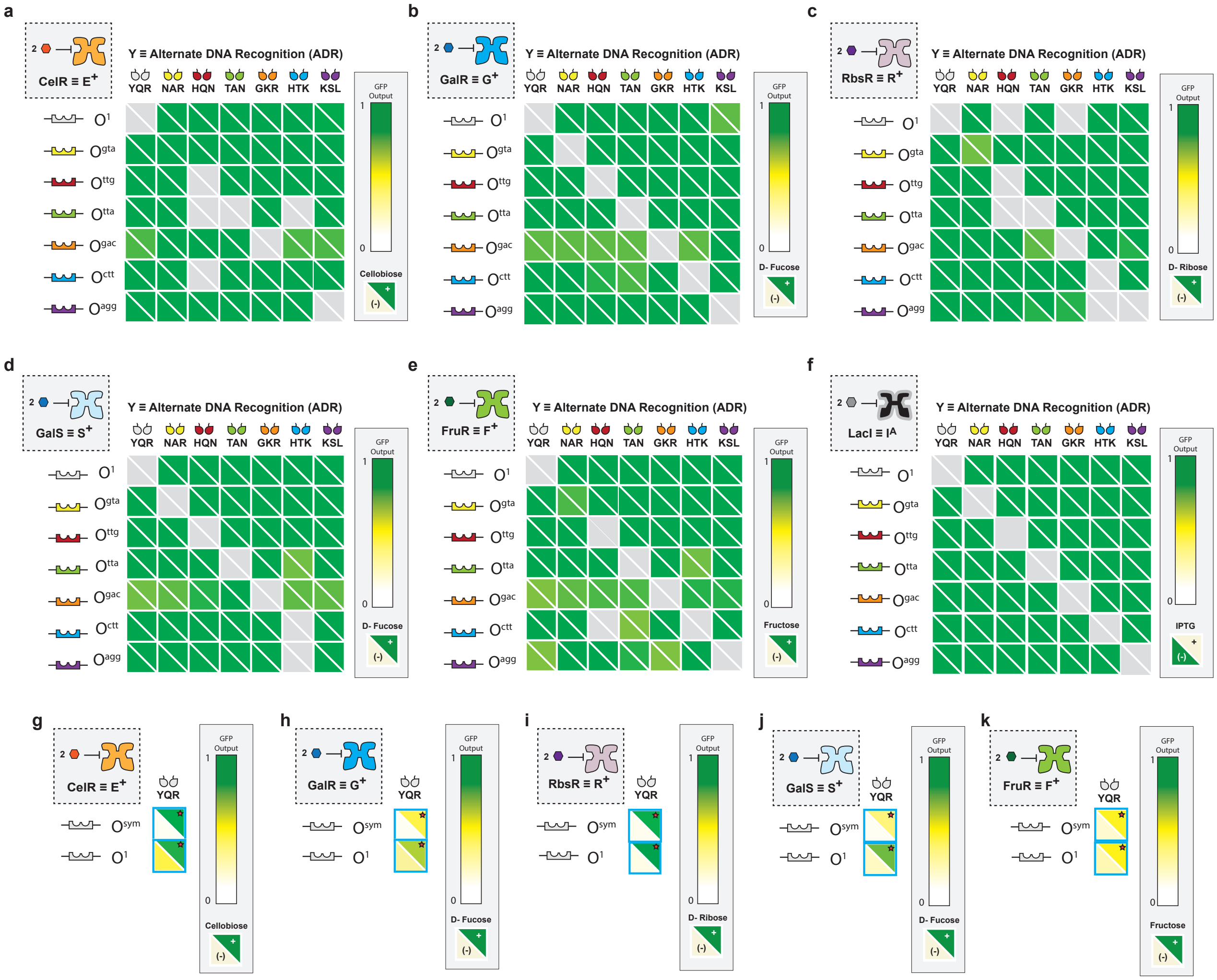

Supplementary Figure 5: Inverse matrices for unresponsive phenotypes. Any repressor-operator combination that was incapable of repression is defined as the X^- phenotype. This accounts for both variants that were non-functional as well as operator-repressor combinations in which the ADR fails to interact with the operator by design. **(a)** Cellobiose Repressor, CelR ≡ E **(b)** Galactose Repressor, GalR ≡ G **(c)** Ribose Repressor, RbsR, R **(d)** Galactose Isorepressor, GalS ≡ S **(e)** Fructose Repressor, FruR ≡ F along with a representative antilac **(f)** $I^{A(1)}$. The ADR DNA-binding domains are displayed along the top of the table and their corresponding operators are shown along the left. Scale bar (inset, right) shows a reference for GFP output. The bottom left triangle shows (OD_{600} normalized) GFP output in the absence of inducer, while the top right shows GFP output in the presence of 10mM of the inducer corresponding to a given RCD. Values correspond to the mean of n=12 biological replicates. These "inverse" matrices illustrate repression values for the X^- phenotypes, which were excluded (shown in gray) in **Fig. 2.** None of the transcription factor-operator pairs shown displayed a statistically significant difference in GFP expression upon addition of ligand at an alpha value of α=0.001 and all expressed GFP at greater than 50% of maximum signal for each operator. **(g-k)** Performance of each X^+_{YQR} on O^1 and O^{SYM}**.** For each of the X^+_{YQR} Transcription Factors, we show the performance on the $LacO^1$ operator, the pseudo-palindromic 21bp operator compared to the performance on O^{SYM}, the fully symmetric operator generated by mirroring the left half site (10bp) of the $LacO^1$operator. We show the performance of each of the RCDs on O^1 (main text **Fig. 2**), while the circuit designs utilized O^{SYM} since all other operators used in this study were symmetric and 20bp – this ensured greater modularity between parts when generating circuits. Red stars denote a statistically significant difference between the two states at α = 0.001 level using a one-tailed student's t-test. Values correspond to the mean of n=12 biological replicates.

Supplementary Figure 6 (part 1)

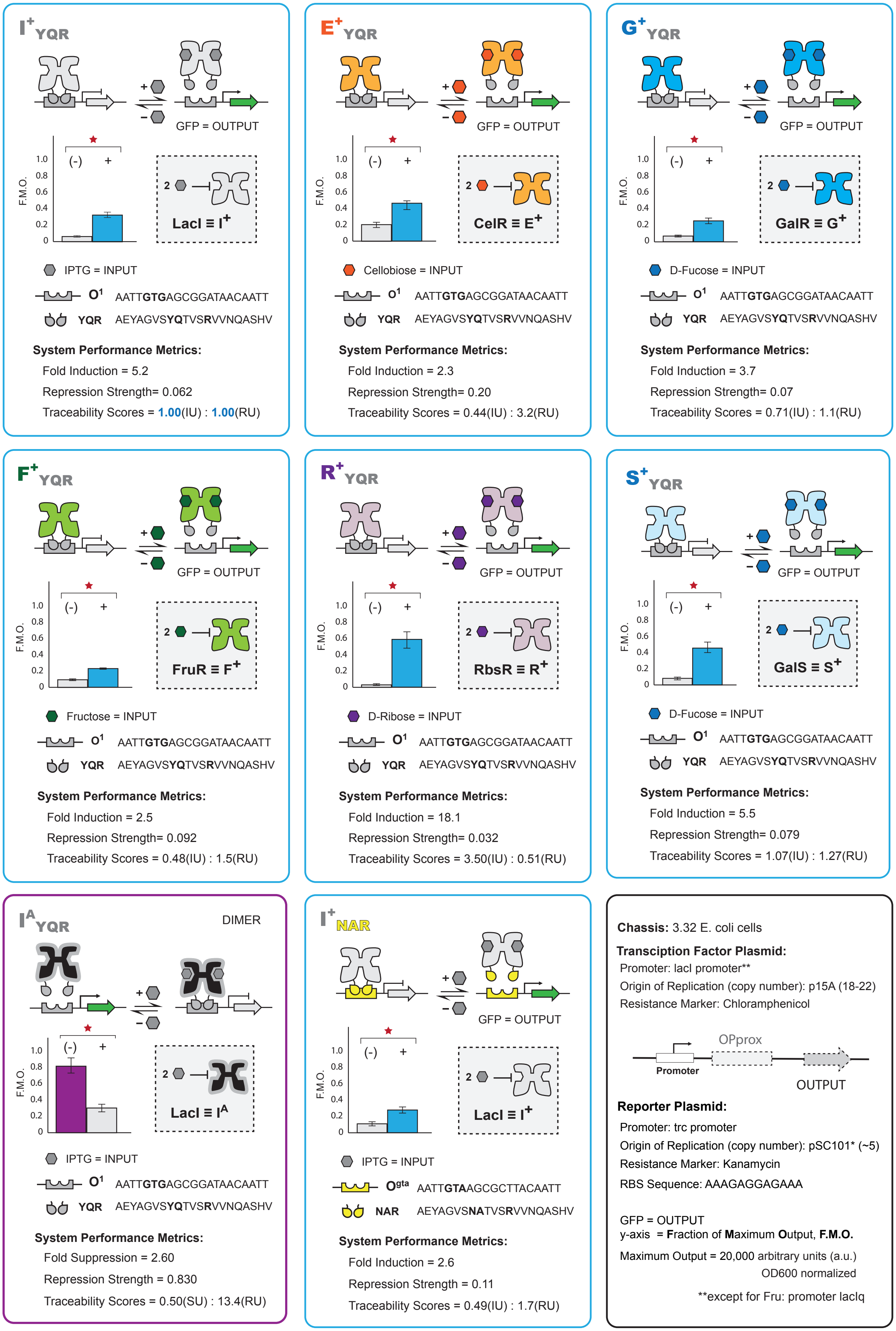

Supplementary Figure 6 (part 2)

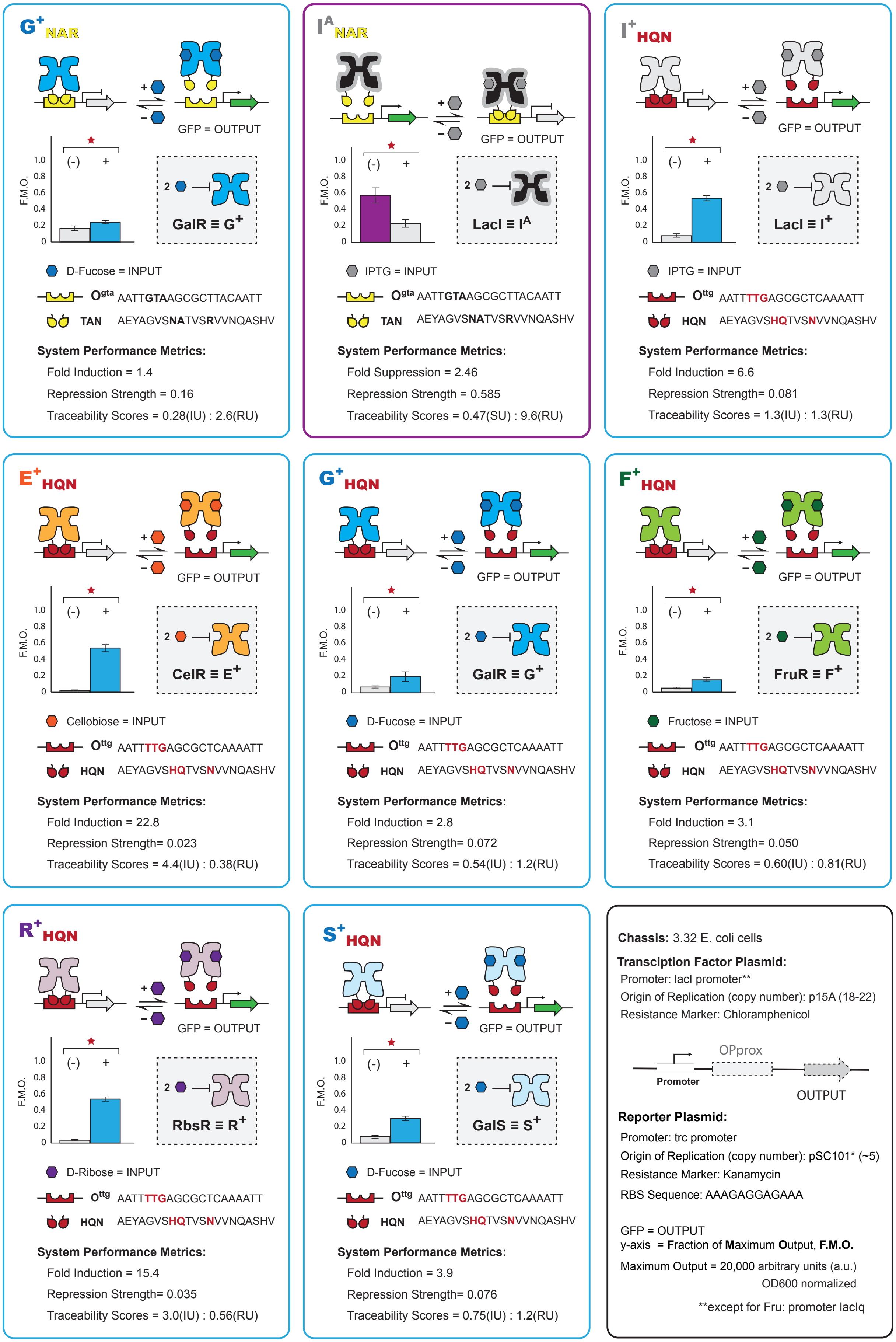

Supplementary Figure 6 (part 3)

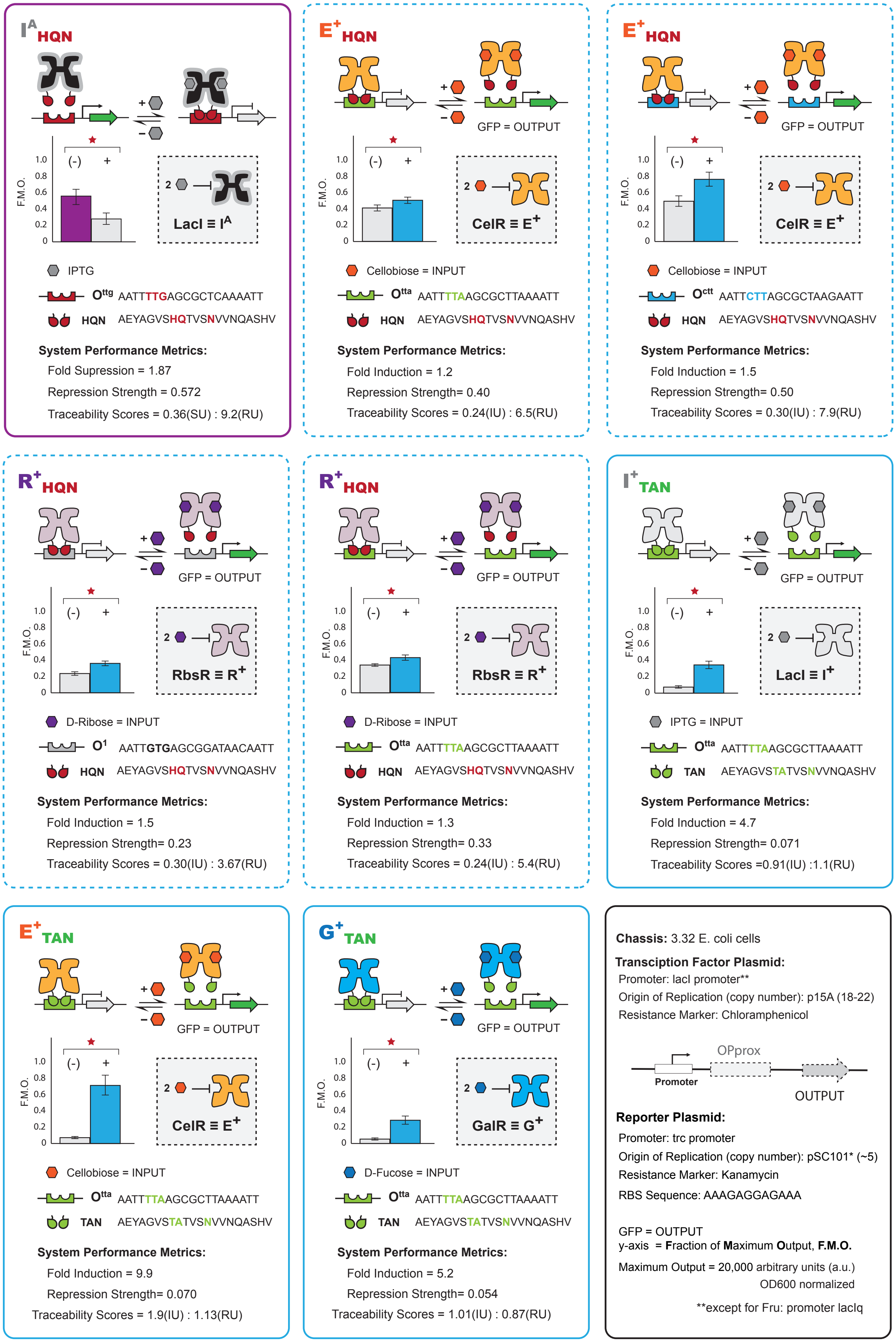

Supplementary Figure 6 (part 4)

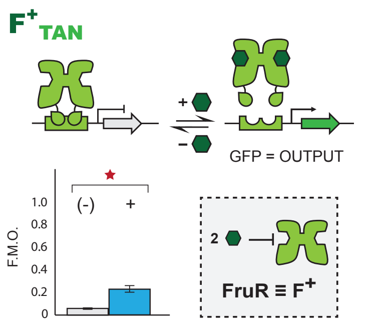

Fructose = INPUT

O^{tta} AATTTTAAGCGCTTAAAATT

TAN AEYAGVSTATVSNVVNQASHV

System Performance Metrics:

Fold Induction = 3.9

Repression Strength= 0.057

Traceability Scores = 0.76(IU) : 0.92(RU)

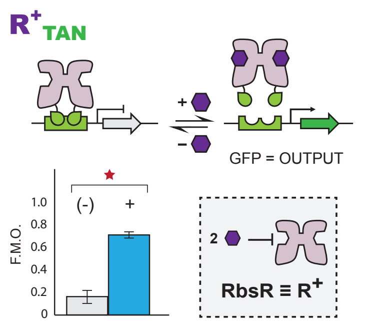

D-Ribose = INPUT

O^{tta} AATTTTAAGCGCTTAAAATT

TAN AEYAGVSTATVSNVVNQASHV

System Performance Metrics:

Fold Induction = 4.3

Repression Strength= 0.16

Traceability Scores = 0.83(IU) : 2.6(RU)

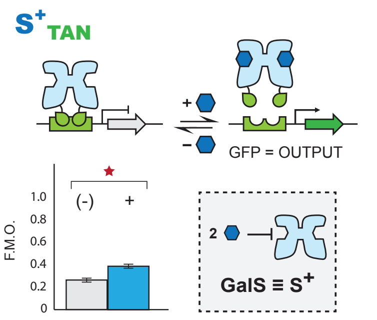

D-Fucose = INPUT

O^{tta} AATTTTAAGCGCTTAAAATT

TAN AEYAGVSTATVSNVVNQASHV

System Performance Metrics:

Fold Induction = 1.5

Repression Strength= 0.26

Traceability Scores = 0.28(IU) : 4.1(RU)

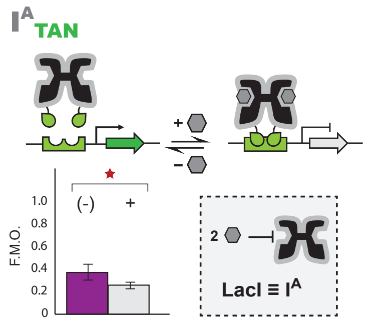

IPTG = INPUT

O^{tta} AATTTTAAGCGCTTAAAATT

TAN AEYAGVSTATVSNVVNQASHV

System Performance Metrics:

Fold Suppression = 1.41

Repression Strength = 0.372

Traceability Scores = 0.27(SU) : 6.0(RU)

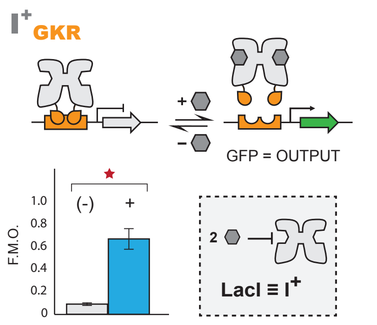

IPTG = INPUT

O^{gac} AATTGACAGCGCTGTCAATT

GKR AEYAGVSGKTVSRVVNQASHV

System Performance Metrics:

Fold Induction = 7.4

Repression Strength= 0.088

Traceability Scores = 1.44(IU) : 1.42(RU)

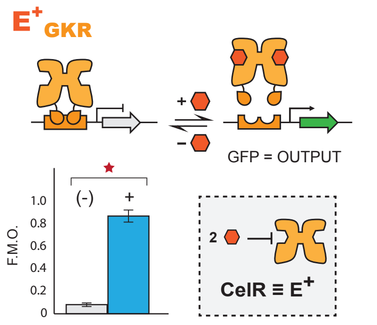

Cellobiose = INPUT

O^{gac} AATTGACAGCGCTGTCAATT

GKR AEYAGVSGKTVSRVVNQASHV

System Performance Metrics:

Fold Induction = 10.6

Repression Strength= 0.082

Traceability Scores = 2.04(IU) : 1.32(RU)

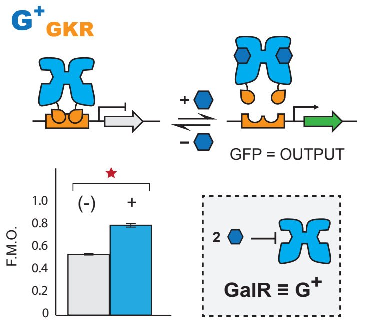

D-Fucose = INPUT

O^{gac} AATTGACAGCGCTGTCAATT

GKR AEYAGVSGKTVSRVVNQASHV

System Performance Metrics:

Fold Induction = 1.5

Repression Strength= 0.53

Traceability Scores = 0.28(IU) : 8.54(RU)

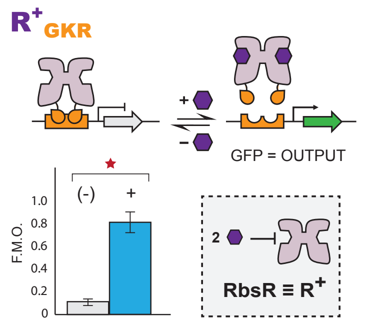

D-Ribose = INPUT

O^{gac} AATTGACAGCGCTGTCAATT

GKR AEYAGVSGKTVSRVVNQASHV

System Performance Metrics:

Fold Induction = 7.2

Repression Strength= 0.11

Traceability Scores = 1.39(IU) : 1.80(RU)

Chassis: 3.32 E. coli cells

Transciption Factor Plasmid:

Promoter: lacI promoter**

Origin of Replication (copy number): p15A (18-22)

Resistance Marker: Chloramphenicol

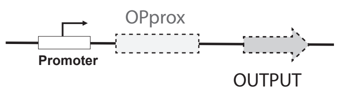

Reporter Plasmid:

Promoter: trc promoter

Origin of Replication (copy number): pSC101* (~5)

Resistance Marker: Kanamycin

RBS Sequence: AAAGAGGAGAAA

GFP = OUTPUT

y-axis = Fraction of **M**aximum **O**utput, **F.M.O.**

Maximum Output = 20,000 arbitrary units (a.u.) OD600 normalized

**except for Fru: promoter lacIq

Supplementary Figure 6 (part 5)

S^+ GKR

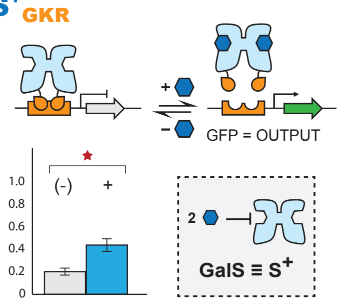

D-Fucose = INPUT

O^gac AATTGACAGCGCTGTCAATT

GKR AEYAGVSGKTVSRVVNQASHV

System Performance Metrics:

Fold Induction = 2.2

Repression Strength= 0.20

Traceability Scores = 0.42(IU) : 3.19(RU)

I^A GKR

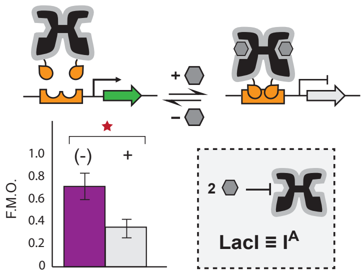

IPTG

O^gac AATTGACAGCGCTGTCAATT

GKR AEYAGVSGKTVSRVVNQASHV

System Performance Metrics:

Fold Supression = 1.87

Repression Strength = 0.73

Traceability Scores = 0.36(SU) : 11.8(RU)

R^+ GKR

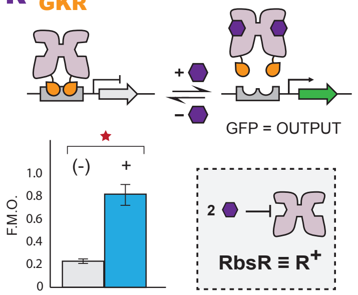

D-Ribose = INPUT

O^1 AATTGTGAGCGGATAACAATT

GKR AEYAGVSGKTVSRVVNQASHV

System Performance Metrics:

Fold Induction = 3.55

Repression Strength= 0.22

Traceability Scores = 0.69(IU) : 3.67(RU)

I^+ HTK

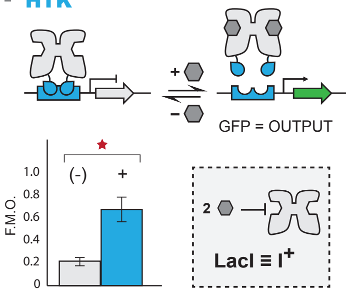

IPTG = INPUT

O^ctt AATTCTTAGCGCTAAGAATT

HTK AEYAGVSHTTVSKVVNQASHV

System Performance Metrics:

Fold Induction = 3.1

Repression Strength= 0.21

Tracablity Scores = 0.61(IU) : 3.4(RU)

G^+ HTK

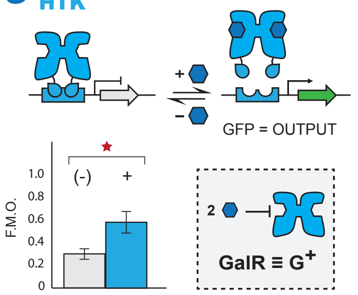

D-Fucose = INPUT

O^ctt AATTCTTAGCGCTAAGAATT

HTK AEYAGVSHTTVSKVVNQASHV

System Performance Metrics:

Fold Induction = 1.9

Repression Strength= 0.30

Tracablity Scores = 0.37(IU) : 4.8(RU)

R^+ HTK

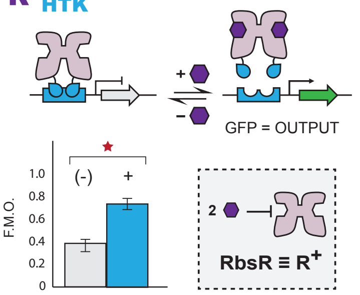

D-Ribose = INPUT

O^ctt AATTCTTAGCGCTAAGAATT

HTK AEYAGVSHTTVSKVVNQASHV

System Performance Metrics:

Fold Induction = 1.9

Repression Strength= 0.37

Tracablity Scores = 0.37(IU) : 6.0(RU)

S^+ HTK

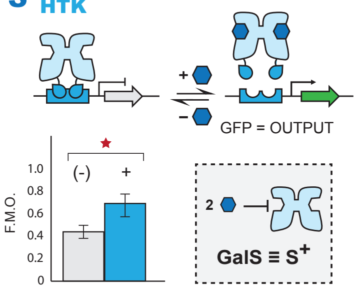

D-Fucose = INPUT

O^ctt AATTCTTAGCGCTAAGAATT

HTK AEYAGVSHTTVSKVVNQASHV

System Performance Metrics:

Fold Induction = 1.6

Repression Strength= 0.31

Tracablity Scores = 0.43(IU) : 6.9(RU)

I^A HTK

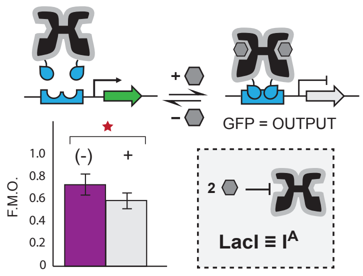

IPTG = INPUT

O^ctt AATTCTTAGCGCTAAGAATT

HTK AEYAGVSHTTVSKVVNQASHV

System Performance Metrics:

Fold Suppression = 1.19

Repression Strength = 0.74

Traceability Scores = 0.23(SU) : 11.9(RU)

Chassis: 3.32 E. coli cells

Transciption Factor Plasmid:

Promoter: lacI promoter**

Origin of Replication (copy number): p15A (18-22)

Resistance Marker: Chloramphenicol

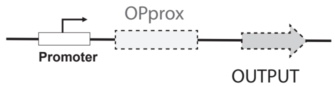

Reporter Plasmid:

Promoter: trc promoter

Origin of Replication (copy number): pSC101* (~5)

Resistance Marker: Kanamycin

RBS Sequence: AAAGAGGAGAAA

GFP = OUTPUT

y-axis = **F**raction of **M**aximum **O**utput, **F.M.O.**

Maximum Output = 20,000 arbitrary units (a.u.)
OD600 normalized

**except for Fru: promoter lacIq

Supplementary Figure 6 (part 6)

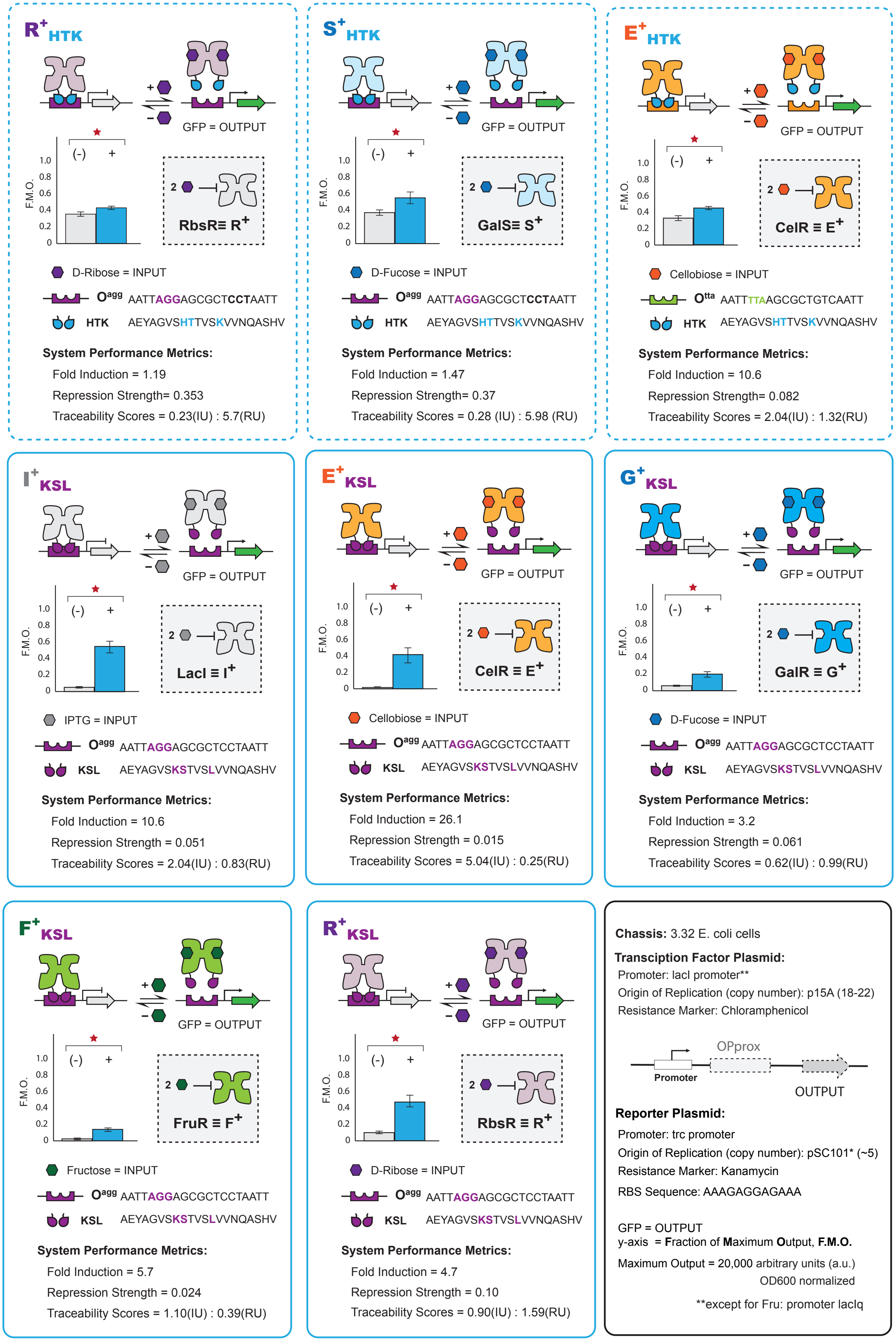

Supplementary Figure 6 (part 7)

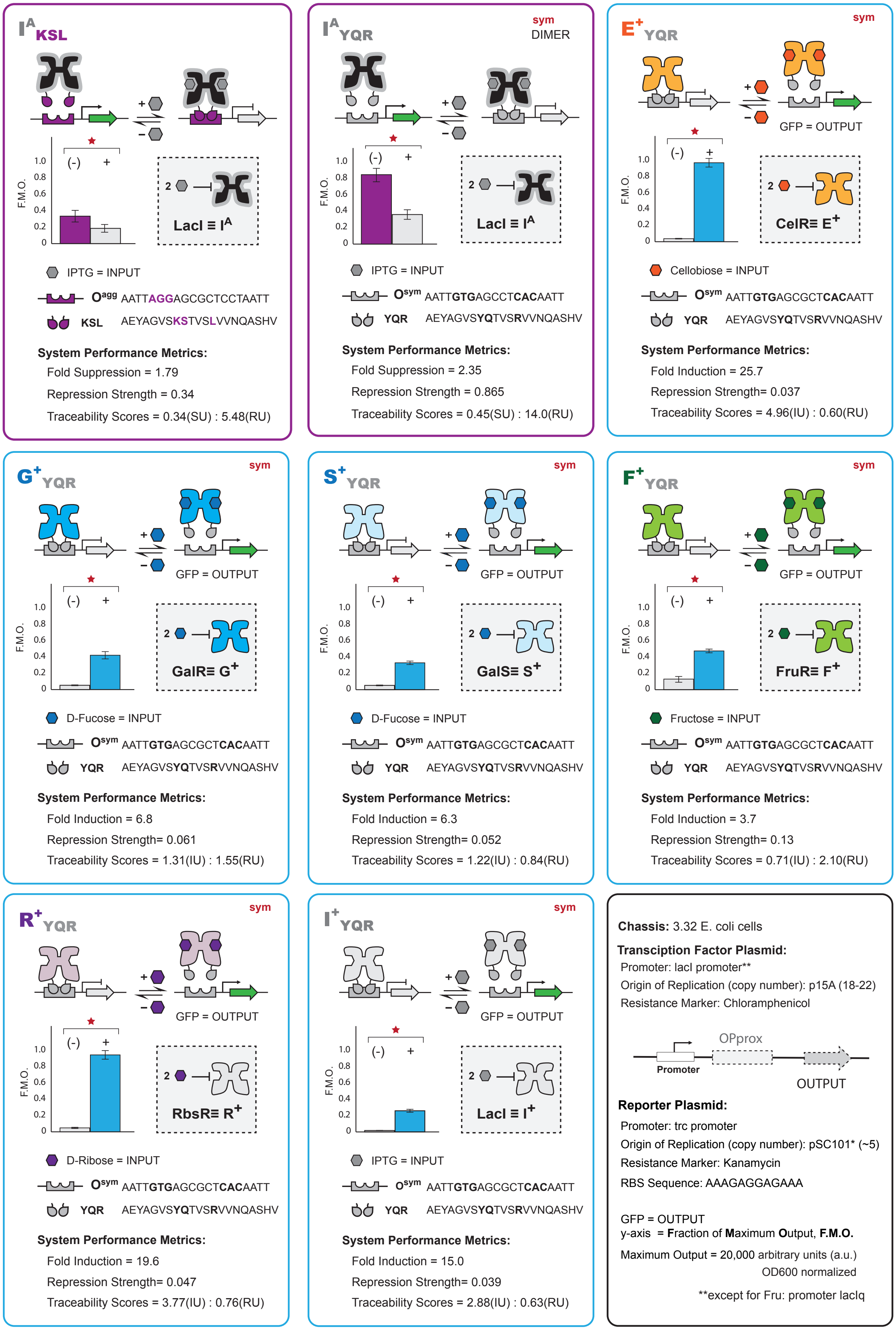

Supplementary Figure 6 (part 8)

E^+ HQN

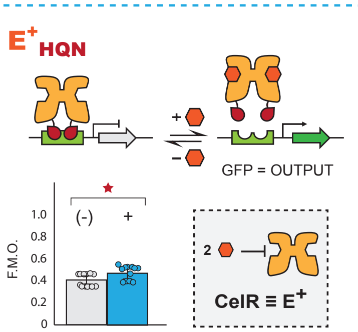

Cellobiose = INPUT

O^{tta} AATTTTAAGCGCTTAAAATT

HQN AEYAGVSHQTVSNVVNQASHV

System Performance Metrics:

Fold Induction = 1.2

Repression Strength= 0.40

Traceability Scores = 0.24(IU) : 6.5(RU)

E^+ HQN

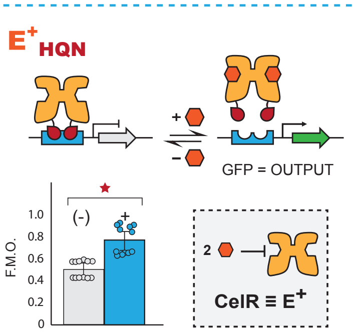

Cellobiose = INPUT

O^{ctt} AATTCTTAGCGCTAAGAATT

HQN AEYAGVSHQTVSNVVNQASHV

System Performance Metrics:

Fold Induction = 1.5

Repression Strength= 0.50

Traceability Scores = 0.30(IU) : 7.9(RU)

R^+ HQN

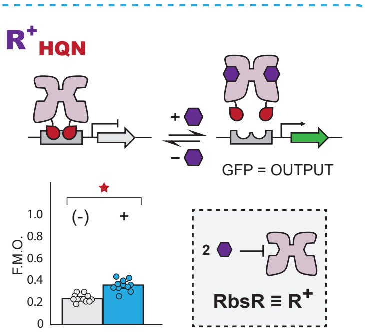

D-Ribose = INPUT

O^1 AATTGTGAGCGGATAACAATT

HQN AEYAGVSHQTVSNVVNQASHV

System Performance Metrics:

Fold Induction = 1.5

Repression Strength= 0.23

Traceability Scores = 0.30(IU) : 3.67(RU)

R^+ HQN

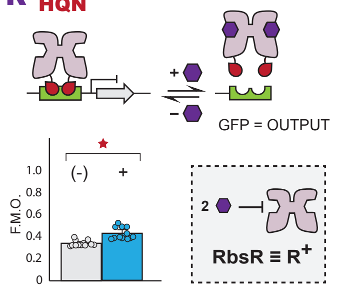

D-Ribose = INPUT

O^{tta} AATTTTAAGCGCTTAAAATT

HQN AEYAGVSHQTVSNVVNQASHV

System Performance Metrics:

Fold Induction = 1.3

Repression Strength= 0.33

Traceability Scores = 0.24(IU) : 5.4(RU)

R^+ GKR

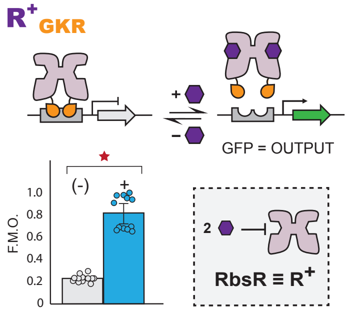

D-Ribose = INPUT

O^1 AATTGTGAGCGGATAACAATT

GKR AEYAGVSGKTVSRVVNQASHV

System Performance Metrics:

Fold Induction = 3.55

Repression Strength= 0.22

Traceability Scores = 0.69(IU) : 3.67(RU)

R^+ HTK

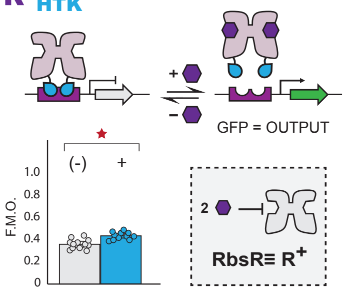

D-Ribose = INPUT

O^{agg} AATTAGGAGCGCTCCTAATT

HTK AEYAGVSHTTVSKVVNQASHV

System Performance Metrics:

Fold Induction = 1.19

Repression Strength= 0.353

Traceability Scores = 0.23(IU) : 5.7(RU)

S^+ HTK

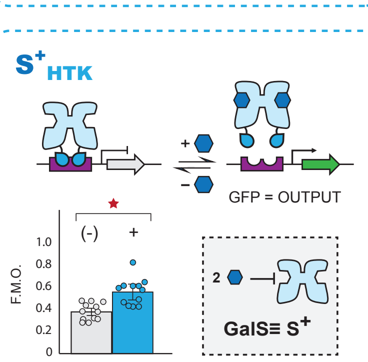

D-Fucose = INPUT

O^{agg} AATTAGGAGCGCTCCTAATT

HTK AEYAGVSHTTVSKVVNQASHV

System Performance Metrics:

Fold Induction = 1.47

Repression Strength= 0.37

Traceability Scores = 0.28 (IU) : 5.98 (RU)

E^+ HTK

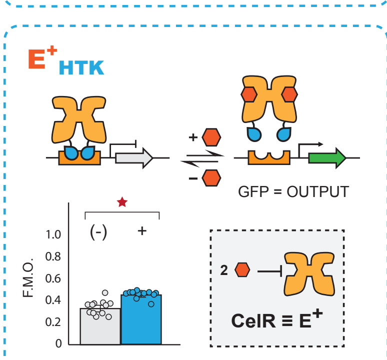

Cellobiose = INPUT

O^{tta} AATTTTAAGCGCTGTCAATT

HTK AEYAGVSHTTVSKVVNQASHV

System Performance Metrics:

Fold Induction = 10.6

Repression Strength= 0.082

Traceability Scores = 2.04(IU) : 1.32(RU)

Chassis: 3.32 E. coli cells

Transciption Factor Plasmid:

Promoter: lacI promoter**

Origin of Replication (copy number): p15A (18-22)

Resistance Marker: Chloramphenicol

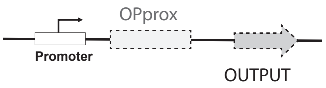

Reporter Plasmid:

Promoter: trc promoter

Origin of Replication (copy number): pSC101* (~5)

Resistance Marker: Kanamycin

RBS Sequence: AAAGAGGAGAAA

GFP = OUTPUT

y-axis = **F**raction of **M**aximum **O**utput, **F.M.O.**

Maximum Output = 20,000 arbitrary units (a.u.)
OD600 normalized

**except for Fru: promoter lacIq

Supplementary Figure 6 (part 9)

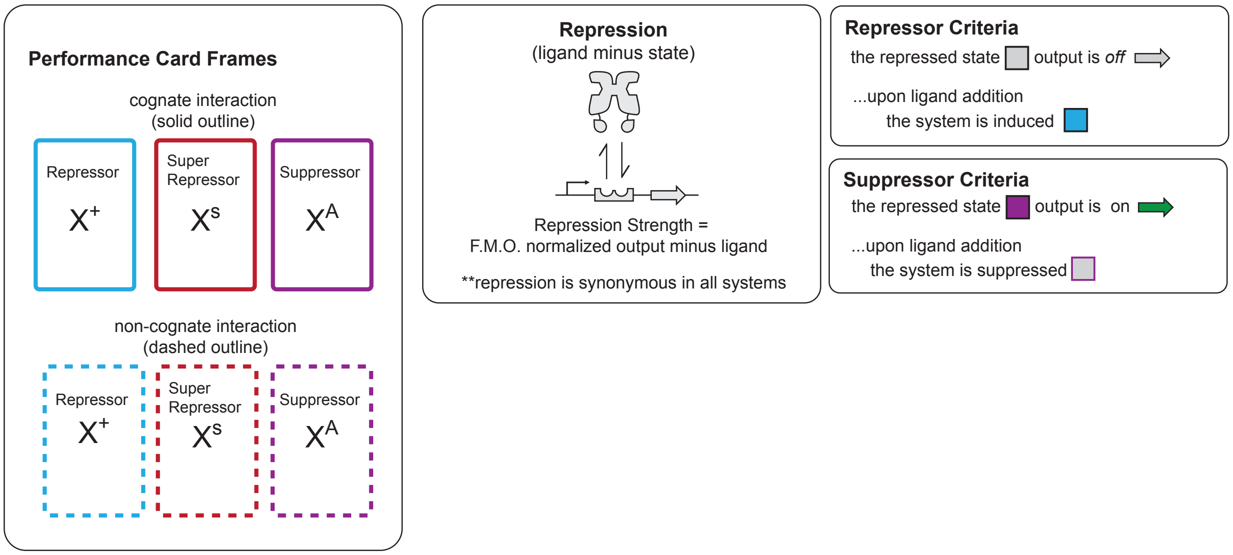

Supplementary Figure 6: Performance cards for each of the functional transcription factors. For each engineered transcription factor that exhibits either the X^+ phenotype (or X^A for suppressors), GFP output in the absence and presence of inducer is shown. Sequence of the DNA operator along with the primary protein sequence of the DNA Binding Domain with positions 17, 18, and 22 highlighted. Performance metrics are outlined above detailing the Fold Induction (Maximum GFP output/Minimum GFP output), Repression Strength (normalized GFP output in the repressed state), and Traceability Scores. The Traceability Score relates the performance of any X^+_{ADR} to the reference, which is designated as the I^+_{YQR} | O^1 pair – a value of less than 1 IU indicates induction less than the reference pair, while a value of greater than 1 RU indicates that a given system represses less tightly than the reference pair. Each page also includes information on the chassis including the type of cell used (*E. Coli* 3.32), the genetic architecture of the reporter used, and information about each plasmid. Examples of select engineered transcription factors used to construct basic logical operations and combinatorial logical programs are illustrated in **Figs. 3,5 and 6**, respectively. The color along the outline of the card denotes the phenotype in accordance with the color scheme outlined in **Fig. 1**. Solid lines indicate cognate (on-diagonal) interactions, while dashed lines indicate non-cognate (off-diagonal, or 'promiscuous') interactions. F.M.O. (Fraction of Maximum Output) for both the repressor and suppressor phenotype along with IU (Induction Units), RU (Repression Units), and Traceability Scores are explained in detail in **Supplementary Note 2**. Statistical analysis and related data are given in **Supplementary Data 2**.

Supplementary Note 2:

Metrics for Non-natural X^+_{ADR} Repressors. The Fraction of Maximum Output (F.M.O.) ≡ [GFP/OD600] / [Max LacSTOP value**], where (i) F.M.O. repression is the system minus ligand, and (ii) F.M.O. induction is the system plus ligand. Repression Strength ≡ F.M.O. normalized output minus ligand, and Fold Induction ≡ FI, such that:

$$\mathrm{FI} = \frac{(\mathrm{F.M.O.\ Induction})}{(\mathrm{F.M.O.\ Repression})} \tag{1}$$

Part 1 of the traceability score is given in terms of the induced state (where IU = Induction Units) such that the IU traceability scores were calculated as follows:

$$\text{IU Traceability Score} = \frac{\text{FI}(\text{X}^{+}_{\text{ADR}}|\text{OP})}{\text{FI}(\text{I}^{+}_{\text{YQR}}|\text{O}^{1})} \tag{2}$$

$$\text{IU Reference Score} = \frac{\text{FI}(\text{I}^{+}_{\text{YQR}}|\text{O}^{1})}{\text{FI}(\text{I}^{+}_{\text{YQR}}|\text{O}^{1})} = 1 \tag{3}$$

Part 2 of the traceability score is given in terms of the repressed state (where RU = Repression Units) such that the RU traceability scores were calculated as follows:

$$\text{RU Traceability Score} = \frac{\text{F.M.O. Repression }(\text{X}^{+}_{\text{ADR}}|\text{OP})}{\text{F.M.O. Repression }(\text{I}^{+}_{\text{YQR}}|\text{O}^{1})} \tag{4}$$

$$\text{RU Reference Score} = \frac{\text{F.M.O. Repression }(\text{I}^{+}_{\text{YQR}}|\text{O}^{1})}{\text{F.M.O. Repression }(\text{I}^{+}_{\text{YQR}}|\text{O}^{1})} = 1 \tag{5}$$

**Max LacSTOP value = 20,000 arbitrary units (a.u.), OD600 normalized.

Metrics for Non-natural I^{A}_{ADR} Suppressors. The Fraction of Maximum Output (F.M.O.) ≡ [GFP/OD600] / [Max LacSTOP value**], where (i) F.M.O. repression is the system minus ligand, and (ii) F.M.O. suppression is the system plus ligand. Repression Strength ≡ F.M.O. normalized output minus ligand, and Fold Suppression ≡ FS, such that:

$$\text{FS} = \frac{(\text{F.M.O. Repression})}{(\text{F.M.O. Suppression})} \tag{6}$$

Part 1 of the traceability score is given in terms of the suppressed state (where SU = Suppression Units) such that the SU traceability scores were calculated as follows:

$$\text{SU Traceability Score} = \frac{\text{FS}(\text{X}^{\text{A}}_{\text{ADR}}|\text{OP})}{\text{FI}(\text{I}^{+}_{\text{YQR}}|\text{O}^{1})} \tag{7}$$

$$\text{SU Reference Score} = \frac{\text{FI}(\text{I}^{+}_{\text{YQR}}|\text{O}^{1})}{\text{FI}(\text{I}^{+}_{\text{YQR}}|\text{O}^{1})} = 1 \tag{8}$$

Part 2 of the traceability score is given in terms of the repressed state (where RU = Repression Units) such that the RU traceability scores for a suppressor were calculated as follows:

$$\text{RU Traceability Score} = \frac{\text{F.M.O. Repression }(\text{X}^{\text{A}}_{\text{ADR}}|\text{OP})}{\text{F.M.O. Repression }(\text{I}^{+}_{\text{YQR}}|\text{O}^{1})} \tag{9}$$

$$\text{RU Reference Score} = \frac{\text{F.M.O. Repression }(\text{I}^{+}_{\text{YQR}}|\text{O}^{1})}{\text{F.M.O. Repression }(\text{I}^{+}_{\text{YQR}}|\text{O}^{1})} = 1 \tag{10}$$

**Max LacSTOP value = 20,000 arbitrary units (a.u.), OD600 normalized

Supplementary Figure 7

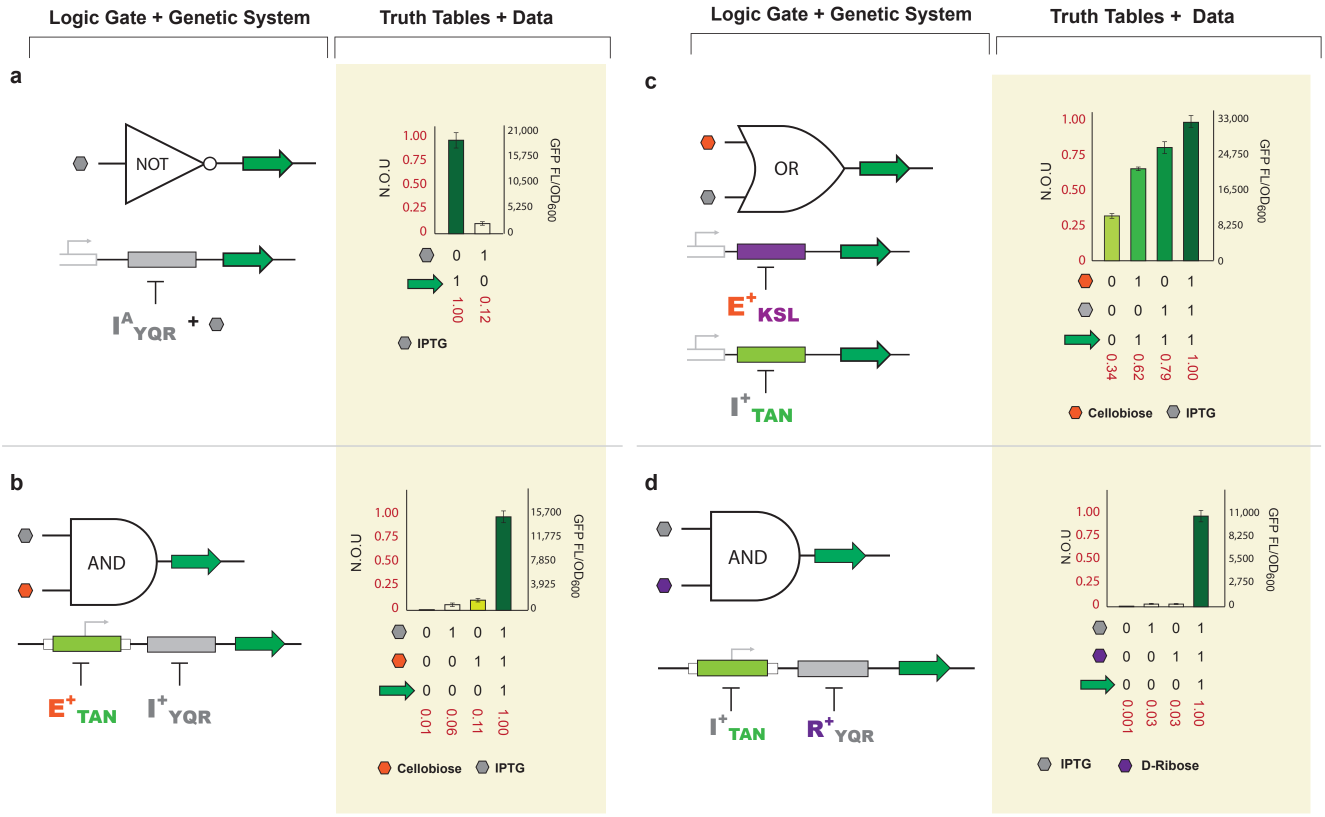

Supplementary Figure 7a

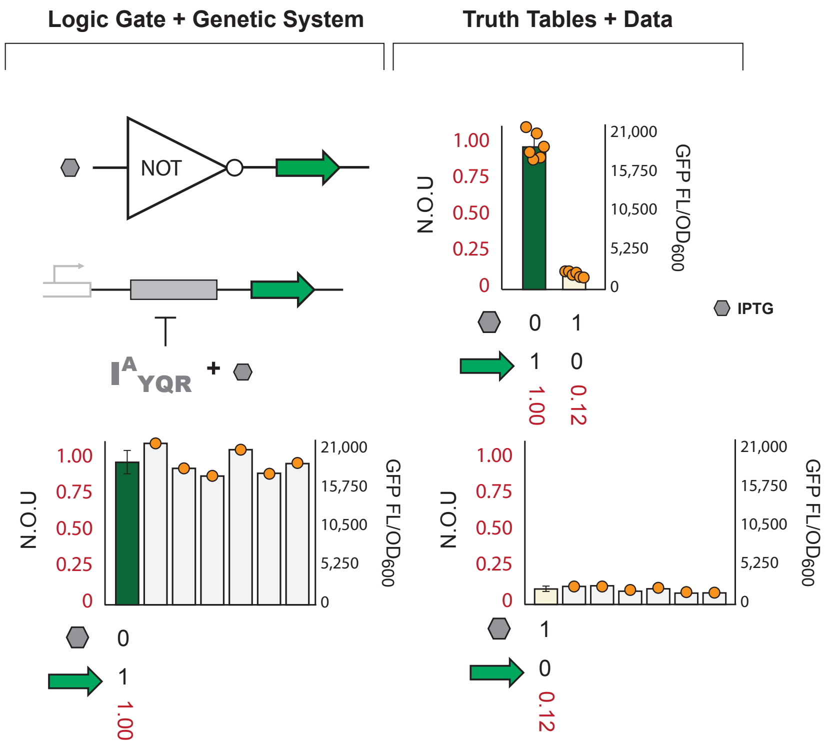

Supplementary Figure 7b

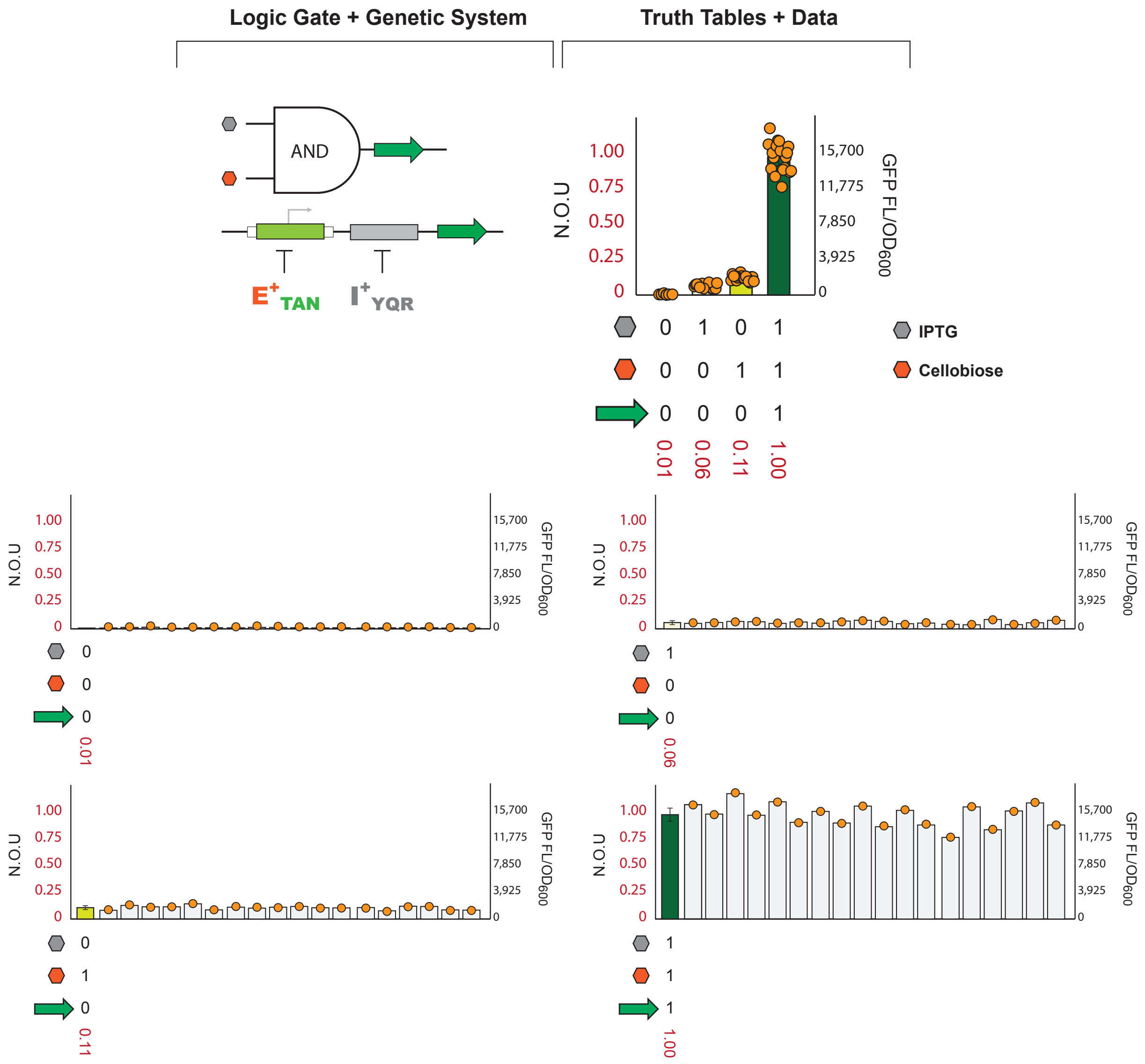

Supplementary Figure 7c

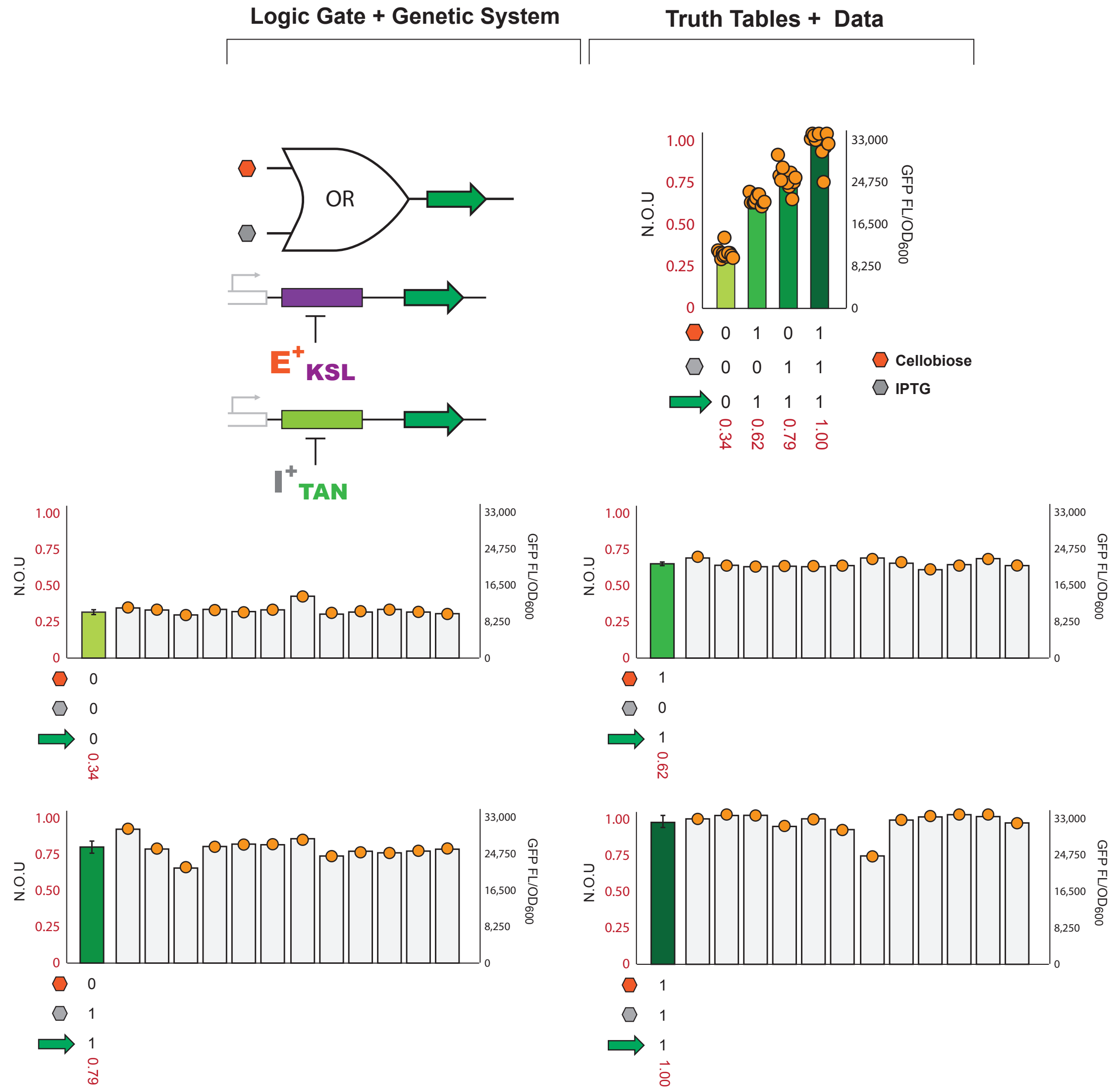

Supplementary Figure 7d

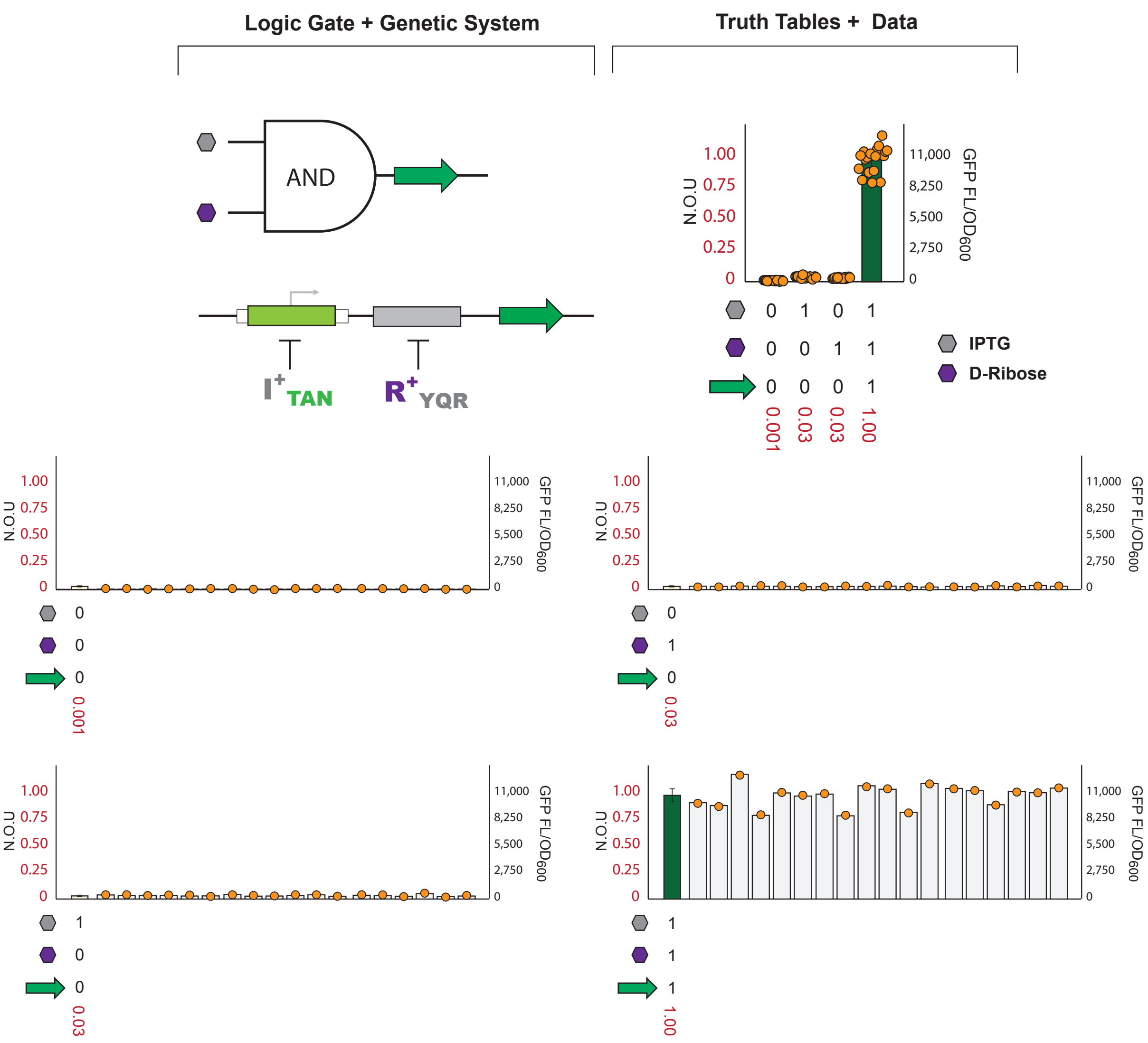

Supplementary Figure 7: Raw data NOT, AND, and OR logical gates. The second y-axis on the right shows the raw GFP Fluorescence/OD_{600} values that have been normalized to N.O.U. Y-axis (left) is given as normalized output units or N.O.U. This is calculated by taking GFP Fluorescence (485ex., 510em.) normalized to OD_{600}, which is further normalized to the maximum output for each experiment. Fractional expression values are shown in red under the truth tables **(a)** An engineered LacI suppressor ('antilac') represents a unary NOT gate – the output is OFF when IPTG is present. **(b)** A biological AND gate is achieved using the SERI architecture utilizing two X^{+}_{ADR} with orthogonal DNA recognition and ligand response. **(c)** Biological OR function is achieved through PARA architecture where each copy of GFP is controlled by a distinct X^{+}_{ADR}. **(d)** Another iteration of a biological AND gate utilizing distinct input signals, *via* two non-natural transcription factors. Data complementary to **Fig. 5a-d**.

Supplementary Figure 8

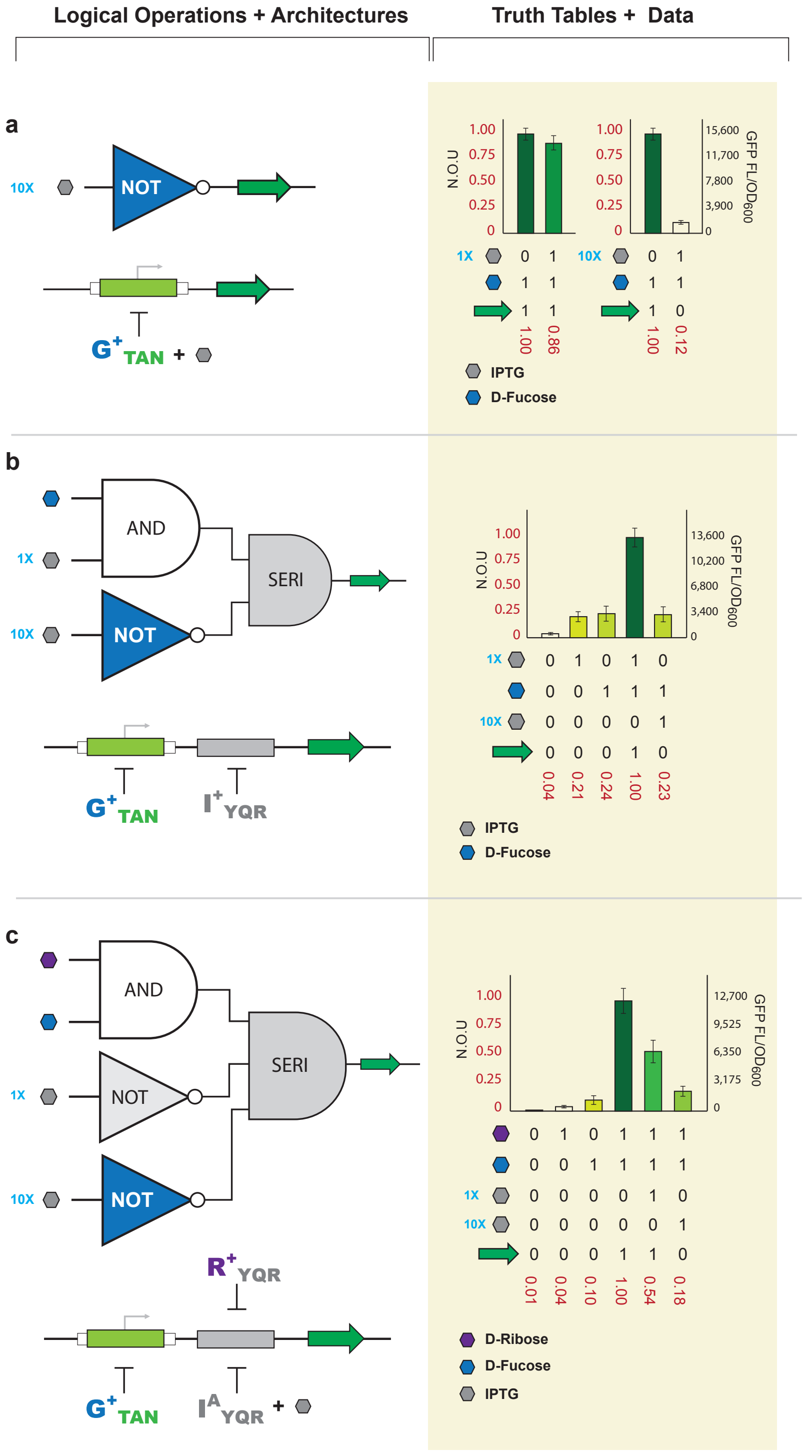

Supplementary Figure 8a

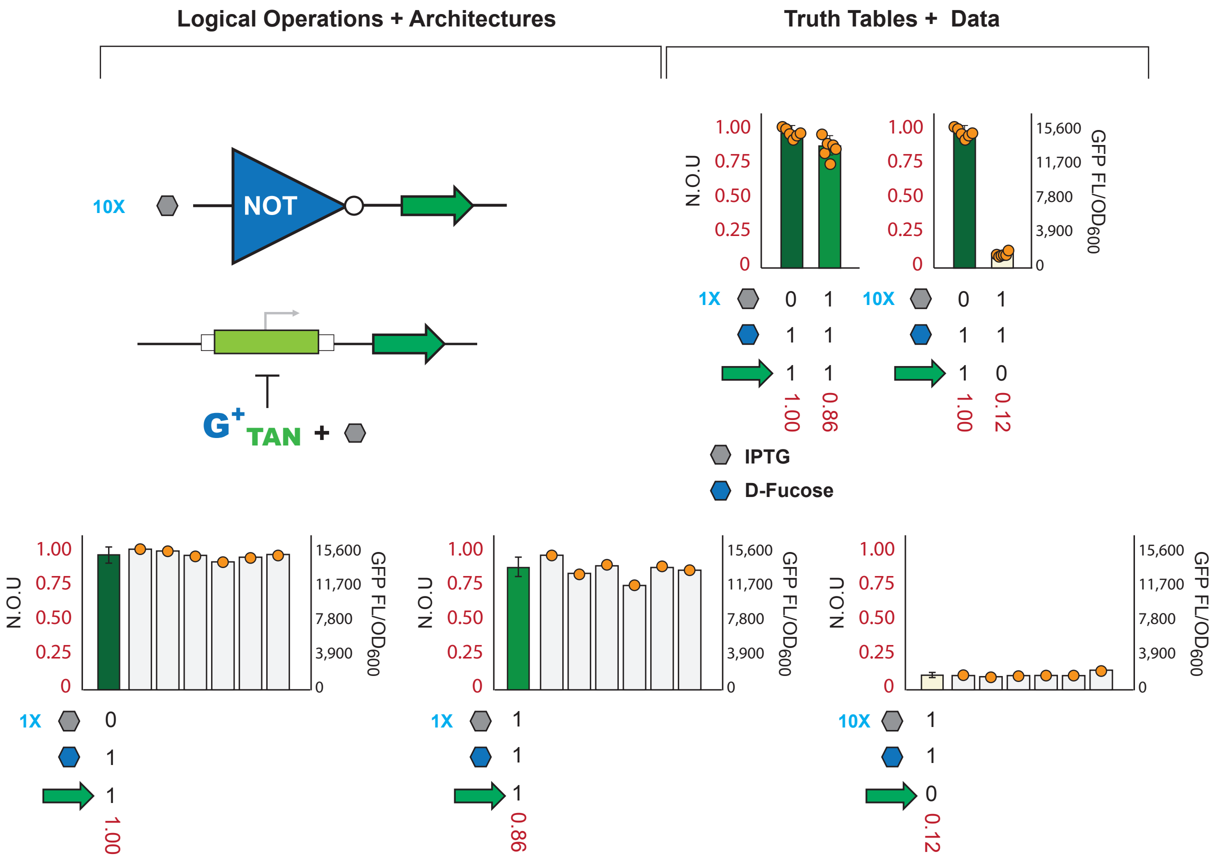

Supplementary Figure 8b

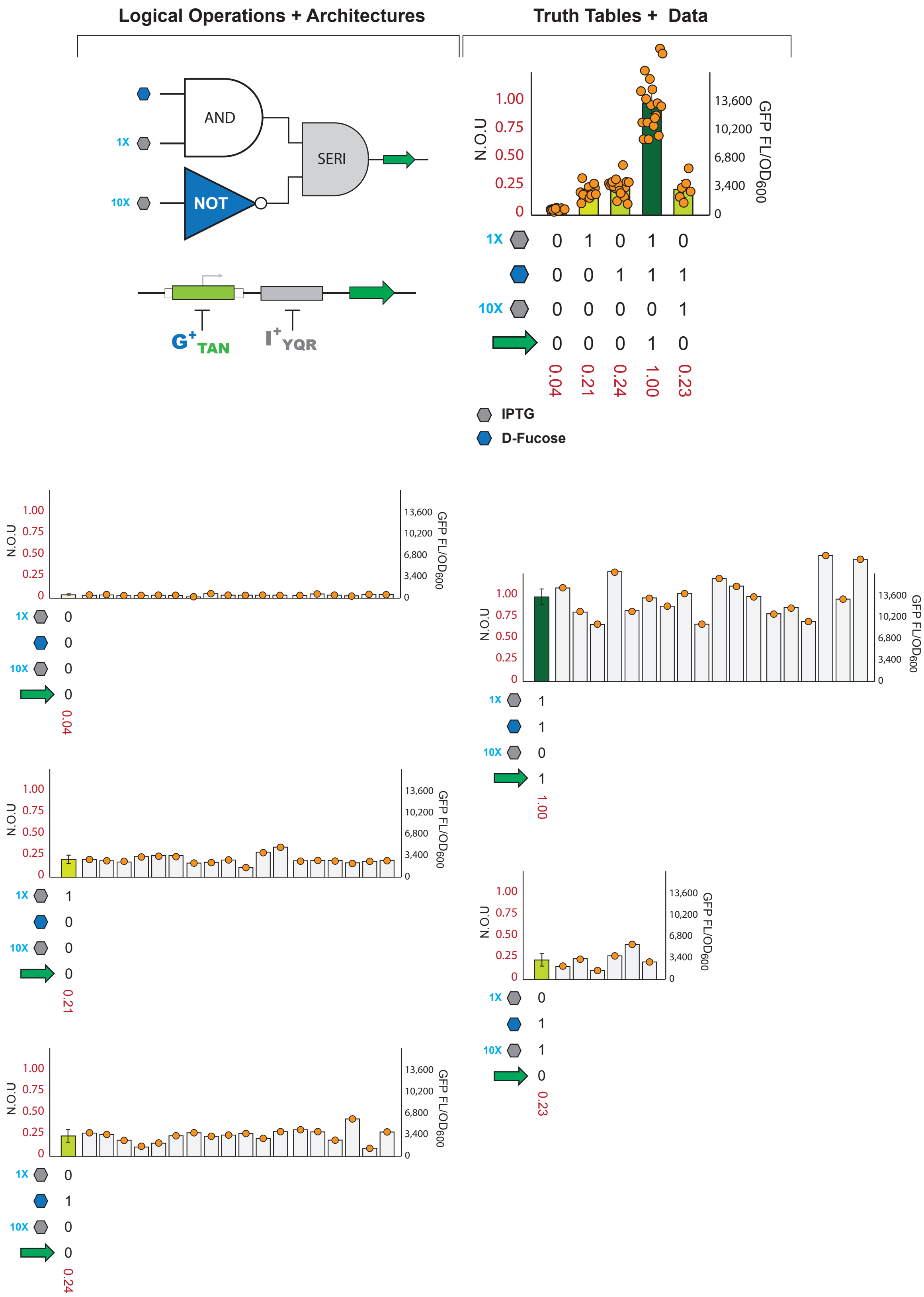

Supplementary Figure 8c

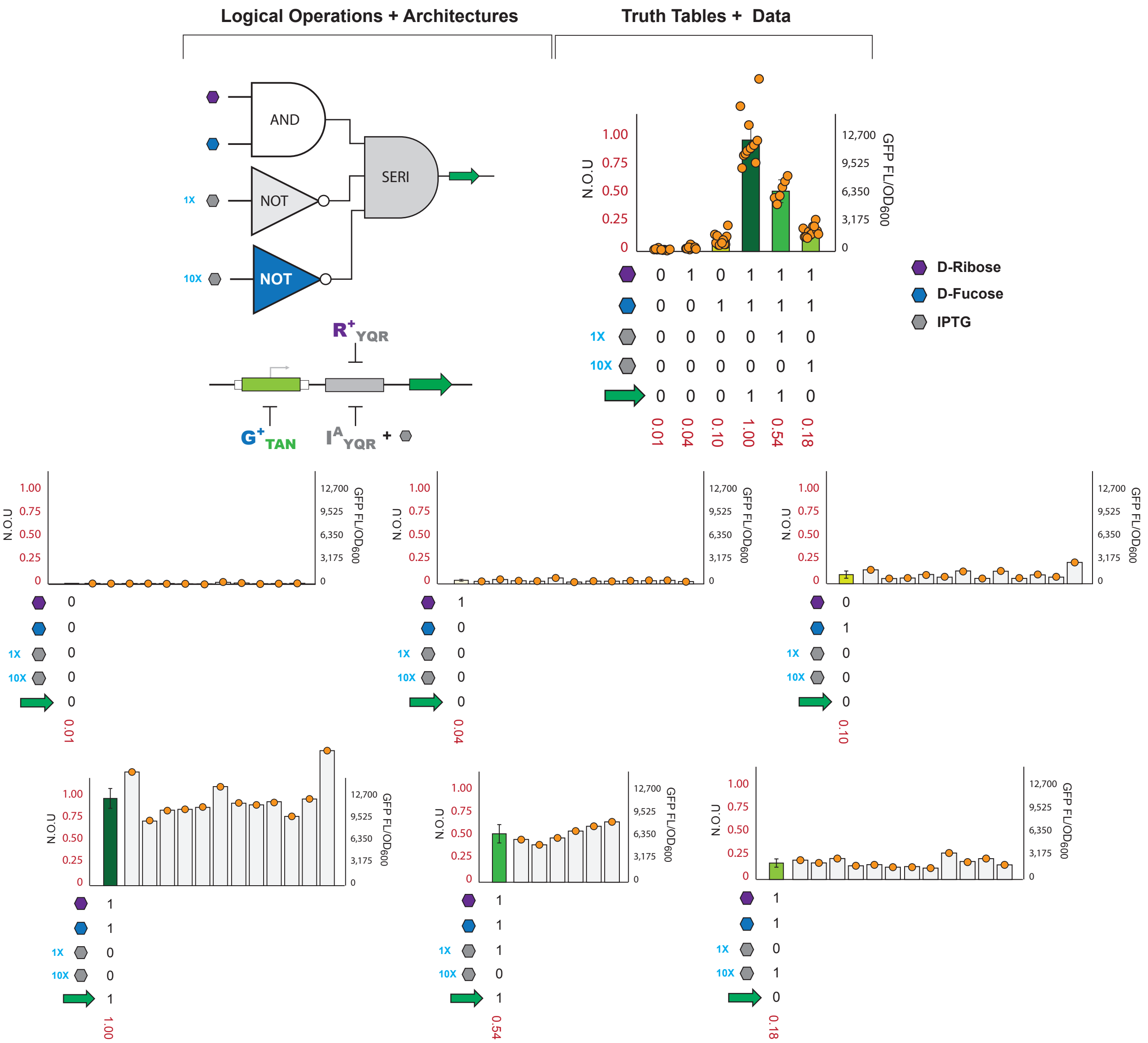

Supplementary Figure 8: Raw data SERI combinatorial logic gates. The second y-axis on the right shows the raw GFP Fluorescence/OD_{600} values that have been normalized to N.O.U. Y-axis (left) is given as normalized output units or N.O.U. This is calculated by taking GFP Fluorescence (485ex., 510em.) normalized to OD_{600}, which is further normalized to the maximum output for each experiment. Fractional expression values are shown in red under the truth tables. **(a)** IPTG acts as a competitive inhibitor to GalR, preventing GFP expression upon addition of fucose in the presence of 10mM IPTG. When 1mM IPTG is present, GalR is only slightly inhibited. **(b)** NOT logic function can be incorporated into the circuit by utilizing IPTG as a competitive inhibitor to GalR, resulting in an apparent ‘bandpass filter’, where GFP output is achieved only in the presence of certain IPTG concentrations. **(c)** A second NOT gate can be added by introducing an I^A transcription factor; this represents a more granular ‘bandpass filter’ where the output can be gradually decreased upon addition of different concentrations of IPTG. Complementary to **Fig. 6a-c**.

Supplementary Figure 9

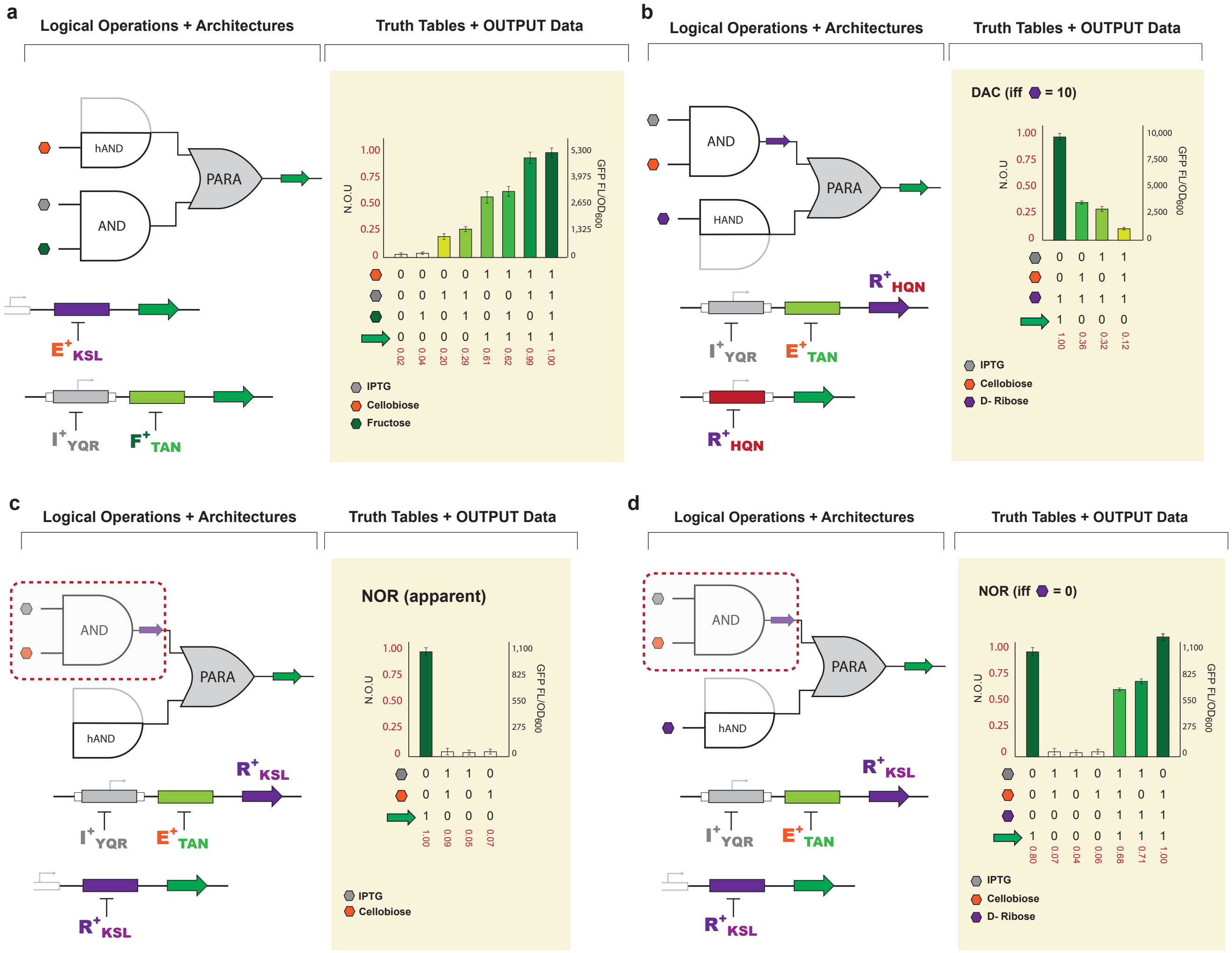

Supplementary Figure 9a

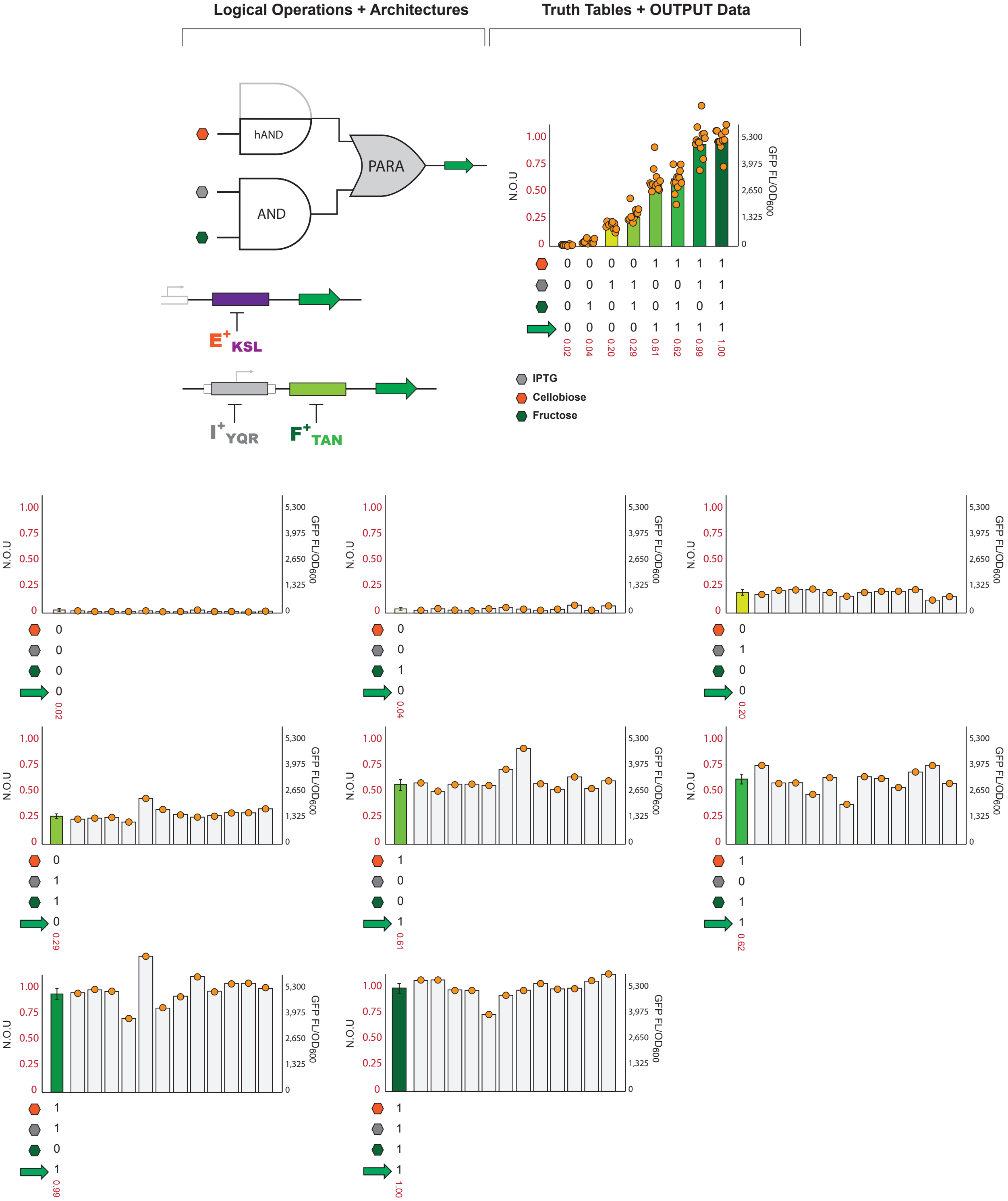

Supplementary Figure 9b (part 1)

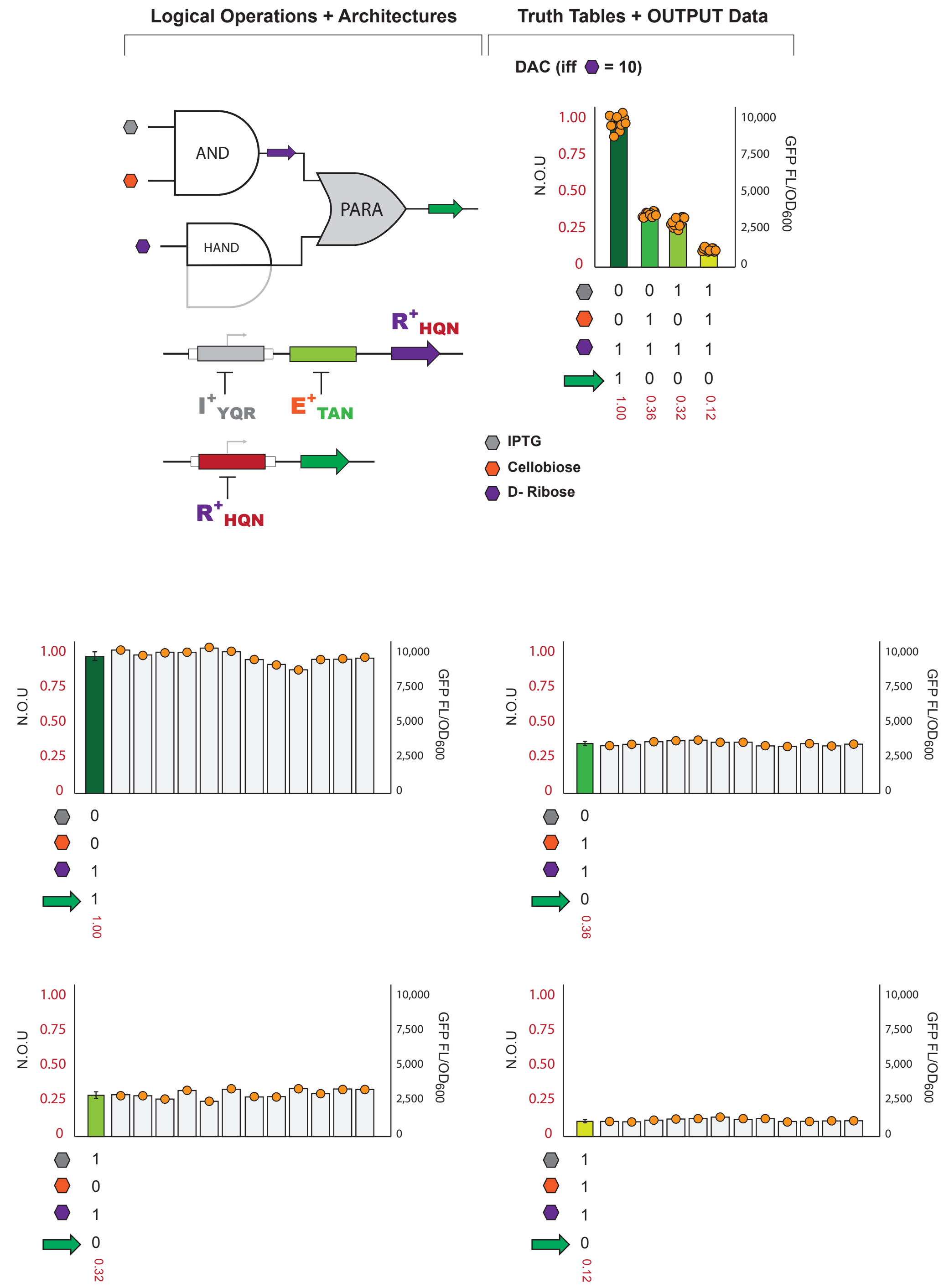

Supplementary Figure 9b (part 2)

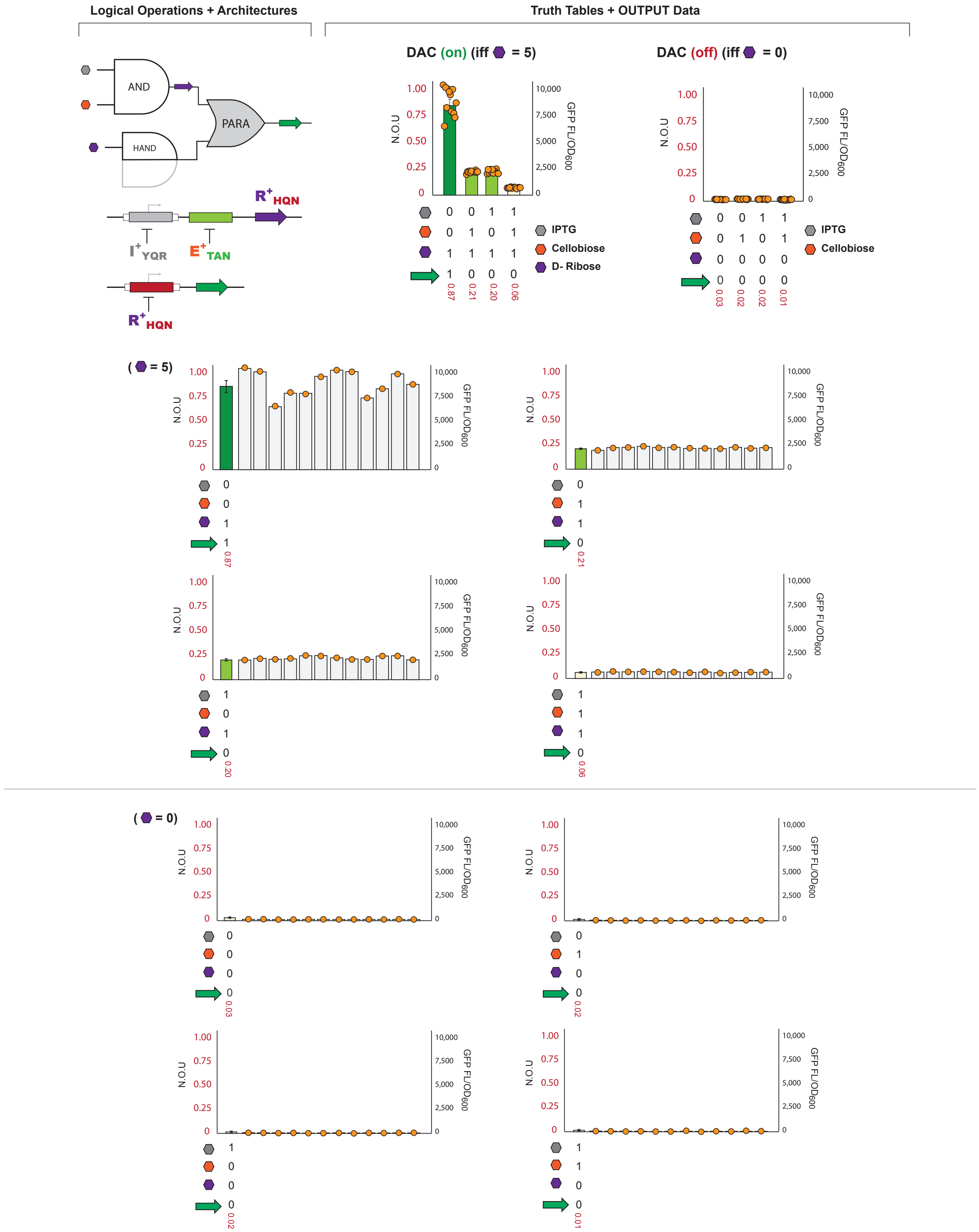

Supplementary Figure 9c

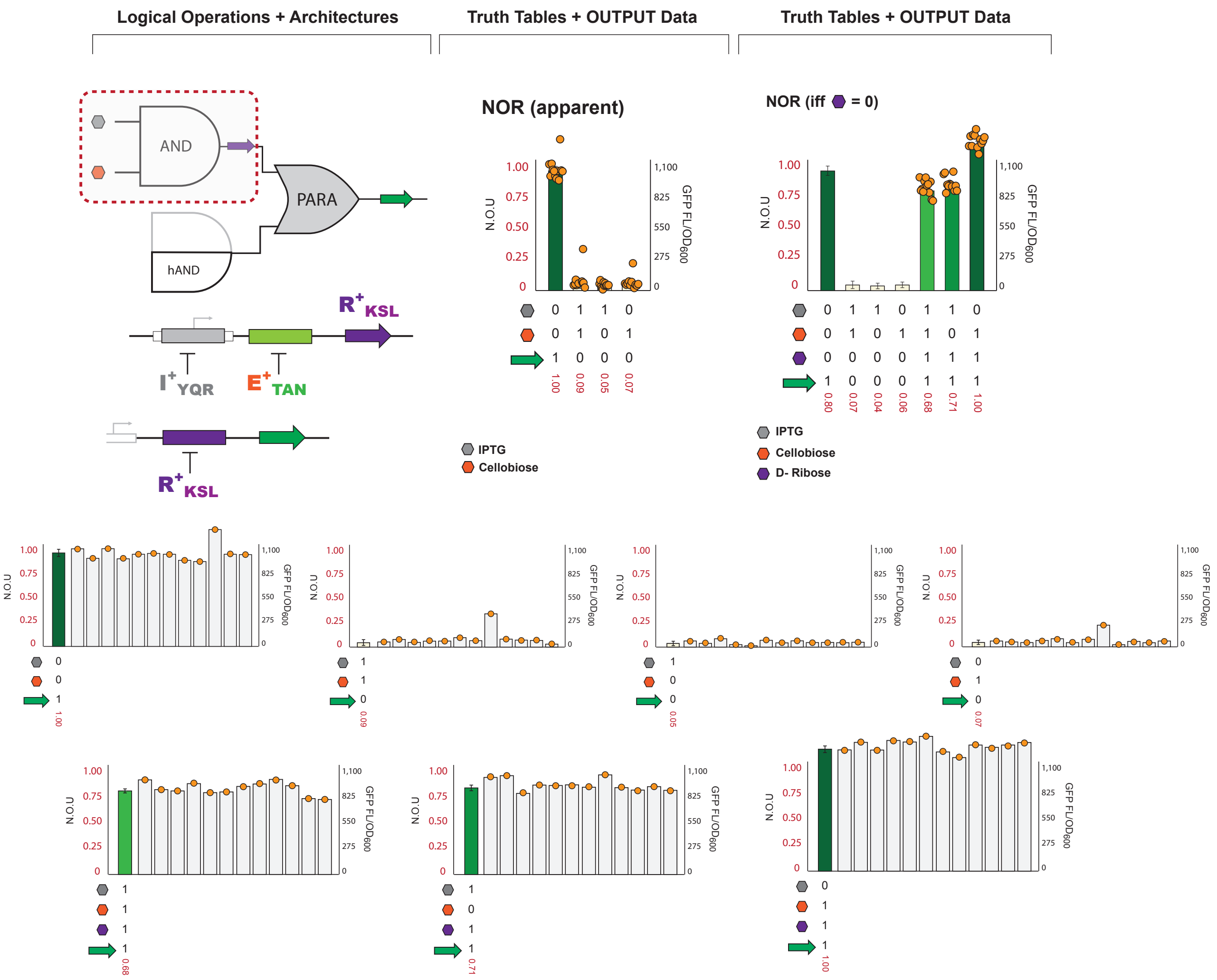

Supplementary Figure 9: Raw data for DAC and NOR circuits. The second y-axis on the right shows the raw GFP Fluorescence/OD_{600} values that have been normalized. The N.O.U. fractional expression values are shown in red under the truth tables. **(a)** Asymmetric parallel configuration composed of three non-synonymous DNA operators. The progressive addition of ligands (which represent digital inputs 0 or 1) leads to a gradual increase in GFP output. **(b)** A second asymmetric parallel system (denoted AND [PARA] HAND) where the two channels are coupled *via* an engineered TF-operator pair. The production of R^{+}_{HQN} is dependent on the addition of IPTG and Cellobiose such that progressive addition of inputs leads to a decrease in GFP output. 10mM ribose is required to alleviate repression **(c)** Slight modifications generate vastly different performance. In this iteration, R^{+}_{KSL} (interacting with a 'proximal' O^{acc}) replaces R^{+}_{HQN} (interacting with a 'core' O^{tgg}) and the change in performance characteristics leads to a more digital output profile, giving the appearance of a NOR gate. Red dashed box indicates a portion of the circuit that is influenced by the performance of the engineered transcription factor (*i.e.* the SERI operation behaves more like an OR rather than AND). **(d)** Upon addition of D-ribose, the NOR gate is deactivated such that the program becomes NOR if and only if (iff) there is no D-Ribose present. Complementary to **Fig. 7**.

Supplementary Figure 10

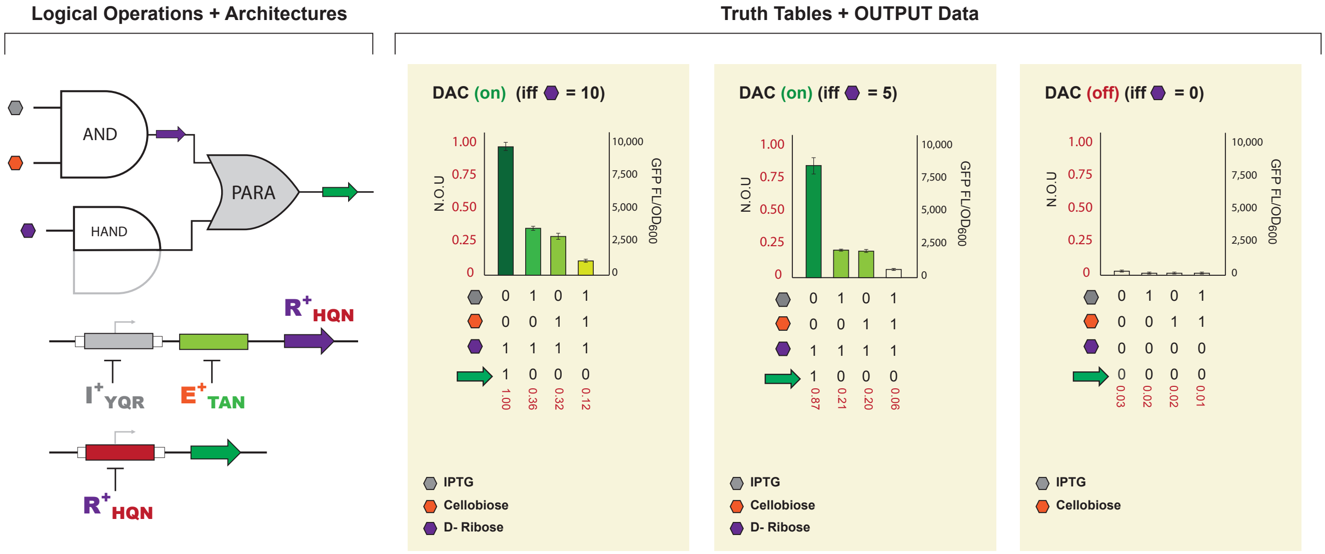

Supplementary Figure 10: Demonstration of the versatility of a DAC program. By replacing the inert output (GFP) with a ligand responsive transcription factor, we are capable of introducing additional layers of complexity. As seen in **Fig. 7b**, the production of increasing amounts of R^{+}_{HQN} *via* the addition of ligands cellobiose and IPTG leads to a decrease in GFP expression *if and only if* (iff) there is D-ribose present to alleviate the repression. By tuning the amount of D-ribose present (from 10mM to 5mM to 0mM), we are able to attenuate or even abolish the response of the system and the fluorescence observed. Complementary to **Fig. 7b**.

Supplementary Figure 11

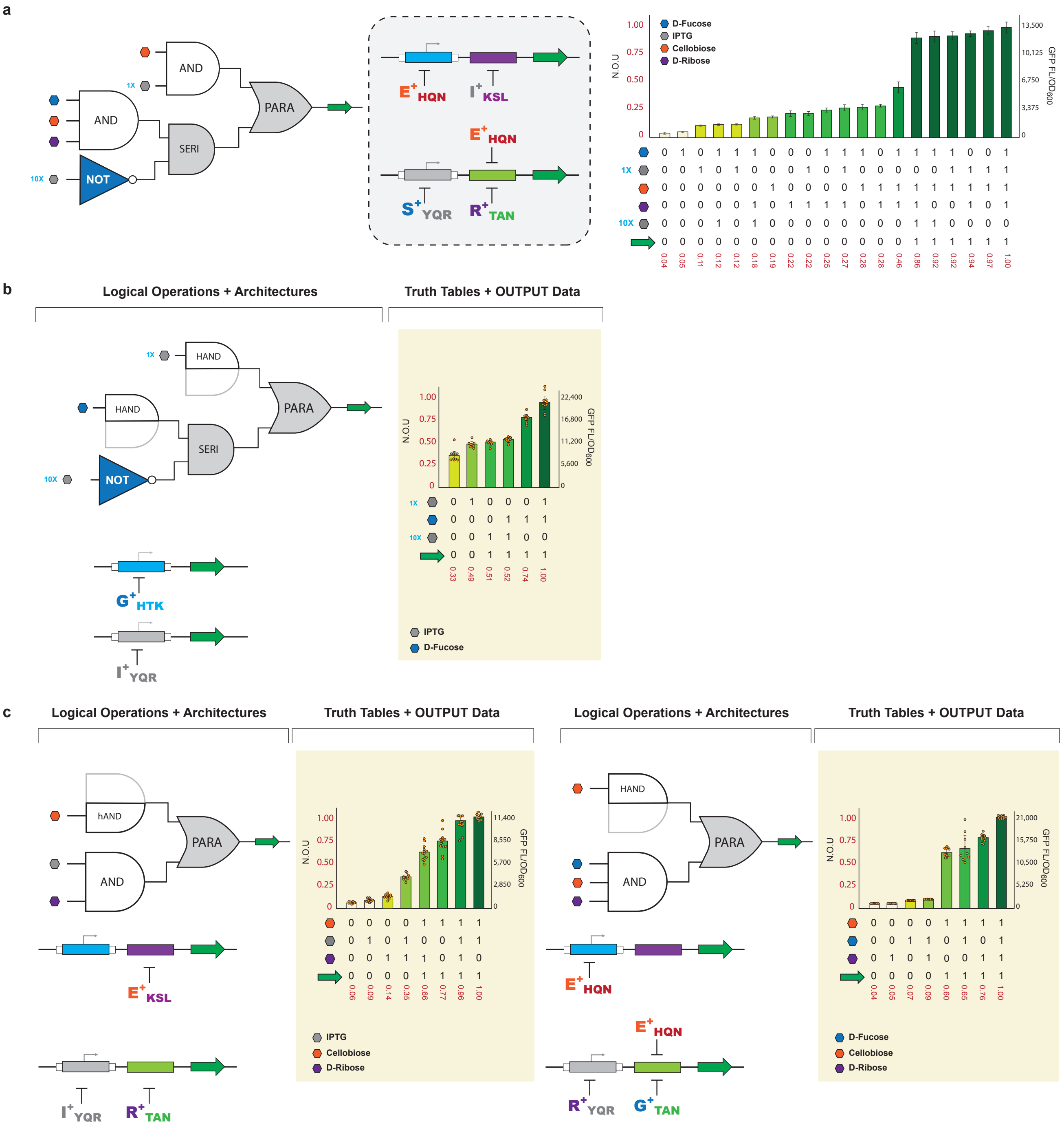

Supplementary Figure 11a (part 1)

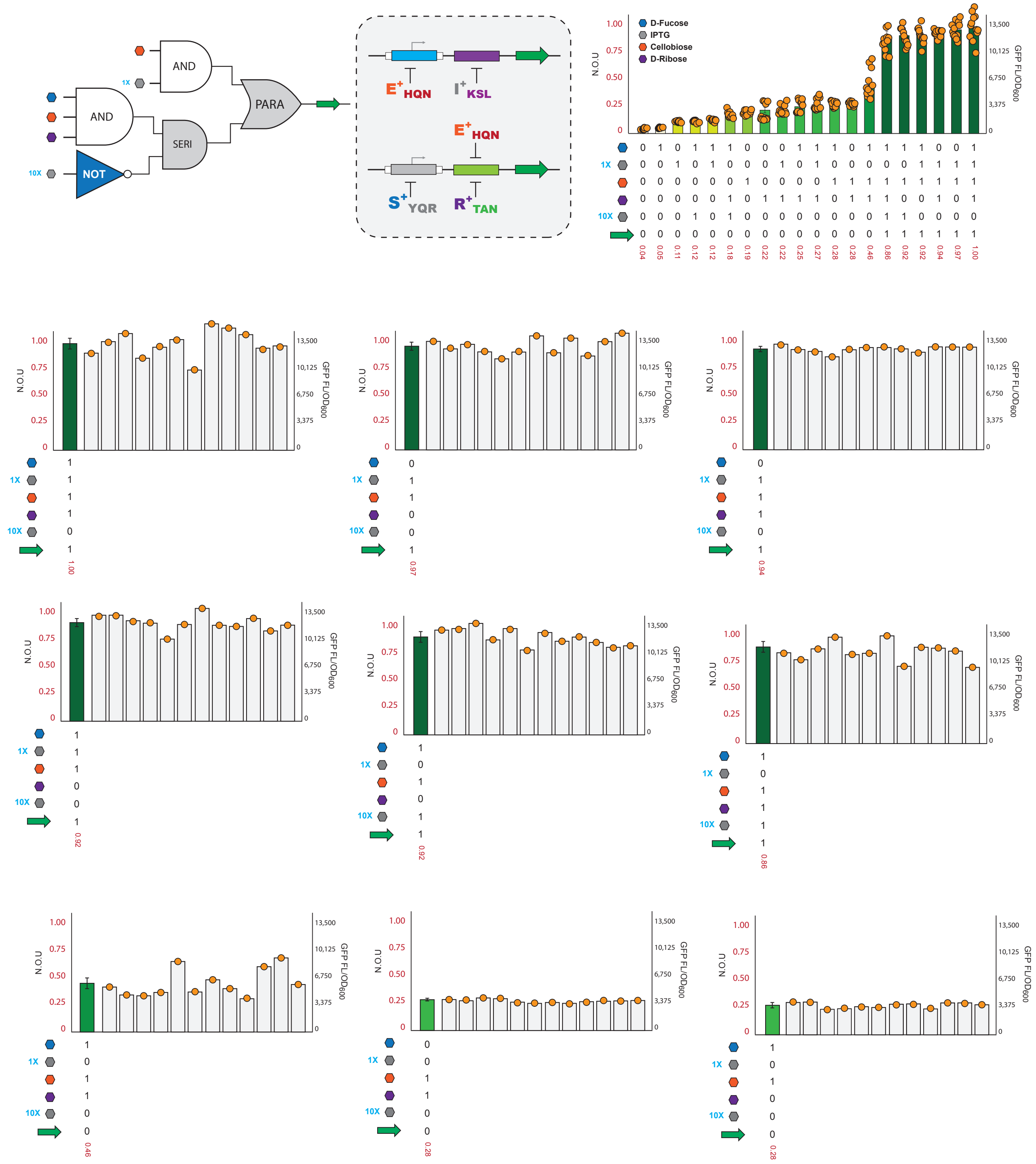

Supplementary Figure 11a (part 2)

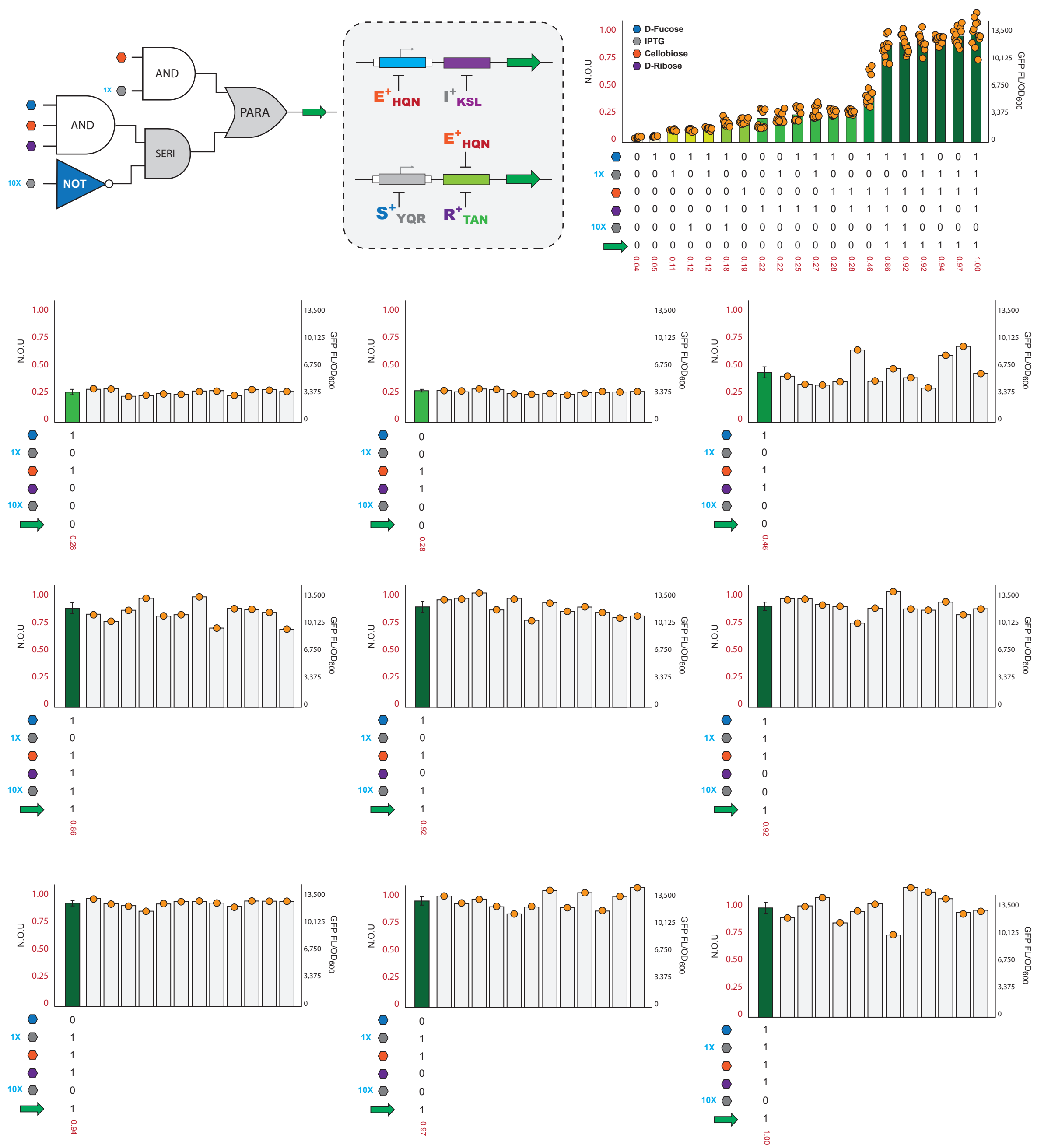

Supplementary Figure 11: The master circuit programs and raw data. (a) Arranging two SERI structures in a PARA configuration leads to the development of the genetic master circuit. This configuration employs four non-synonymous DNA operators and allows for the generation of any of the logical programs presented in this study. When both outputs are the same (GFP), this gives rise to the most granular digital-to-analog converter observed in this study. **(b)** This program leverages the same architecture of the master circuit seen above but utilizes only two transcription factors to exert control over GFP expression. G^{+}_{HTK} and I^{+}_{YQR} are used to control their cognate operators (O^{agg} and O^{sym}, respectively). Next, we leverage the ligand crossover between the two to increase an added layer of complexity - the NOT function at 10mM IPTG. **(c)** Two iterations of the 3 input DAC shown in the top figure. By utilizing distinct transcription factors, we can achieve distinct, discrete "states" or levels of GFP expression and we can also rever t between more "digital-like" (on the right) or "analog-like" (on the left) decision making. The second y-axis on the right shows the raw GFP Fluorescence/OD_{600} values that have been normalized the N.O.U.. Complementary to **Fig. 8**.

Supplementary Figure 12 (part 1)

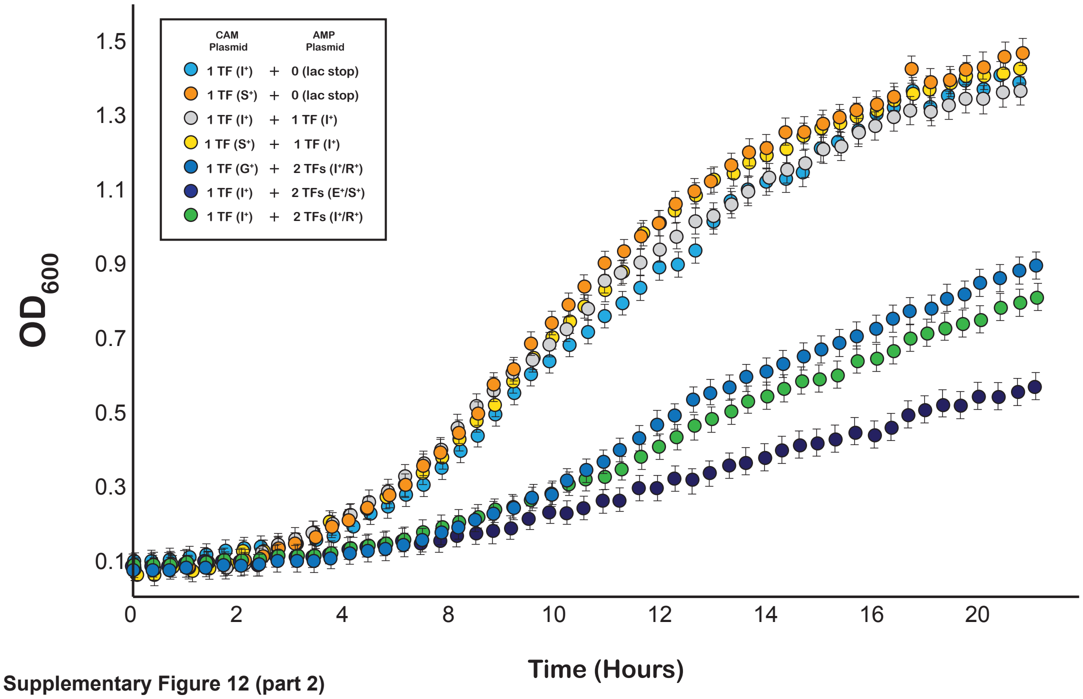

Supplementary Figure 12 (part 2)

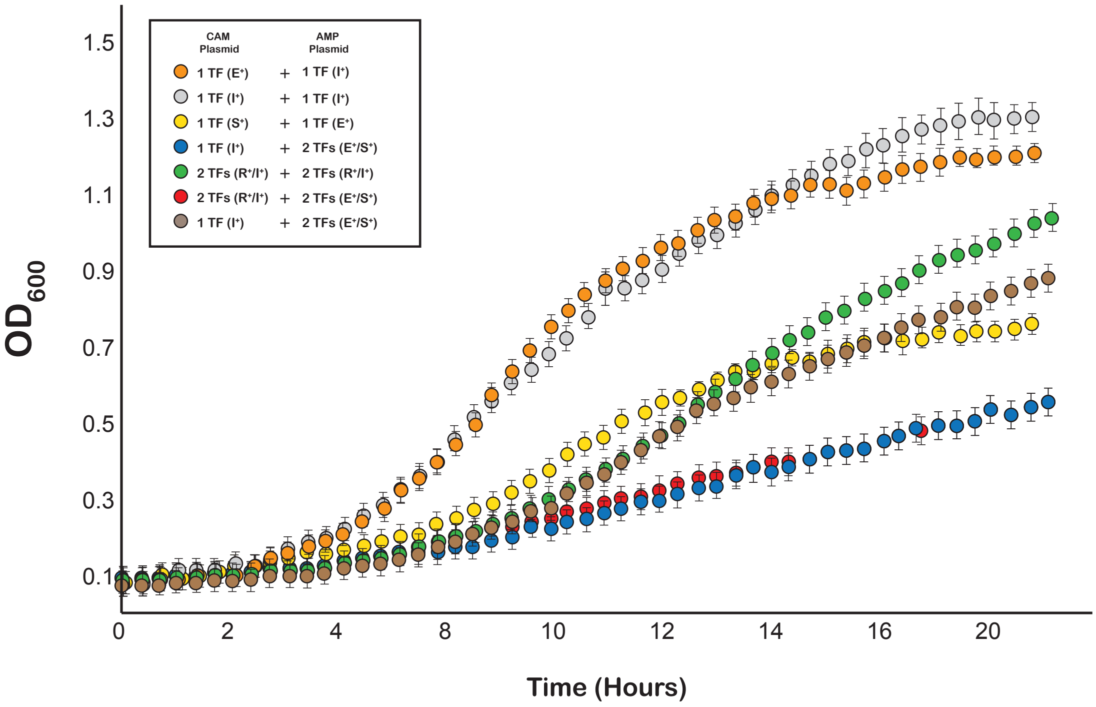

Supplementary Figure 12: Growth curves for several combinations of transcription factors. Cells were co-transformed with both the pLac (CAM resistant) plasmid and the pSO (AMP resistant) plasmid coding for either 0 (lac stop), 1, 2 , 3 or 4 transcription factors in the absence of GFP reporter. Cells were grown as outlined in the Methods section and the OD_{600} was sampled at regular intervals. Data points represent an average of n = 5 Biological Replicates taken on the same day and error bars indicate the standard deviation (SD). Also see **Supplementary Note 3**.

Supplementary Note 3: The GalS and GalR chimeras have previously been reported as toxic to *E. Coli* [2] without the incorporation of the E230K mutation and were therefore chosen for further experimentation. Although our S^{+}_{ADR} chimeras do contain the E230K mutation, we observe no toxicity when we express either the combination of S^{+} and LacStop or S^{+} and I^{+} (relative to I^{+} and LacStop and I^{+} and I^{+}, respectively). Conversely, our G^{+}_{ADR} variants do not contain the E230K mutation, but growth curves for several combinations reveal there is no toxicity when expressing either the G^{+} and LacStop, the G^{+} and I^{+} or the G+ and I+/R+ combinations (the latter was compared relative to the I^{+} and I^{+}/R^{+} combination, which contains no RCDs that have been reported as toxic). One interesting finding was that the combination of 3 TFs consisting of I^{+} and E^{+}/S^{+} showed a stark difference in growth when compared to the combination of G^{+} and I^{+}/R^{+} **(Supplementary Fig. 12).** Having established that GalS shows no toxicity to the cells, we hypothesized that CelR may be accounting for some of the differences in growth. The E^{+} and I^{+} combination shows a slight decrease in growth relative to the I^{+} and I^{+} combination, indicating some degree of toxicity, and this is exacerbated in the E^{+} and S^{+} combination. This combination also expresses E^{+} on the pSO plasmid, rather than the pLacI and the former expresses our Transcription Factor using the stronger lacIq promoter, rather than the lacI promoter, which would result in higher expression of our purportedly toxic E^{+} chimera. This toxicity is also seen in the combinations of R^{+}/I^{+} and E^{+}/S^{+} and I^{+} and E^{+}/S^{+}, both of which contain the combination of E^{+} and S^{+} with expression of E^{+} driven by the stronger lacIq promoter. It is interesting to note that CelR is the only Transcription Factor used in this work that does not originate in *E. Coli* but rather originates from *Thermobifida fusca*.

Supplementary Figure 13

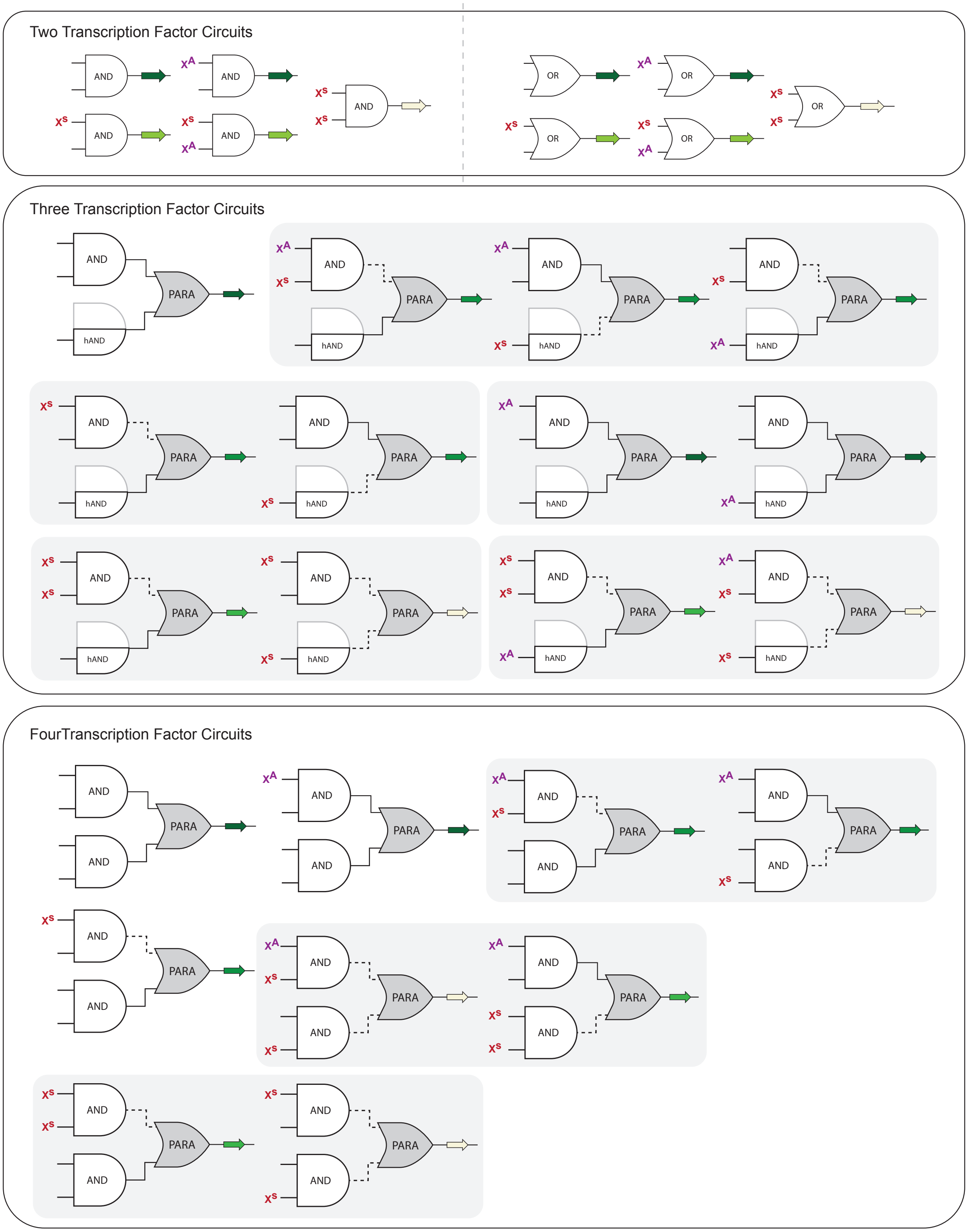

Supplementary Figure 13: Analog logic circuits that can be constructed from three transcription factor (TF) phenotypes for two-, three-, and four-operator architectures. Phenotype nomenclature is described in **Fig. 1**. Dashed output lines indicate attenuated gene expression due to the influence of a super repressor on that gene, as do lighter/yellower shades of green at the final output. Two-TF operator architectures are shown in main text **Fig. 3**. Examples of the three-TF and four-TF operator architectures are presented in the main text, **Fig. 7** and **Fig. 8** (respectively).

Supplementary Note 4: Combinatorial and Permutational Analyses. To better understand the number of unique TF/operator groupings enabled by our chimeric set of TFs, we have performed combinatorial and permutational analyses of the operator architectures presented in this paper (SERI, PARA, SERI [PARA] hAND, and the master circuit, SERI [PARA] SERI). For the purposes of these calculations, we consider only those circuits wherein (1) each TF interacts with only one operator, and no other TF interacts with that operator, and (2) only one gene is under control of these operators (even if there are multiple copies of that gene under control, as in the PARA architecture). The number of unique combinations for each operator architecture, phenotype selection, and starting assumptions is presented in **Supplementary Table 1**.

Using different starting assumptions allows us to focus on different aspects of the scale of possible circuit configurations using our chimeric TF set. A combinatorial analysis takes into account two additional assumptions: (1) that all TFs have equal repression strength, induction strength, and dynamic range, and (2) that operators all have equal repression strength. These assumptions reveal the number of uniquely ligand- and operator-orthogonal TF combinations that can be constructed for a given number of operator sites, without regard to resulting circuit performance. Permutational analysis A gives a better picture of the scope of the circuit design space because it takes into account differences in TF repression strength. Permutational analysis B takes into account both TF repression strength and operator repression strength, and these differences allow for tunable circuit performance and better represent the inherent variability in performance across unique TFs and operator positions.

Counting the number of unique TF/operator groupings from our dataset is achieved using a custom MATLAB script and set of helper functions available as **Supplementary Software 2**. The user sets the number of TFs/operators, which phenotypes to include in the calculation, and whether the calculation should be combinatorial or permutational. The function then steps through each possible combination of RCDs given that TF/operator number and the repression matrices fed in (the repression matrices from this current work (shown in **Fig. 2**) are fed in by default), and within those combinations compares individual TFs (*i.e.*, RCD + ADR + operator). The code rejects any non-orthogonal groupings, which can occur if the TFs bind to the same operator or the TFs respond to the same ligand. Accepted groupings are stored in a cell array, and the total number of rows in the cell array at the end of the calculation is the number of TF combinations given the input parameters.

The availability of three functional phenotypes in our dataset expands the range of analog circuit logic that can be constructed for each operator architecture. As shown in **Supplementary Fig. 13**, two-operator architectures (both SERI and PARA) allow for five basic analog logic operations, three-operator architectures of the type shown in main text **Fig. 7** allow for 12 basic analog logic operations, and four-operator architectures (SERI [PARA] SERI) allow for 9 basic analog logic operations. In addition, a much broader range of circuits with unique expression control and logic can be achieved by well-known techniques for fine-tuning circuit parameters, such as operator strengths, operator positions, plasmid copy number, distinct operators controlling different genes, translational feedback (of the type shown in main text **Fig. 7b-d**).

Supplementary Table 1.

# TFs/Operators	Condition	Combinatorial Count (TFs having equal parameters, operators having equal strength)	Permutational Count (TFs having different strengths, operators having equal strength)	Permutational Count (TFs having different strengths and operators having different strengths)
2	X^{+} only	365	485	970
2	X^{+} and X^{s}	434	594	1188
2	X^{+} and I^{A}	500	677	1354
2	X^{+}, X^{s}, I^{A}	578	808	1616
3	X^{+} only	909	1896	11376
3	X^{+} and X^{s}	1151	2645	15870
3	X^{+} and I^{A}	1451	3133	18798
3	X^{+}, X^{s}, I^{A}	1764	4241	25446
4	X^{+} only	655	2441	29292
4	X^{+} and X^{s}	854	3920	47040
4	X^{+} and I^{A}	1193	4553	54636
4	X^{+}, X^{s}, I^{A}	1492	7182	86184

Numbers of unique TF sets computed for different numbers of operators and different sets of phenotypes. The combinatorial count, permutational count A, and permutational count B are based on different assumptions related to TF and operator strength.

Supplementary Software 2 – Separate Documents.

Supplementary Data 1: Sequences for all primers used in this work both for synthesis and plasmid construction – Separate Excel Spreadsheet.

Supplementary Data 2: Statistical Data for all functional repressors including p-values and effect sizes (Cohen's d values) – Separate Excel Spreadsheet.

Source Data – Separate Documents.

REFERENCES

1. Daber, R. & Lewis, M. A novel molecular switch. *J Mol Biol* **391**, 661-670 (2009).
2. Meinhardt, S. *et al.* Novel insights from hybrid LacI/GalR proteins: family-wide functional attributes and biologically significant variation in transcription repression. *Nucleic Acids Res* **40**, 11139-11154 (2012).
