# Supplementary material for: Transcriptional programming using engineered systems of transcription factors and genetic architectures
Source: Nat Commun. 2019 Oct 21;10:4784. doi: 10.1038/s41467-019-12706-4 (PMC6803630; doi:10.1038/s41467-019-12706-4)
Supplement: Supplementary file 2 — Description of Additional Supplementary Files [file 41467_2019_12706_MOESM2_ESM.pdf]

Description of Additional Supplementary Files

File Name: Supplementary Data 1
Description: Sequences for all primers used in this work both for synthesis and plasmid construction – Separate Excel Spreadsheet.

File Name: Supplementary Data 2
Description: Statistical Data for all functional repressors including p-values and effect sizes (Cohen's d values) – Separate Excel Spreadsheet.

File Name: Supplementary Software 1
Description: Costume software used to calculate the combinatorial count in Supplementary Table 1. With installation guide source code, pseudocode and General Public License included.
