# Supplementary material for: Transcriptional programming using engineered systems of transcription factors and genetic architectures
Source: Nat Commun. 2019 Oct 21;10:4784. doi: 10.1038/s41467-019-12706-4 (PMC6803630; doi:10.1038/s41467-019-12706-4)
Supplement: Supplementary file 3 — Supplementary Software 1 [file 41467_2019_12706_MOESM3_ESM.zip › Supplementary Software/Installation Guide.docx]

The ‘.m’ files included in this file are for use in MATLAB. As usual for MATLAB, all of these .m files need to be in the working directory (called the ‘current folder’ in MATLAB) that the user sets in the leftmost window of MATLAB. Once this zipped file is extracted, in MATLAB the user can simply navigate to the folder containing these ‘.m’ files, and once there open the “TFCombinationsCount” and run the script. The user can also drag these files into whatever ‘current folder’ is set in MATLAB. Further details are given in the ‘readme’ file.

The typical install time for this script and helper functions is <1 second.

Please contact [corey.wilson@chbe.gatech.edu](corey.wilson@chbe.gatech.edu%20) with any questions.

Code available at <https://github.com/AndrewEShort/Wilson-Lab>
