# Supplementary material for: Transcriptional programming using engineered systems of transcription factors and genetic architectures
Source: Nat Commun. 2019 Oct 21;10:4784. doi: 10.1038/s41467-019-12706-4 (PMC6803630; doi:10.1038/s41467-019-12706-4)
Supplement: Supplementary file 3 — Supplementary Software 1 [file 41467_2019_12706_MOESM3_ESM.zip › Supplementary Software/readme.docx]

This file includes a MATLAB script (TFCombinationsCount) and a set of helper functions that allow the user to count orthogonal transcription factor (TF) combinations given a number of operator/TFs and a set of repression matrices (e.g. "Transcriptional programming using engineered systems of transcription factors and genetic architectures ", Figure 2). Any given repression matrices can be fed into the TFCombinationsCount script using GetTFsets.m, TfinputsRec.m, and XsInputsRec.m, though datasets that are not 7x7 will require the user to update and debug the code somewhat (see below).

**System Requirements:**

Software dependencies: The .m files will work in any functioning MATLAB install in any given operating system. The script and helper functions have been tested on MATLAB version 9.5.0.944444 (R2018b). No non-standard hardware is required.

**Installation Guide:**

The ‘.m’ files included in this file are for use in MATLAB. As usual for MATLAB, all of these .m files need to be in the working directory (called the ‘current folder’ in MATLAB) that the user sets in the leftmost window of MATLAB. Once this zipped file is extracted, in MATLAB the user can simply navigate to the folder containing these ‘.m’ files, and once there open the “TFCombinationsCount” and run the script. The user can also drag these files into whatever ‘current folder’ is set in MATLAB.

The typical install time for this script and helper functions is <1 second.

Please contact [corey.wilson@chbe.gatech.edu](file:///Users/cwilson323/Library/Containers/com.microsoft.Word/Data/Downloads/corey.wilson@chbe.gatech.edu%20) with any questions.

Code available at <https://github.com/AndrewEShort/Wilson-Lab>

**Demo:**

Open the TFCombinationsCount.m script in MATLAB. By default, this script is set up to count how many unique, orthogonal, four-transcription factor/operator circuits can be constructed from the dataset presented in Figure 2, along with a set of seven on-diagonal I^+^ phenotypes from *Rondon, R. E. and C. J. Wilson (2019). "Engineering a New Class of Anti-LacI Transcription Factors with Alternate DNA Recognition." Acs Synthetic Biology* ***8****(2): 307-317.* This count includes X^s^ phenotypes as well as I^A^ phenotypes, and assumes all TFs and operators have equal strength. When executed, after about 5 seconds this script is expected to return:

count =

1492

Changing “combinatorial” (line 8) = 0 and “permutational” (line 9) = 1 results in the script calculating two permutational counts, “count”, which tallies how many unique sets can be constructed if the TFs have different strenghts, and “countOp”, which tallies how many unique sets can be constructed if the TFs and operators have different strengths. After about 10 seconds, this modified script is expected to return:

count =

7182

countOp =

86184

These values correspond to those given in Supplementary Table 1.

**Instructions for use:**

Stepping through TFCombinationsCount, on line 5 the user chooses the number of TFs to include in all combinations considered in the calculation (currently configured for 2, 3 and 4 only; see details below), stored as numberOfTFs. 'Xs' designates whether to include the Xs phenotype in the calculation; 1 for yes, 0 for no. 'antilac' designates whether to include the antilac phenotype in the calculation; 1 for yes, 0 for no. 'Combinatorial' designates whether the calculation should reject variations in TF and operator repression strength (see below) (1 for yes, 0 for no). 'Permutational' designates whether the calculation should include variations in TF strength, and 'operatorDiff' in tandem with permutational designates whether the calculation should include variations in operator strength (see below) (1 for yes, 0 for no). Either combinatorial or permutational, but not both, should be set to 1.

The 'TFinputsRec' helper function feeds in repression matrix data. Functional phenotypes are input as '1's, nonfunctional as '0's. These inputs can be customized for any repression matrices (so long as the user is prepared to debug simple errors this causes in the code, since it's customized to 7x7 matrices for this study).

The 'XsInputsRec' helper function adds in Xs phenotypes to the repression matrices.

The 'GetTFsets' helper function feeds in the combinatorial groupings of numberOfTFs TFs. These were solved by hand and entered manually, and these groupings reject TFs that are inducible by the same ligand. This can be extended for any number of TFs, but currently only allows for groupings of 2, 3, or 4 TFs.

The code then steps through each repression matrix entry in the first TF from TFset from GetTFsets and compares it to every repression matrix entry in the second TF from TFset. The code rejects any non-orthogonal groupings which can occur if the TFs bind to the same operator (i.e. if any of the entries on their respective columns overlap by row). Accepted groupings are stored in a 3D cell array with each layer specific to a TFsets entry, and the total number of rows in the cell array at the end of the calculation is the number of TF combinations given the input parameters. Further details are annotated in the code.

**Pseudocode**:

- User sets solution parameters (TF number, combinatorial vs. permutational, and phenotypes to include).
- TFCombinationsCount calls either TFinputsRec or XsInputsRec depending on user-defined settings to define TF repression matrix variables (matrices of 1s and 0s, where 1s represent functional phenotypes and 0s represent nonfunctional phenotypes).
- TFCombinationsCount calls GetTFsets to define TFsets, a cell array containing the combinatorial groupings of compatible TFs.
- Enter for loop: for h = 1:RTFsets
  - For every TF grouping in TFsets, this for loop counts the number of possible TF combinations from their repression matrices as follows:
  - The indices of each functional phenotype for each TF are stored in TFACoords, TFBCoords, etc.
  - Starting from the lowest funcitonal indices, the first TF from currTFcombination is checked for how many rows it “rejects”, that is, how many and which operators are bound by that TF with that DNA binding domain. The coordinates are stored in ledger column 1, and the rows rejected based on that TF’s DNA binding domain promiscuity are stored in ledger column 2.
  - Based on ledger column 2, the first row that is not blocked and which contains a functional phenotype in TF set 2 from currTFcombination is checked for how many rows it rejects; these coordinates are stored in ledger columns 3 and 4, respectively.
  - After all pairs from the first two entries in all TF combinations from currTFcombination have been recorded, if 3 TF sets are being counted, AdditionalTFcontribution3 is called, and the third TF’s contribution is added to the ledger in a similar manner. If 4 TF sets are being counted, AdditionalTFcontribution4 is also called, and the fourth TF is added to the ledger in a similar manner.
  - Next, if the count is combinatorial, the ledger is searched for and purged of repetitions (e.g., in a combinatorial count, {[1,1], [2,2], [3,3]} is equivalent to {[1,1], [3,3], [2,2]}, so only one of each combinatorial set is kept in the ledger).
  - The above process is repeated for every combination in (every row of) TFsets, and every new ledger page is stored in the third dimension of the Ledger cell array (indexed by h, corresponding to the rows of TFsets).
- Exit for loop.
- The total number of rows in all pages of the ledger is counted, and the final count is returned. If the count is permutational, the count assuming all TFs have different strength but operators have equals strength is given as count, and the count assuming both TFs and operators have different strengths is given as countOp (where countOp = count * 2 for two-TF sets, countOP = count * 6 for three-TF sets, and countOP = count * 12 for four-TF sets (corresponding to how many unique TF positions there are given a three-operator PARA setup like Fig. 5a, or a four-operator PARA setup like Fig. 5e).

**Some further details from the Supplementary Information of "Transcriptional programming using engineered systems of transcription factors and genetic architectures" (see SI for associated figures and table).**

To better understand the number of unique TF/operator groupings enabled by our chimeric set of TFs, we have performed combinatorial and permutational analyses of the operator architectures presented in this paper (SERI, PARA, SERI [PARA] hAND, and the master circuit, SERI [PARA] SERI). For the purposes of these calculations, we consider only those circuits wherein (1) each TF interacts with only one operator, and no other TF interacts with that operator, and (2) only one gene is under control of these operators (even if there are multiple copies of that gene under control, as in the PARA architecture). The number of unique combinations for each operator architecture, phenotype selection, and starting assumptions is presented in Supplementary Table 1.

Using different starting assumptions allows us to focus on different aspects of the scale of possible circuit configurations using our chimeric TF set. A combinatorial analysis takes into account two additional assumptions: (1) that all TFs have equal repression strength, induction strength, and dynamic range, and (2) that operators all have equal repression strength. These assumptions reveal the number of uniquely ligand- and operator-orthogonal TF combinations that can be constructed for a given number of operator sites, without regard to resulting circuit performance. Permutational analysis A gives a better picture of the scope of the circuit design space because it takes into account differences in TF repression strength. Permutational analysis B takes into account both TF repression strength and operator repression strength, and these differences allow for tunable circuit performance and better represent the inherent variability in performance across unique TFs and operator positions.

Counting the number of unique TF/operator groupings from our dataset is achieved using a custom MATLAB script and set of helper functions available as Supplementary Software 2. The user sets the number of TFs/operators, which phenotypes to include in the calculation, and whether the calculation should be combinatorial or permutational. The function then steps through each possible combination of RCDs given that TF/operator number and the repression matrices fed in (the repression matrices from this current work (shown in main text Figure 2) are fed in by default), and within those combinations compares individual TFs (i.e., RCD+ADR+operator). The code rejects any non-orthogonal groupings, which can occur if the TFs bind to the same operator or the TFs respond to the same ligand. Accepted groupings are stored in a cell array, and the total number of rows in the cell array at the end of the calculation is the number of TF combinations given the input parameters.

The availability of three functional phenotypes in our dataset expands the range of analog circuit logic that can be constructed for each operator architecture. As shown in Supplementary Figure 13, two-operator architectures (both SERI and PARA) allow for five basic analog logic operations, three-operator architectures of the type shown in main text Figure 7 allow for 12 basic analog logic operations, and four-operator (main text Figure 8) architectures (SERI [PARA] SERI) allow for 9 basic analog logic operations. In addition, a much broader range of circuits with unique expression control and logic can be achieved by well-known techniques for fine-tuning circuit parameters, such as operator strengths, operator positions, plasmid copy number, distinct operators controlling different genes, translational feedback (of the type shown in main text Figure 7b-d).
