# Supplementary material for: Transcriptional programming using engineered systems of transcription factors and genetic architectures
Source: Nat Commun. 2019 Oct 21;10:4784. doi: 10.1038/s41467-019-12706-4 (PMC6803630; doi:10.1038/s41467-019-12706-4)
Supplement: Supplementary file 6 — Reporting Summary [file 41467_2019_12706_MOESM6_ESM.pdf]

Corresponding author(s): Corey J. Wilson

Last updated by author(s): Aug 18, 2019

Reporting Summary

Nature Research wishes to improve the reproducibility of the work that we publish. This form provides structure for consistency and transparency in reporting. For further information on Nature Research policies, see Authors & Referees and the Editorial Policy Checklist .

Statistics

For all statistical analyses, confirm that the following items are present in the figure legend, table legend, main text, or Methods section.

n/a	Confirmed	
☐	☒	The exact sample size (*n*) for each experimental group/condition, given as a discrete number and unit of measurement
☐	☒	A statement on whether measurements were taken from distinct samples or whether the same sample was measured repeatedly
☐	☒	The statistical test(s) used AND whether they are one- or two-sided<br>*Only common tests should be described solely by name; describe more complex techniques in the Methods section.*
☒	☐	A description of all covariates tested
☒	☐	A description of any assumptions or corrections, such as tests of normality and adjustment for multiple comparisons
☐	☒	A full description of the statistical parameters including central tendency (e.g. means) or other basic estimates (e.g. regression coefficient) AND variation (e.g. standard deviation) or associated estimates of uncertainty (e.g. confidence intervals)
☐	☒	For null hypothesis testing, the test statistic (e.g. *F*, *t*, *r*) with confidence intervals, effect sizes, degrees of freedom and *P* value noted<br>*Give P values as exact values whenever suitable.*
☒	☐	For Bayesian analysis, information on the choice of priors and Markov chain Monte Carlo settings
☒	☐	For hierarchical and complex designs, identification of the appropriate level for tests and full reporting of outcomes
☐	☒	Estimates of effect sizes (e.g. Cohen's *d*, Pearson's *r*), indicating how they were calculated

Our web collection on statistics for biologists contains articles on many of the points above.

Software and code

Policy information about availability of computer code

Data collection	MATLAB version 9.5.0.944444 (R2018b) was used to develop the custom code given in Supplemental Code 1.<br>BioTek Gen 5 installation version 2.07.17 was used for plate reader assays.
Data analysis	Microsoft Excel

For manuscripts utilizing custom algorithms or software that are central to the research but not yet described in published literature, software must be made available to editors/reviewers. We strongly encourage code deposition in a community repository (e.g. GitHub). See the Nature Research guidelines for submitting code & software for further information.

Data

Policy information about availability of data

All manuscripts must include a data availability statement. This statement should provide the following information, where applicable:

- Accession codes, unique identifiers, or web links for publicly available datasets
- A list of figures that have associated raw data
- A description of any restrictions on data availability

The authors declare that all analyzed data supporting the findings of this study are available within the paper [and its supplementary information files]. However, the raw dataset can also be made available from the corresponding author upon reasonable request. The sequences of the following plasmids and strains are provided in GenBank: CelR Variants (Accession #s MN207910 - MN207915), FruR Variants (MN207916 - MN207921), GalR Variants (MN207922 - MN207928), GalS Variants (MN207929 - MN207935), RbsR Variants (MN207958 - MN207963) Antilac Variants (MN207936 - MN207944), Master Architecture (MN207945), RbsR Reporters (MN207946, MN207947), pSO plasmid variants (MN207948 - MN207951), pSOx2 plasmids (MN207952 - MN207954), pLac-Lac plasmids (MN207955 - MN207957), Single Reporter Variants (MN207964 - MN207971), Series Reporter (MN207972)

Field-specific reporting

Please select the one below that is the best fit for your research. If you are not sure, read the appropriate sections before making your selection.

☒ Life sciences ☐ Behavioural & social sciences ☐ Ecological, evolutionary & environmental sciences

For a reference copy of the document with all sections, see nature.com/documents/nr-reporting-summary-flat.pdf

Life sciences study design

All studies must disclose on these points even when the disclosure is negative.

Sample size	In our previous work in ACS Synthetic Biology (Rondon and Wilson), we used a sample size of n=6 to determine the phenotype of the previously established IAYQR as well as the IAADR developed in that study. Throughout the study we found that with a sample size of 6 biological replicates, that we were able to faithfully replicate the results from our previous work in ACS Synthetic Biology (Richards et. al) and that we were also able to replicate the phenotypes found for the novel IAADR across measurements made on diferent days. For this current work, we chose to increase our sampling to n=12 to increase the power of our statistical hypothesis testing.
Data exclusions	No data was excluded from analysis - all 12 biological replicates were considered in the calculation of each data point
Replication	In filling out the Repression Matrices on figure 2, the 12 replicates were collected on two separate days with 6 points collected on each. In comparing the two sets of replicates, we found that the phenotypes and GFP output in the on/off state could be replicated within the confidence interval of the measurements.
Randomization	Colony Forming Units of cells containing the respective Genetic Circuits and Transcription Factors were chosen at random for assaying.
Blinding	Blinding is not feasible in this study because it is necessary for the authors to know the exact genotype and sequence of the plasmids being introduced into cells in order to properly set up the experimental conditions and assay.

Reporting for specific materials, systems and methods

We require information from authors about some types of materials, experimental systems and methods used in many studies. Here, indicate whether each material, system or method listed is relevant to your study. If you are not sure if a list item applies to your research, read the appropriate section before selecting a response.

Materials & experimental systems

n/a	Involved in the study	
☒	☐	Antibodies
☒	☐	Eukaryotic cell lines
☒	☐	Palaeontology
☒	☐	Animals and other organisms
☒	☐	Human research participants
☒	☐	Clinical data

Methods

n/a	Involved in the study	
☒	☐	ChIP-seq
☒	☐	Flow cytometry
☒	☐	MRI-based neuroimaging
